# Supplementary material for: Design, Synthesis, and Photophysical Properties of 5-Aminobiphenyl Substituted [1,2,4]Triazolo[4,3-c]- and [1,2,4]Triazolo[1,5-c]quinazolines
Source: Molecules. 2024 May 24;29(11):2497. doi: 10.3390/molecules29112497 (PMC11173969; doi:10.3390/molecules29112497)
Supplement: Supplementary file 1 [file molecules-29-02497-s001.zip › molecules-2995880-supplementary.pdf]

Design, synthesis and photophysical properties of 5-aminobiphenyl substituted [1,2,4]triazolo[4,3-*c*]- and [1,2,4]triazolo[1,5-*c*]quinazolines

Tatyana N. Moshkina, Alexandra E. Kopotilova, Marya A. Ivan'kina, Ekaterina S. Starnovskaya, Denis A. Gazizov , Emiliya V. Nosova , Dmitry S. Kopchuk, Oleg S. El´tsov, Pavel A. Slepukhin, and Valery N. Charushin

CONTENTS

1. NMR and mass spectra of 5-aryl-substituted [1,2,4]triazolo[4,3-*c*]quinazolines and [1,2,4]triazolo[1,5-*c*]quinazolines 2
2. The proposed reaction mechanism 39
3. Crystallographic data of triazoloquinazolines 40
4. Absorption and emission spectra of fluorophores in toluene and MeCN 45
5. Solvatochromic study for compounds **4** and **5** 54
6. Cyclic voltammograms for compounds **4a-f** and **5a-f** 61
7. Theoretical calculations 63

1. NMR and mass spectra of 5-aryl-substituted [1,2,4]triazolo[4,3-*c*]quinazolines and [1,2,4]triazolo[1,5-*c*]quinazolines

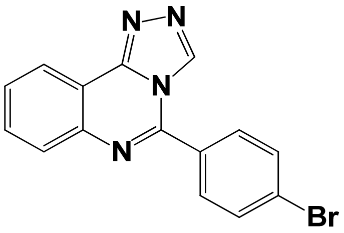

2a

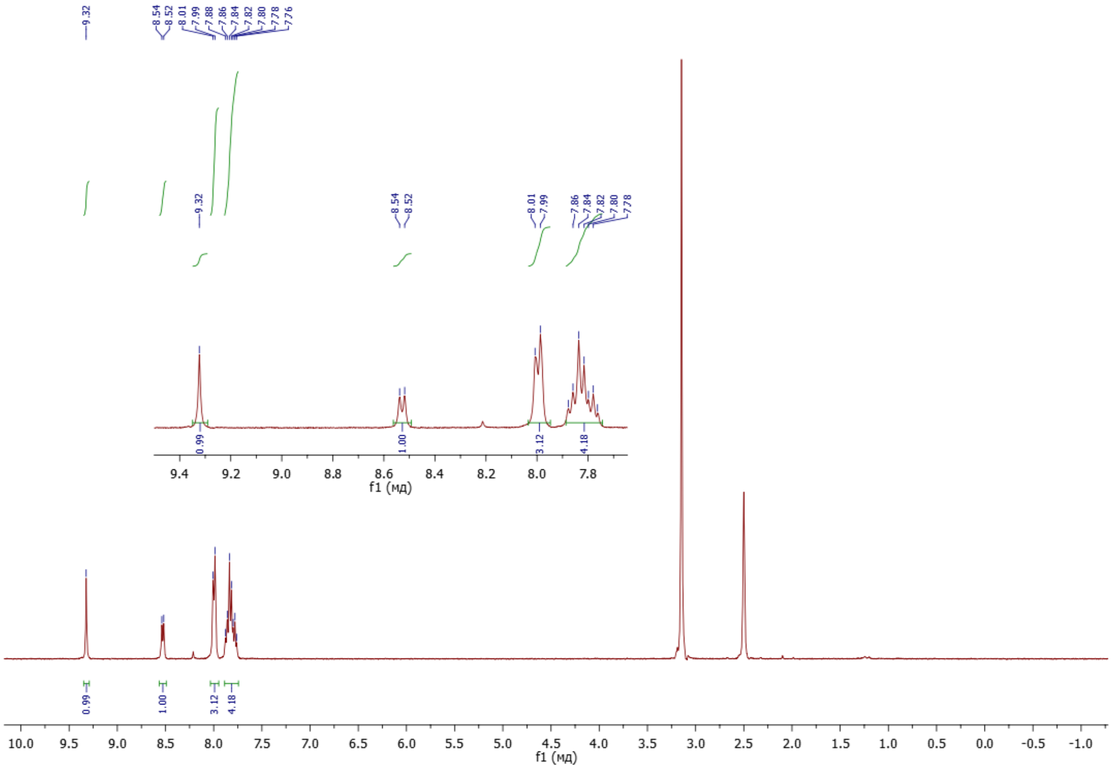

a

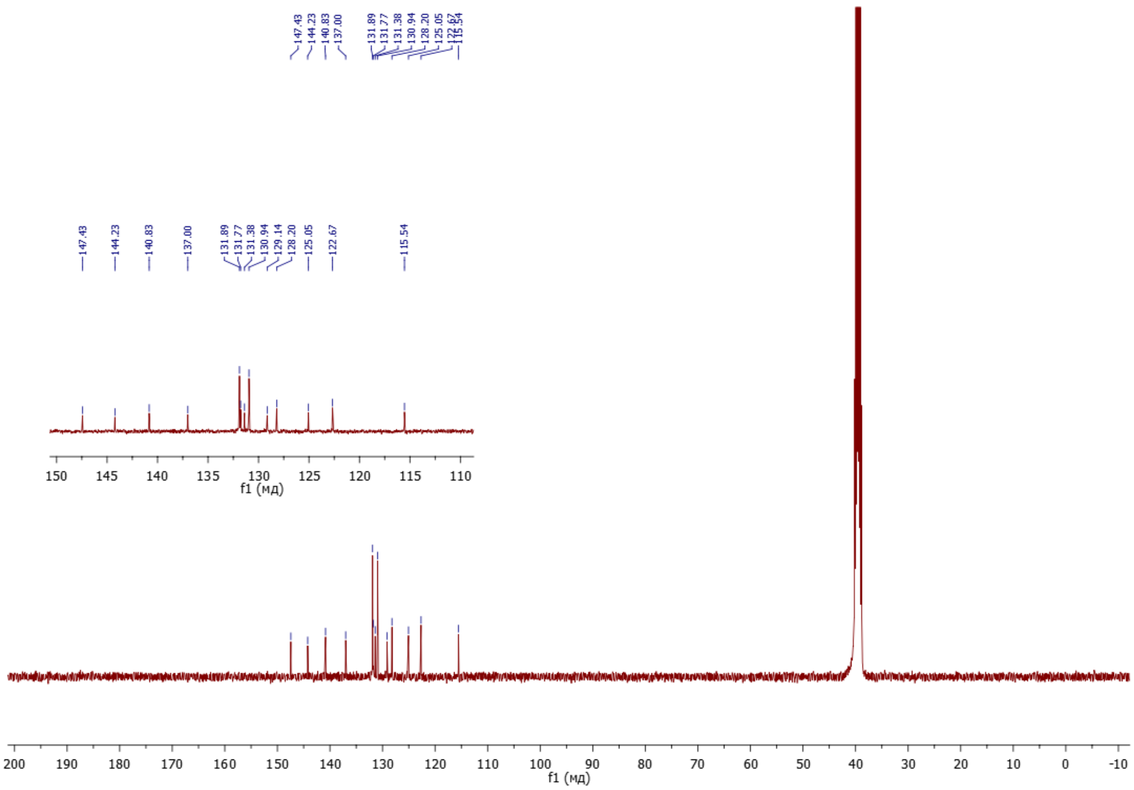
147.43
144.23
140.83
137.00
131.89
131.77
131.38
130.94
129.14
128.20
125.05
122.67
115.54
f1 (мд)

b

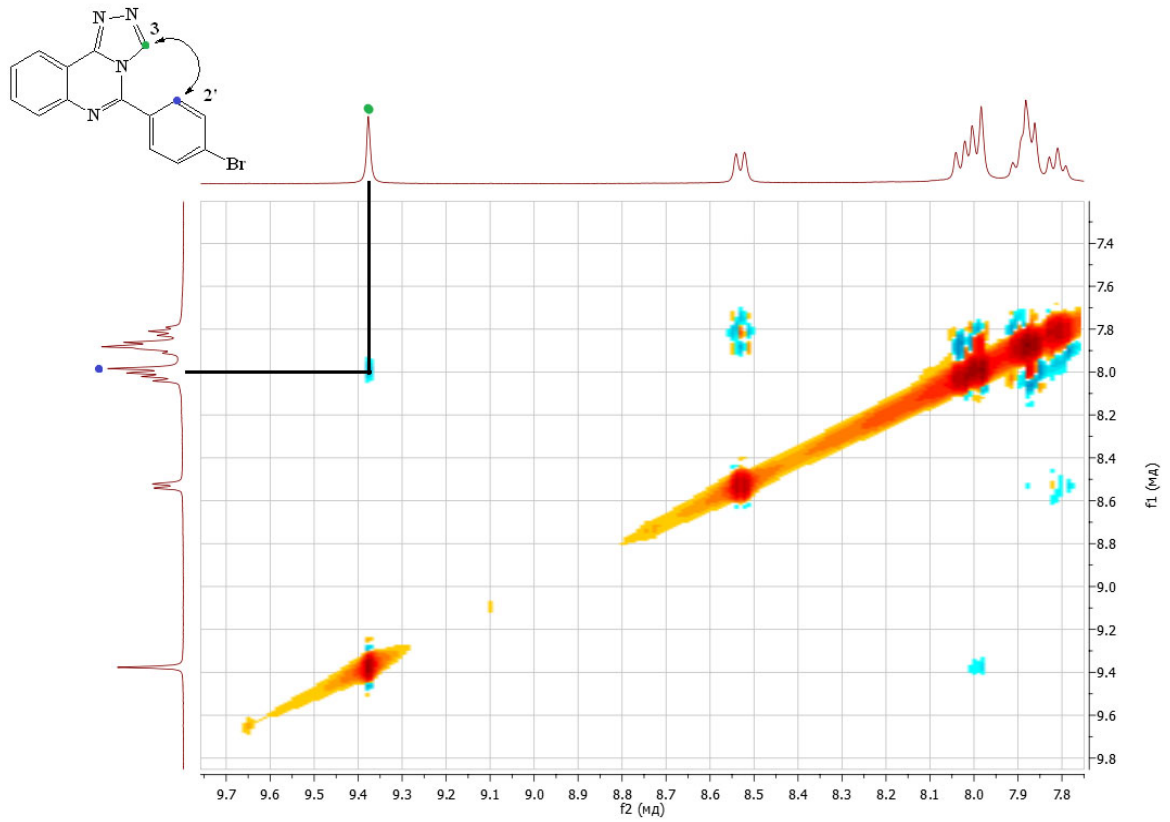
N
N
3
N
2'
N
Br
f2 (мд)
f1 (мд)

c

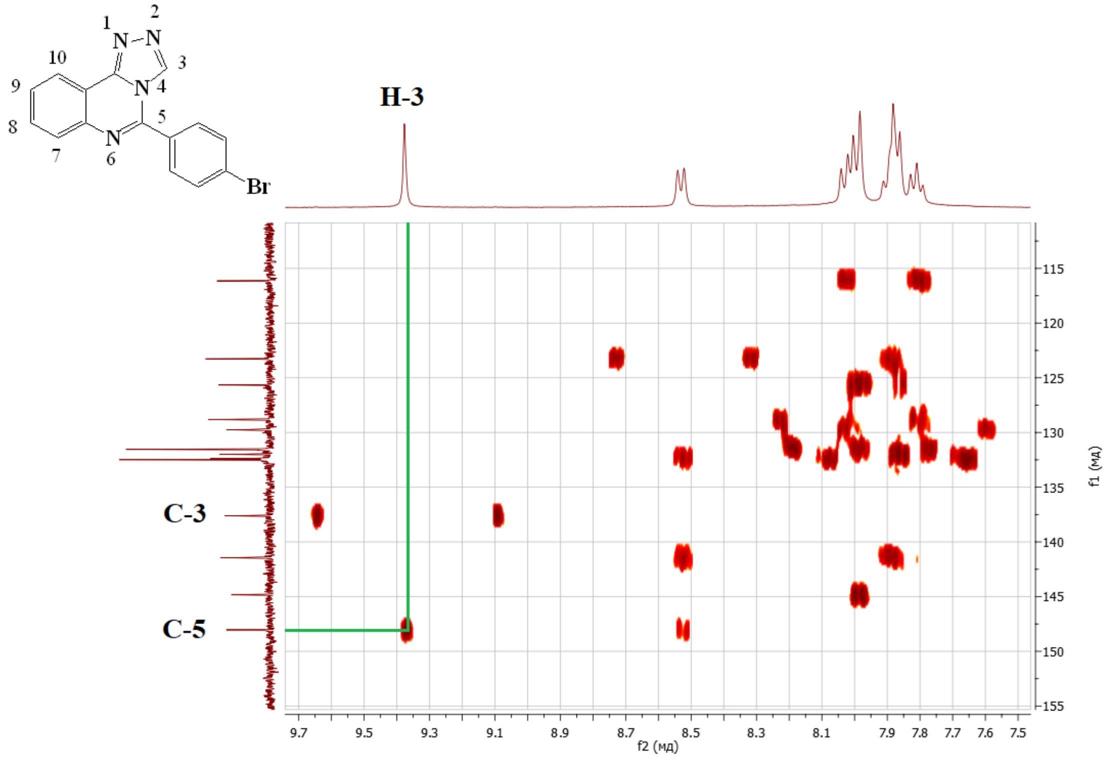

d

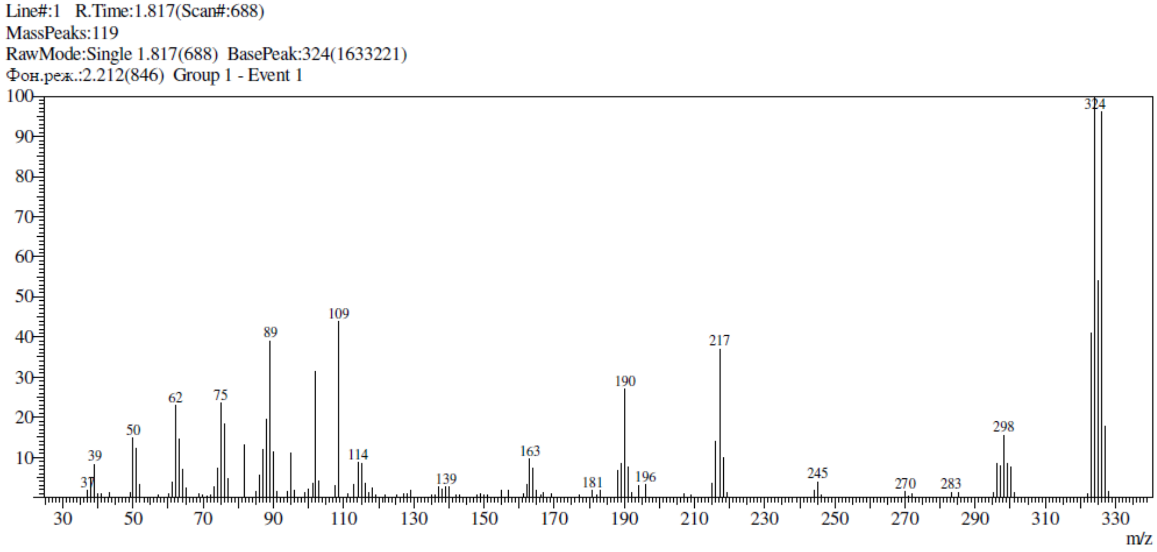

e

Figure S1. ^{1}H (a), ^{13}C (b) NMR spectra of **2a** in DMSO-d6; ^{1}H-^{1}H NOESY (c) and ^{1}H-^{13}C HMBC (d) spectra of **2a** in DMSO-d6; mass spectrum (e) of **2a**.

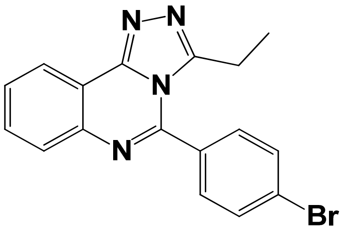
N
N
N
N
Br

2b

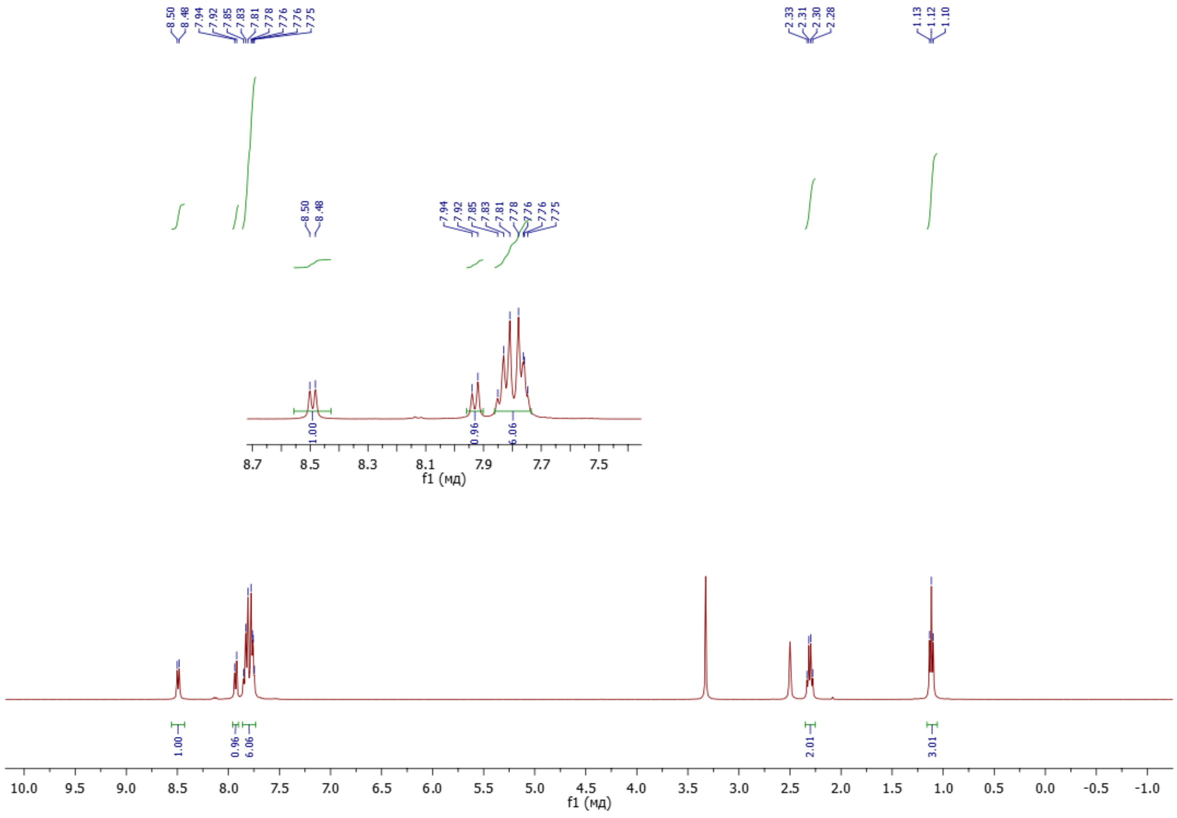
8.50
8.48
7.94
7.92
7.85
7.83
7.81
7.78
7.76
7.76
7.75
2.33
2.31
2.30
2.28
1.13
1.12
1.10
1.00
0.96
6.06
2.01
3.01
8.7 8.5 8.3 8.1 7.9 7.7 7.5
10.0 9.5 9.0 8.5 8.0 7.5 7.0 6.5 6.0 5.5 5.0 4.5 4.0 3.5 3.0 2.5 2.0 1.5 1.0 0.5 0.0 -0.5 -1.0
f1 (мд)

a

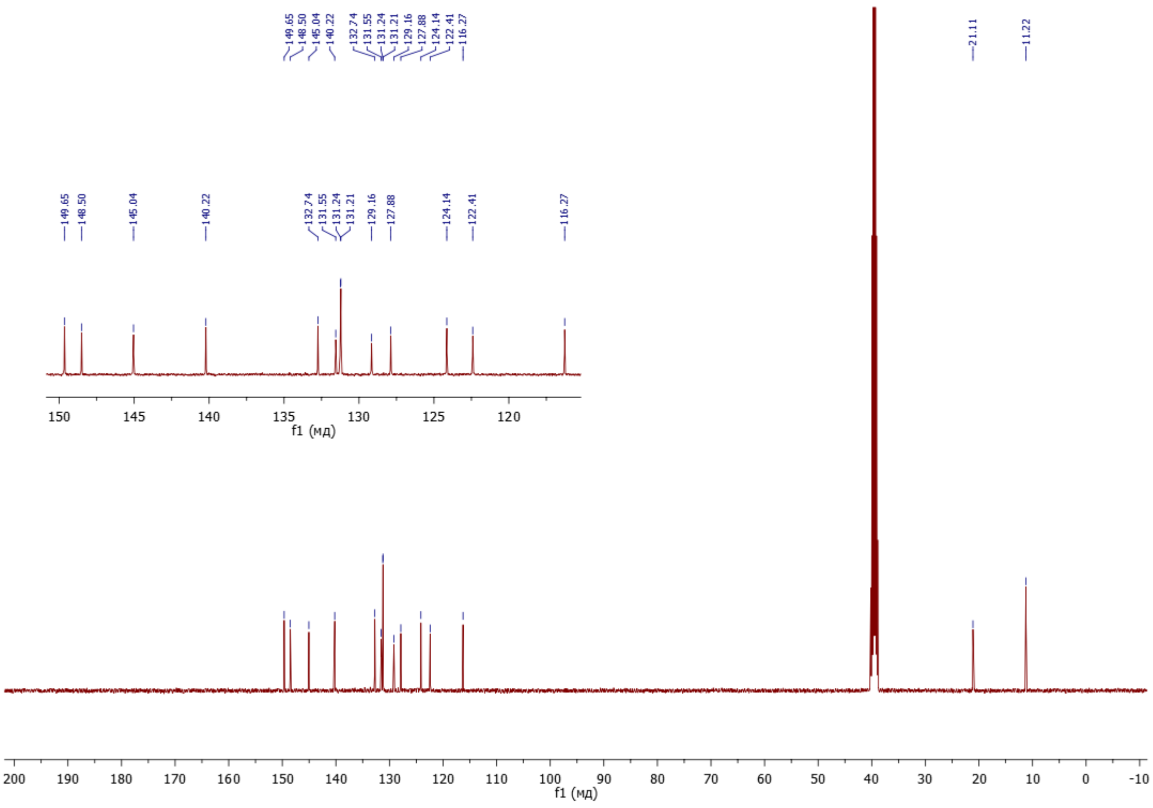

b

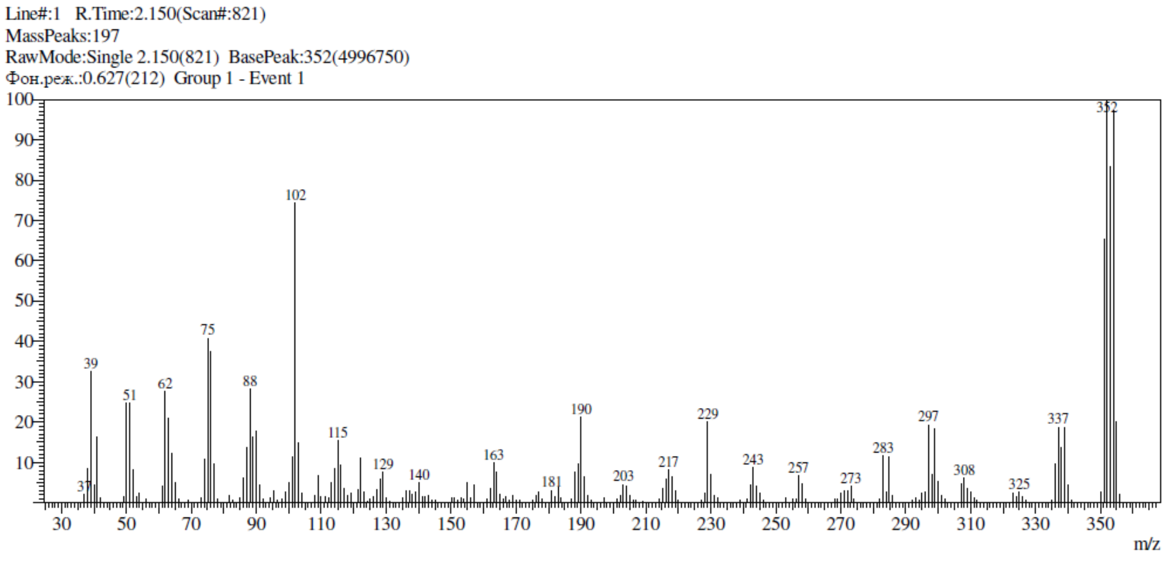

d

Figure S2. ^{1}H (a), ^{13}C (b) NMR spectra of **2b** in DMSO-d_6; mass spectrum (c) of **2b.**

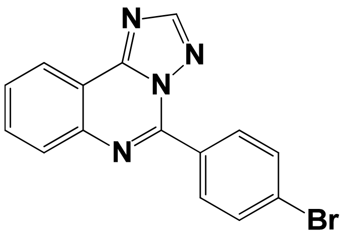

3a

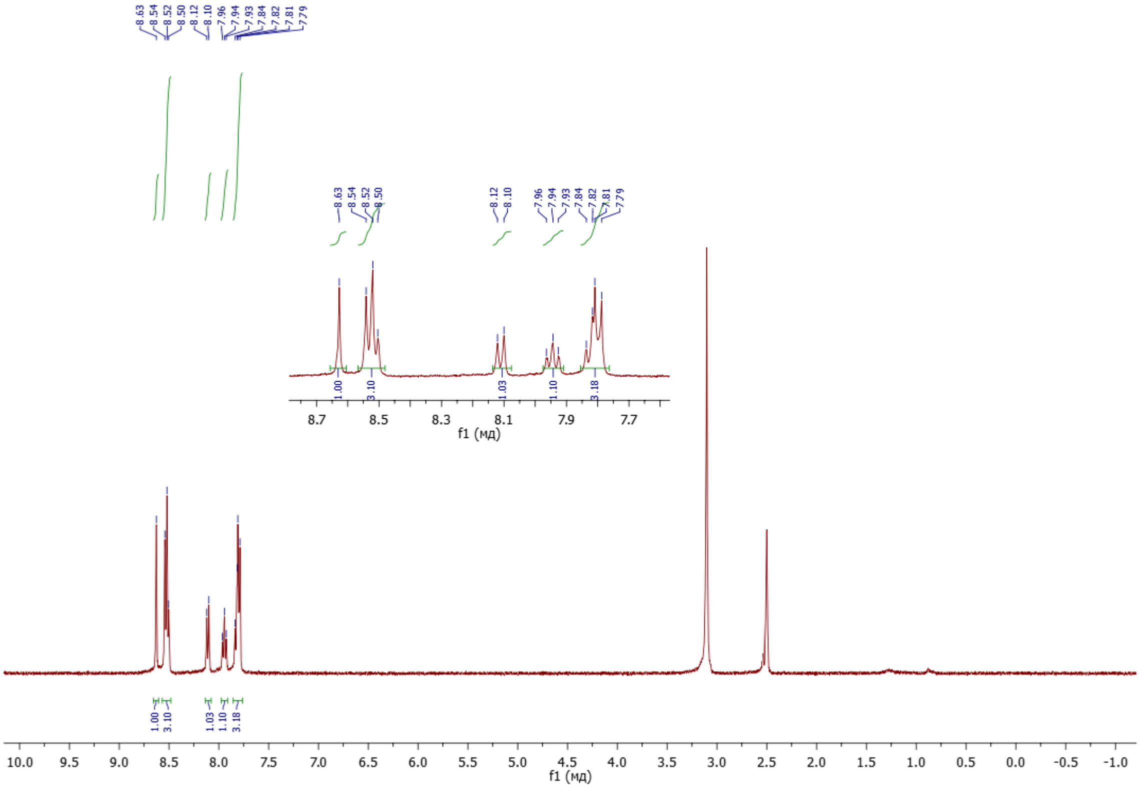

8.63
8.54
8.52
8.50
8.12
8.10
7.96
7.94
7.93
7.84
7.82
7.81
7.79
1.00
3.10
1.03
1.10
3.18
f1 (мд)

a

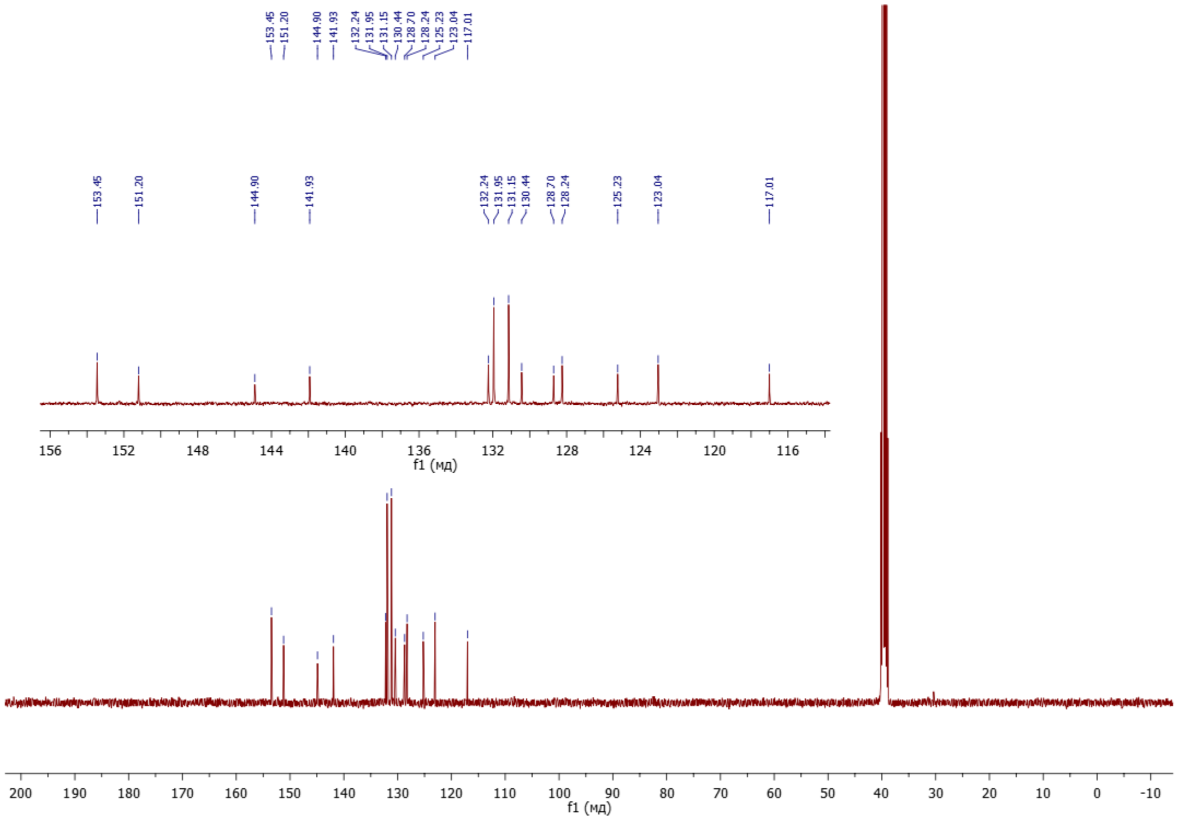
153.45
151.20
144.90
141.93
132.24
131.95
131.15
130.44
128.70
128.24
125.23
123.04
117.01
f1 (мд)

b

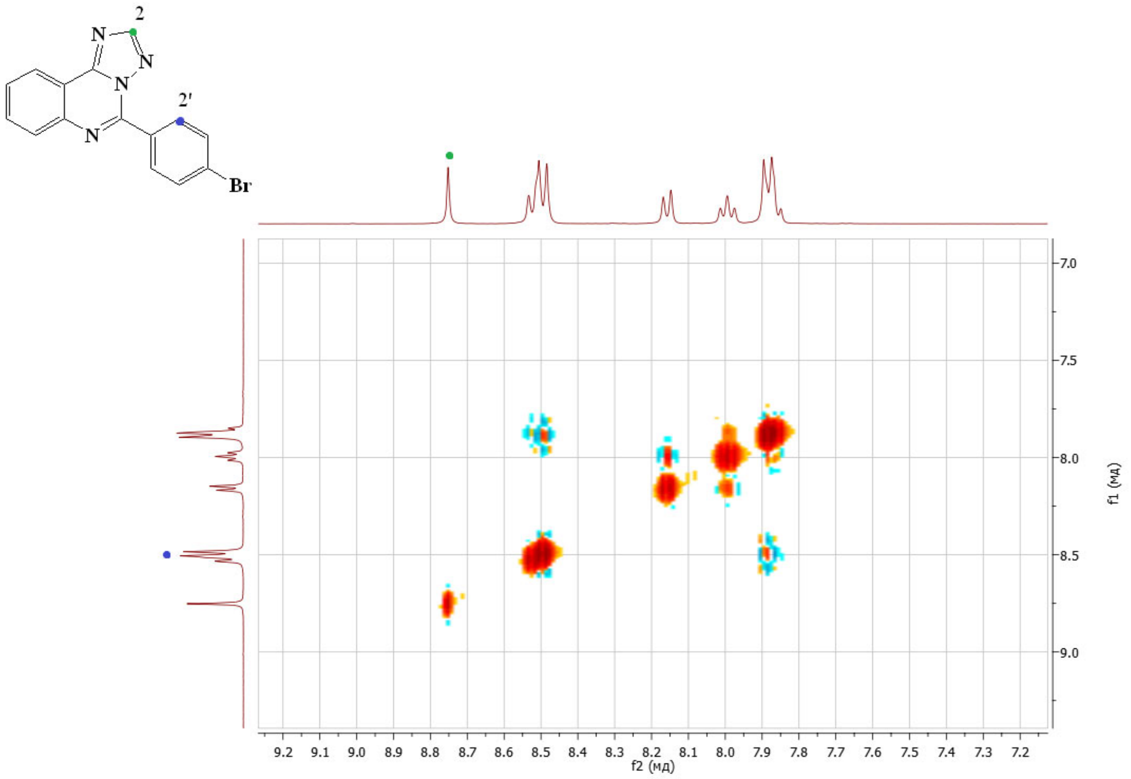
2
N
N
N
N
2′
Br
f2 (мд)
f1 (мд)

c

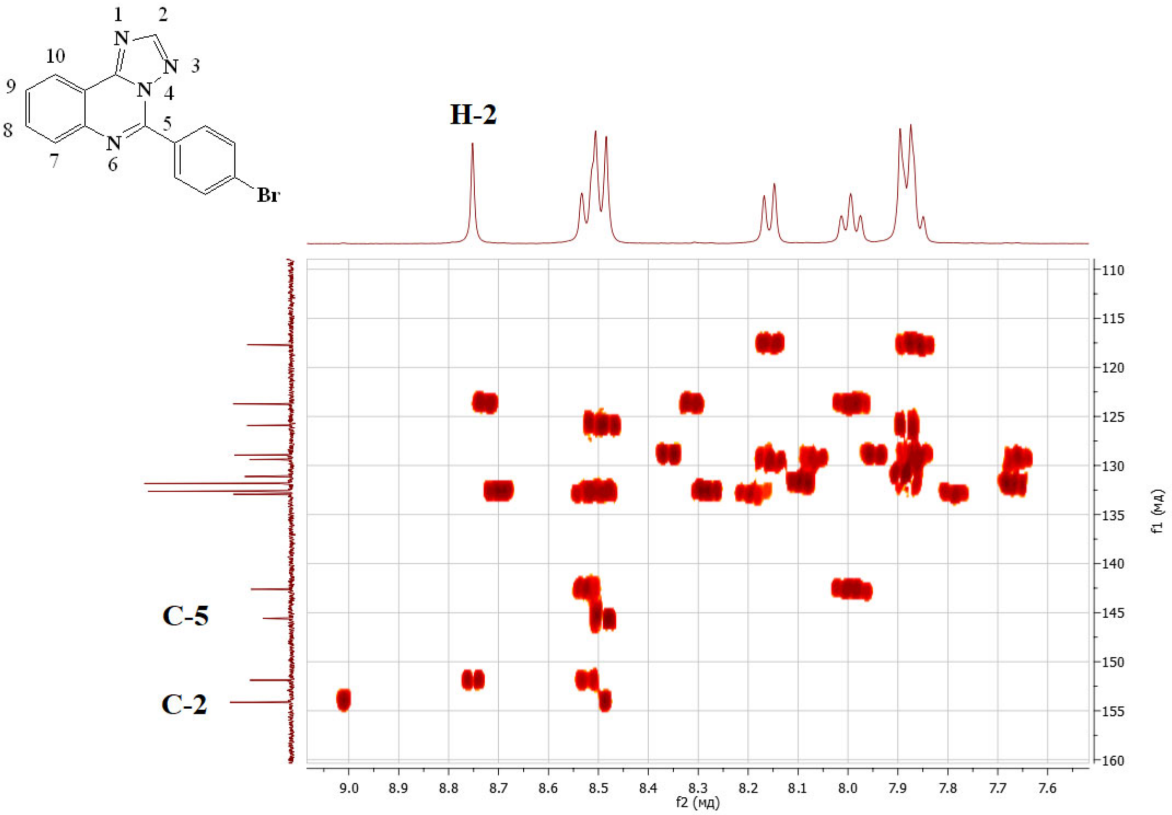

d

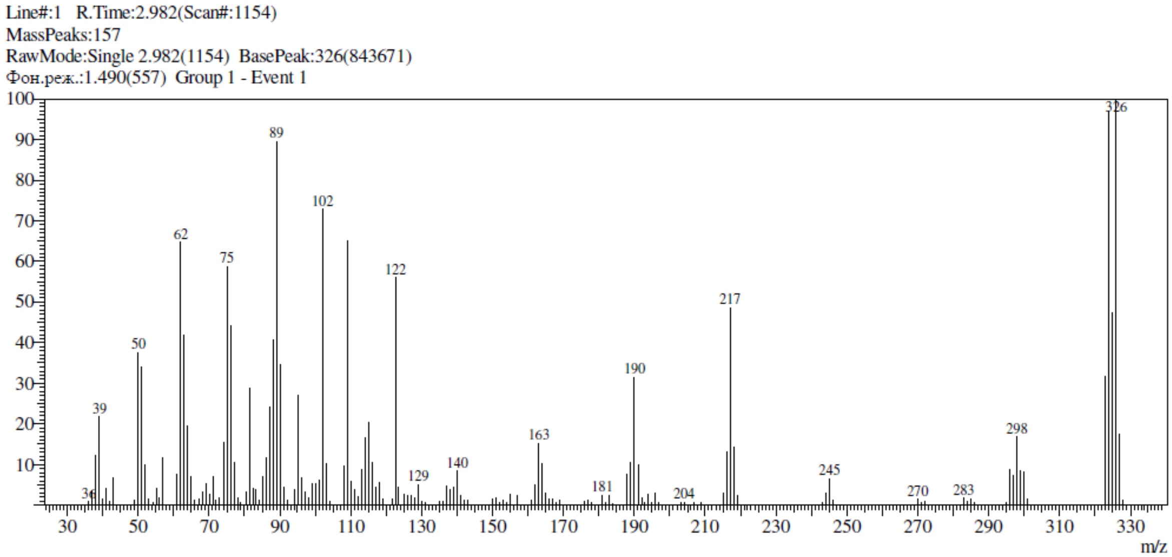

d

Figure S3. ^{1}H (a), ^{13}C (b) NMR spectra of **3a** in DMSO-d_6; and ^{1}H-^{1}H NOESY (c) ^{1}H-^{13}C HMBC (d) spectra of **3a** in DMSO-d_6; mass spectrum (d) of **3a**.

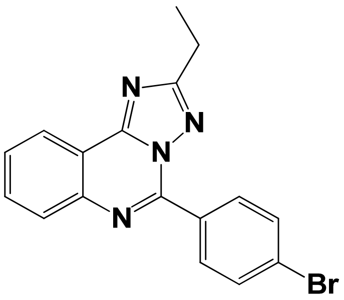
N
N
N
N
Br

3b

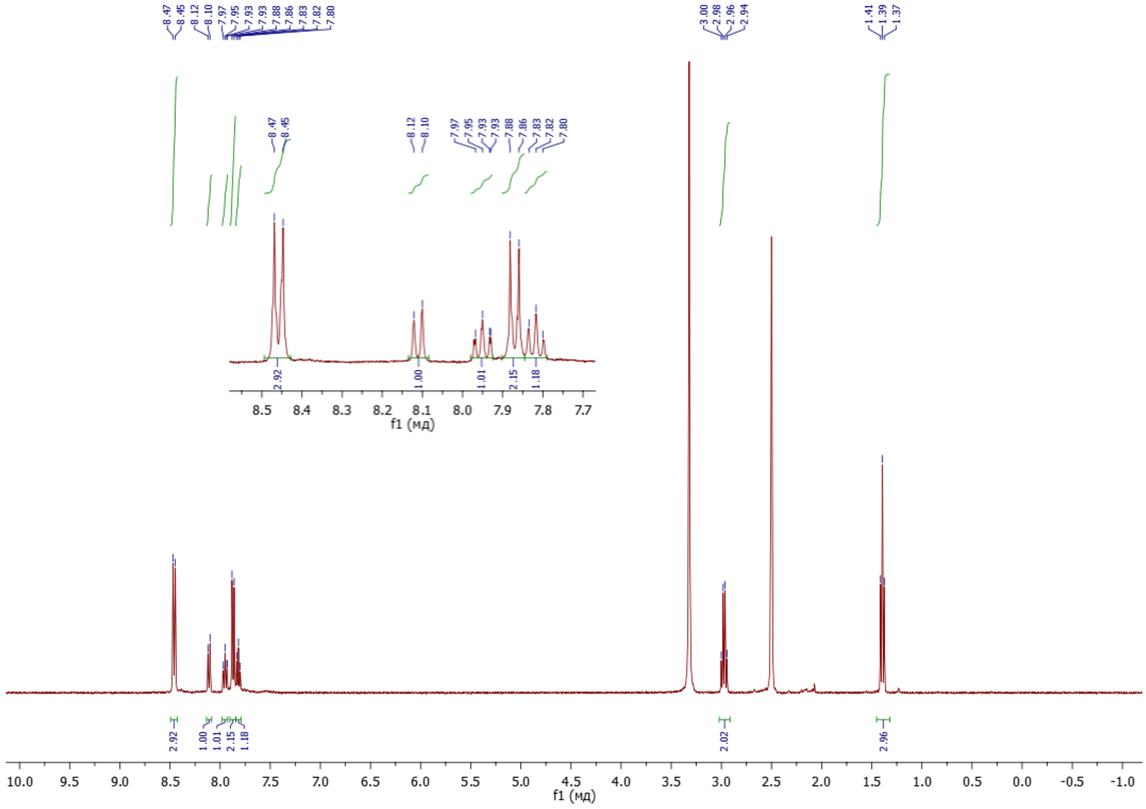
8.47
8.45
8.12
8.10
7.97
7.95
7.93
7.93
7.88
7.86
7.83
7.82
7.80
3.00
2.98
2.96
2.94
1.41
1.39
1.37
2.92
1.00
1.01
2.15
1.18
2.02
2.96
f1 (мд)

a

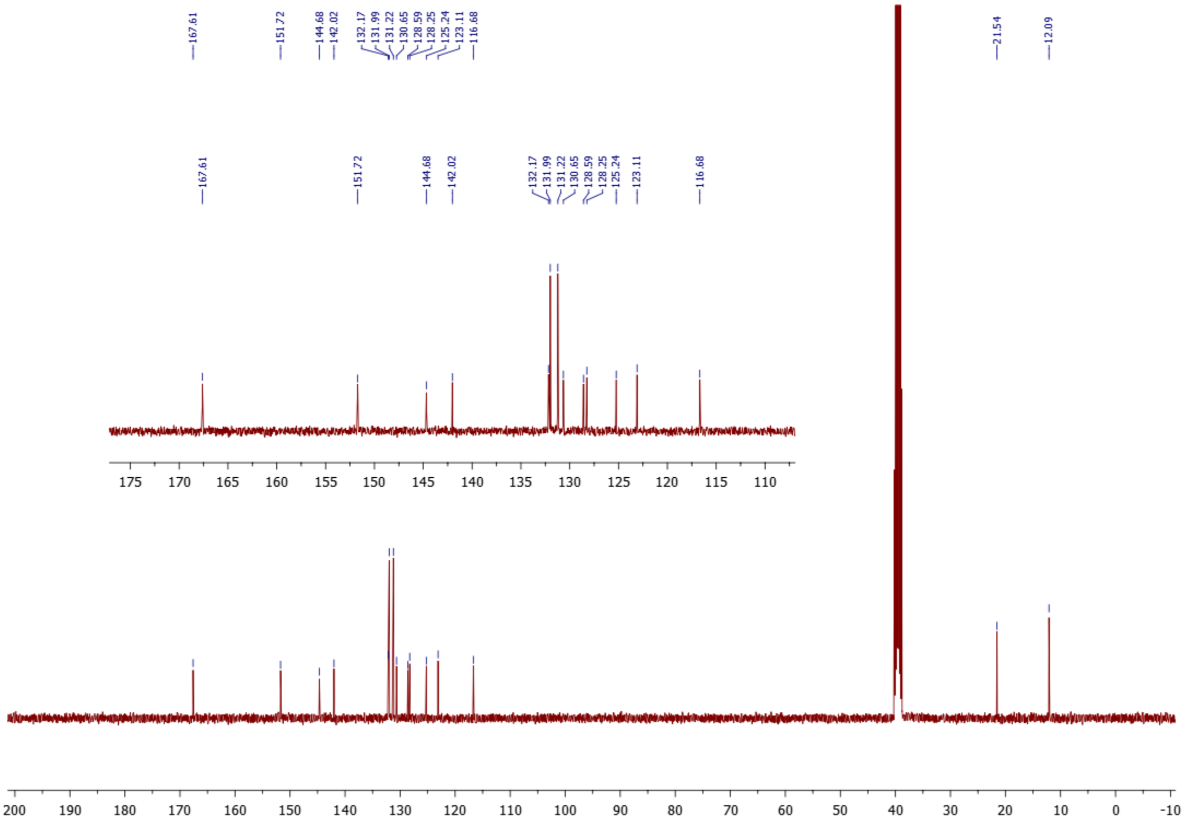

b

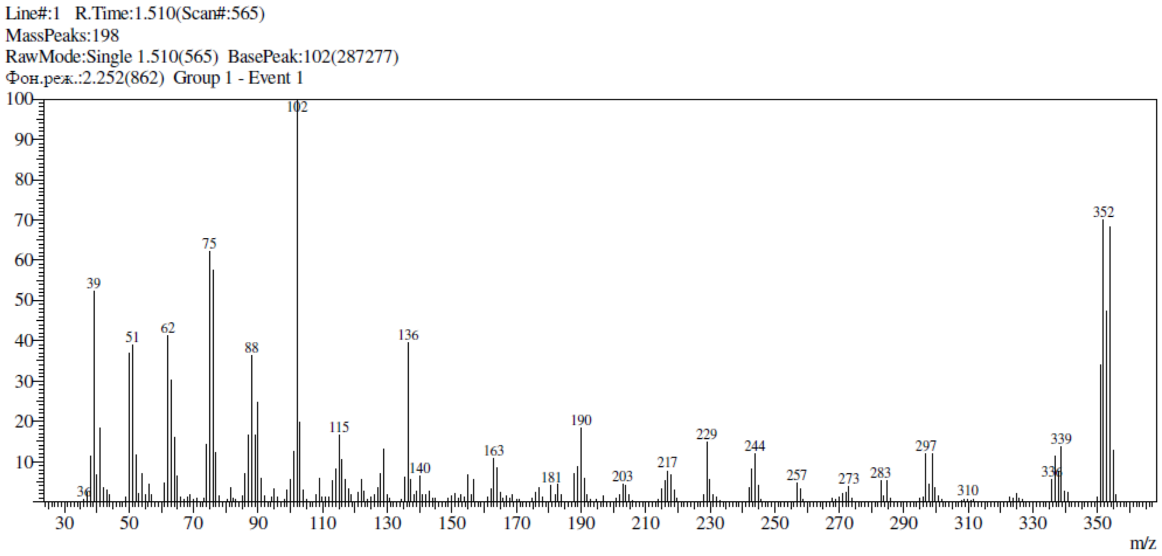

c

Figure S4. ^{1}H (a), ^{13}C (b) NMR spectra of **3b** in DMSO-d_6; mass spectrum (c) of **3b.**

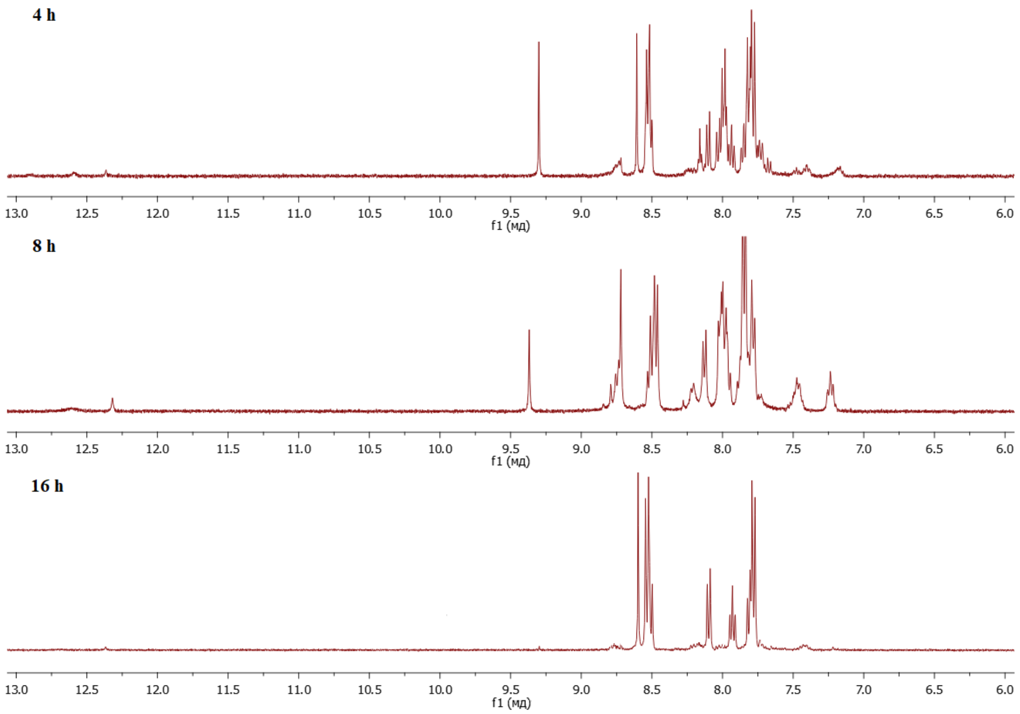

a

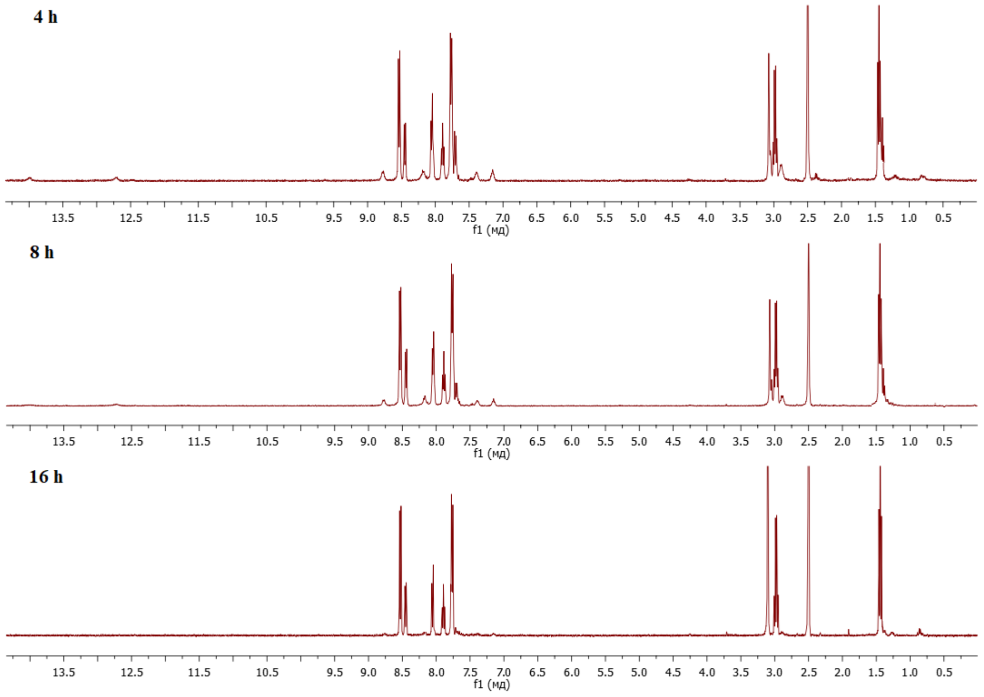

b

Figure S5. ^{1}H NMR monitoring of the reaction progress of 2-(4-bromophenyl)-4-hydrazinoquinazoline with triethyl orthoformate (a) or triethyl orthopropionate (b) in acetic media after 4 h, 8 h and 16 h. Spectra were recorded in DMSO-d_6.

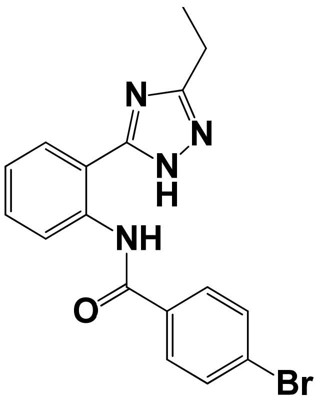
N
N
N
H
NH
O
Br

3b’

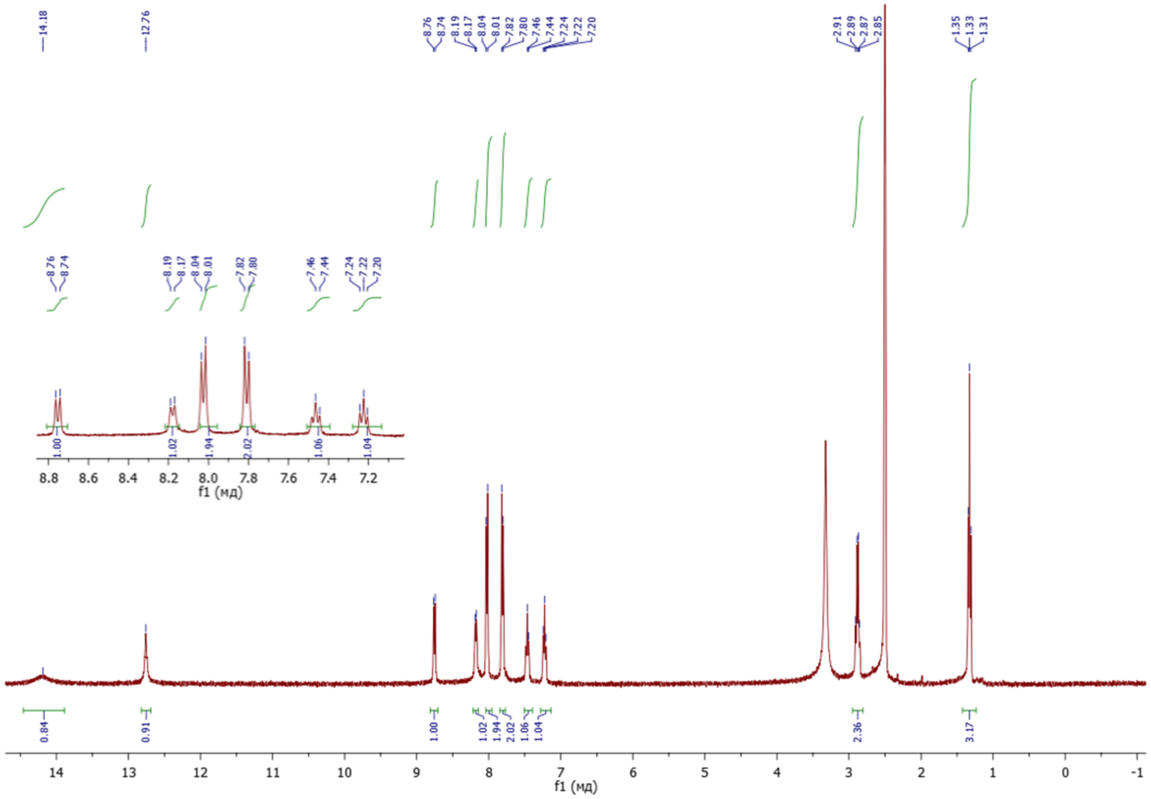
14.18
12.76
8.76
8.74
8.19
8.17
8.04
8.01
7.82
7.80
7.46
7.44
7.24
7.22
7.20
2.91
2.89
2.87
2.85
1.35
1.33
1.31
1.00
1.02
1.94
2.02
1.06
1.04
8.8
8.6
8.4
8.2
8.0
7.8
7.6
7.4
7.2
f1 (мд)
0.84
0.91
1.00
1.02
1.94
2.02
1.06
1.04
2.36
3.17
14
13
12
11
10
9
8
7
6
5
4
3
2
1
0
-1
f1 (мд)

a

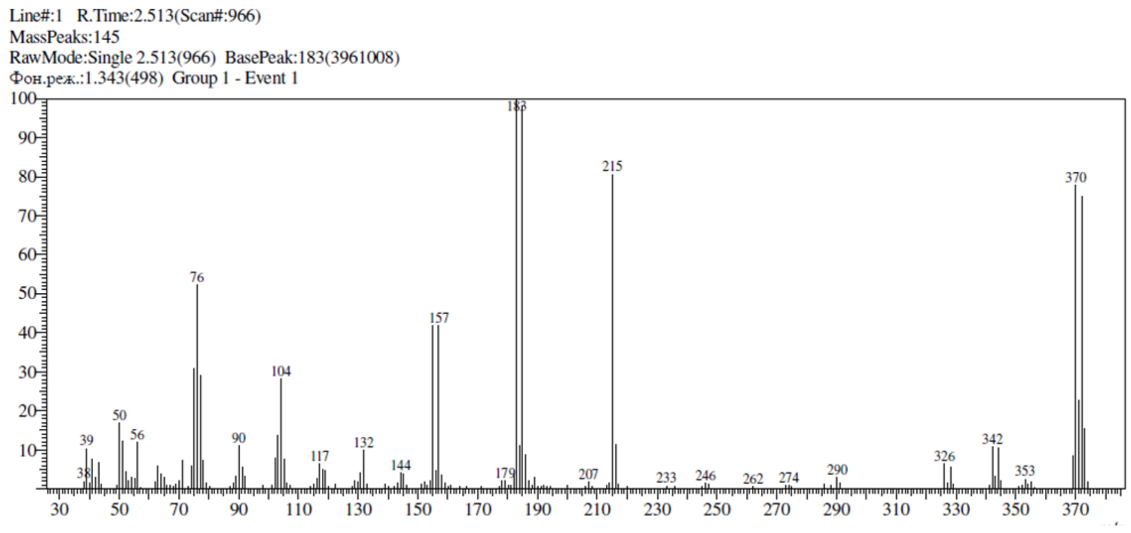

b

Figure S6. ^{1}H (a) NMR spectrum of **3b'** in DMSO-d6; mass spectrum (b) of **3b'**.

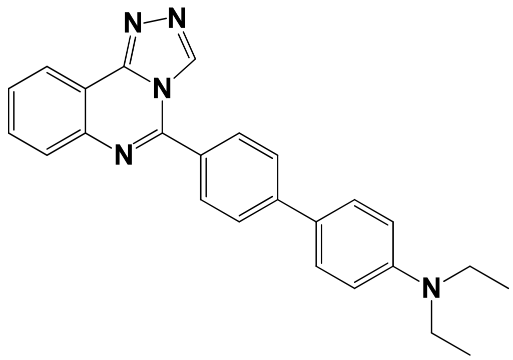

4a

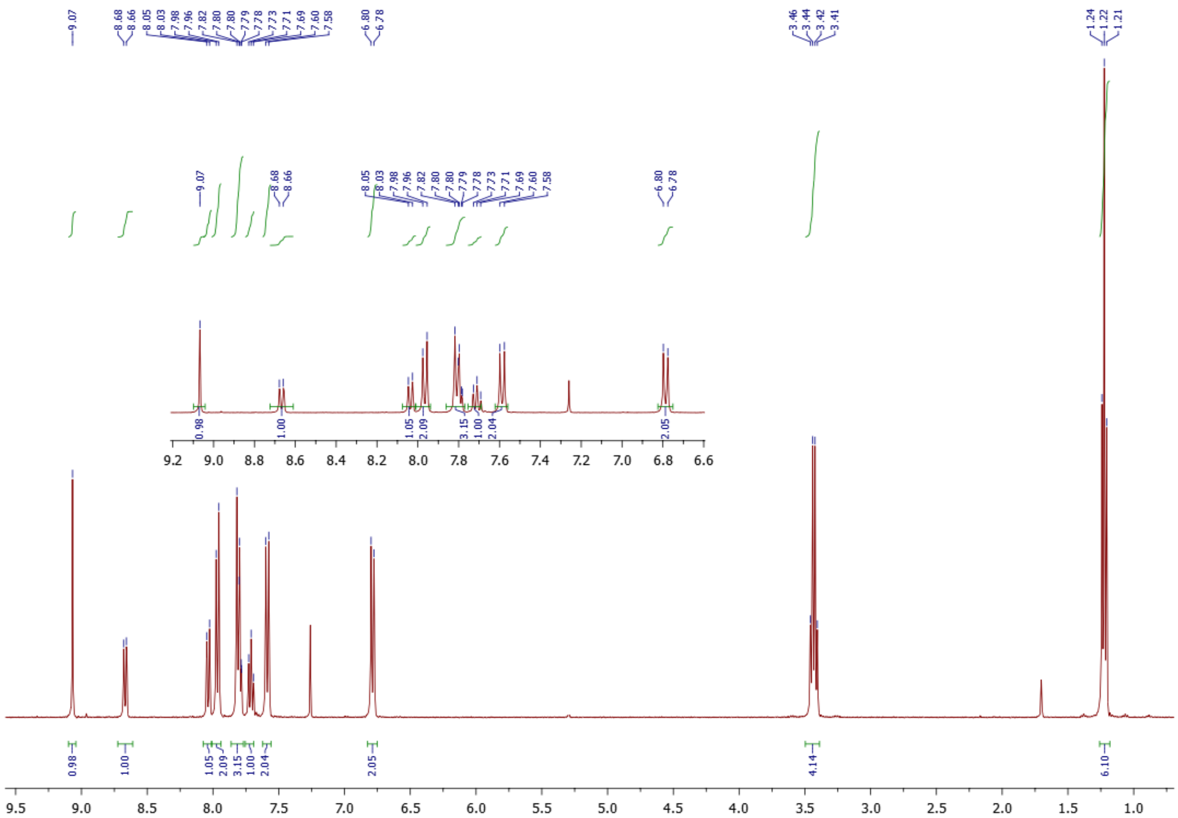

a

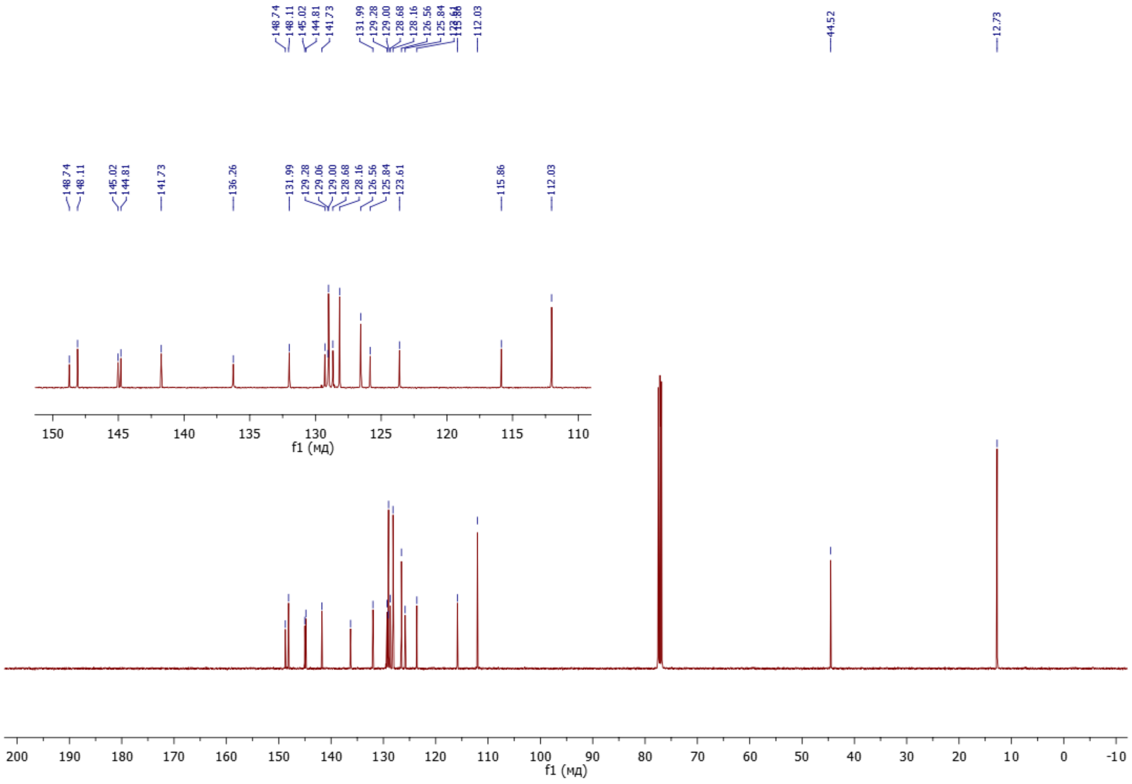

b

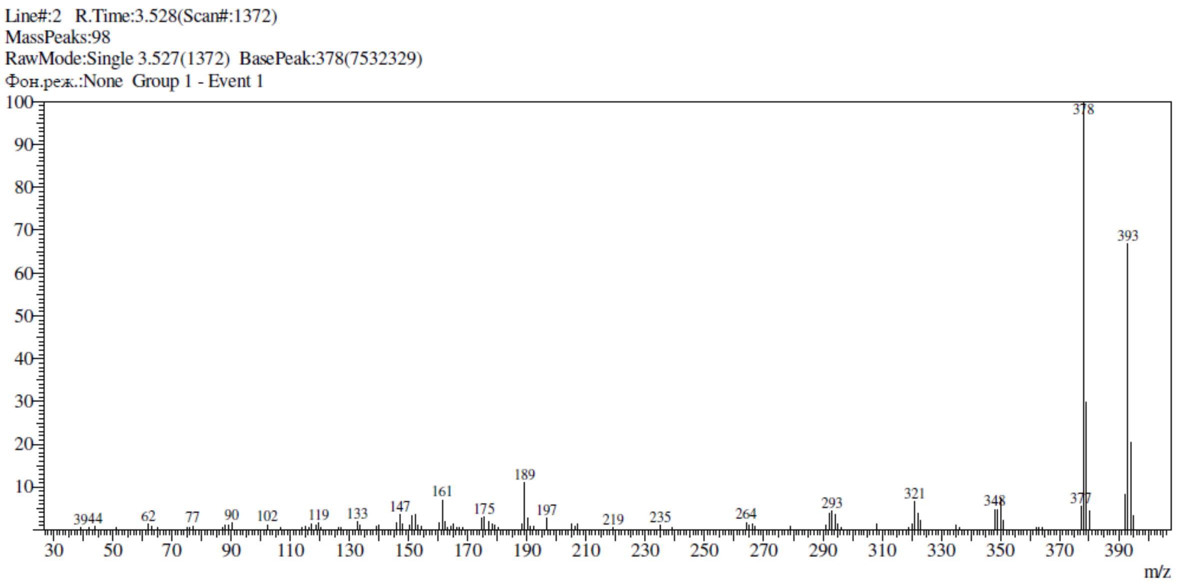

c

Figure S7. ^{1}H (a), ^{13}C (b) NMR spectra of **4a** in $DCCl_3$; mass spectrum (c) of **4a**.

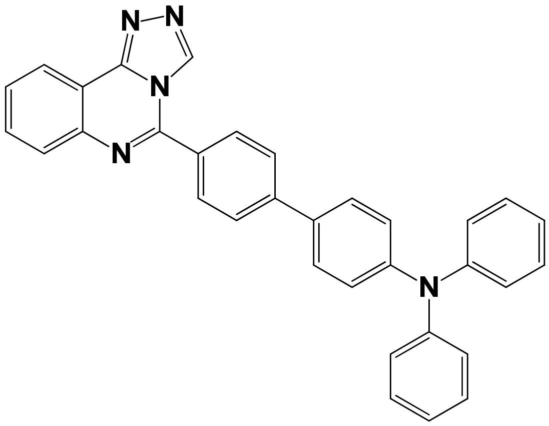

4b

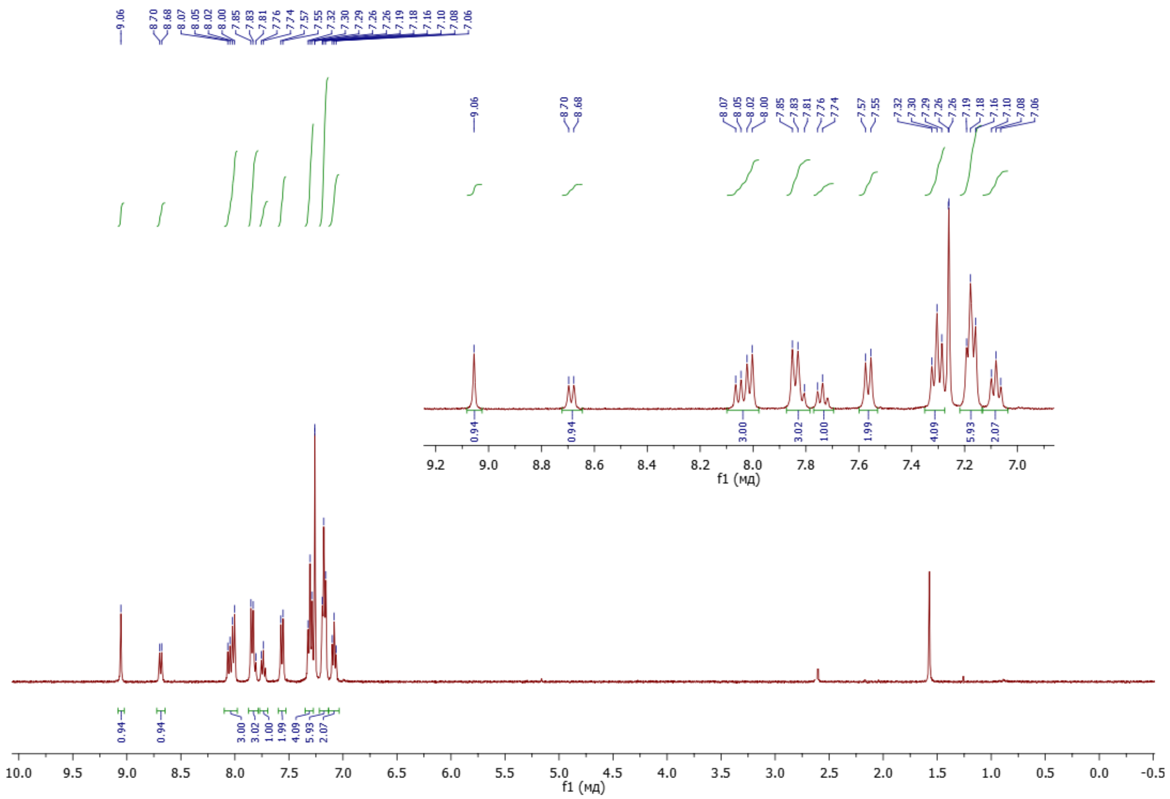
9.06
8.70
8.68
8.07
8.05
8.02
8.00
7.85
7.83
7.81
7.76
7.74
7.57
7.55
7.32
7.30
7.29
7.26
7.26
7.19
7.18
7.16
7.10
7.08
7.06
0.94
0.94
3.00
3.02
1.00
1.99
4.09
5.93
2.07
f1 (мд)

a

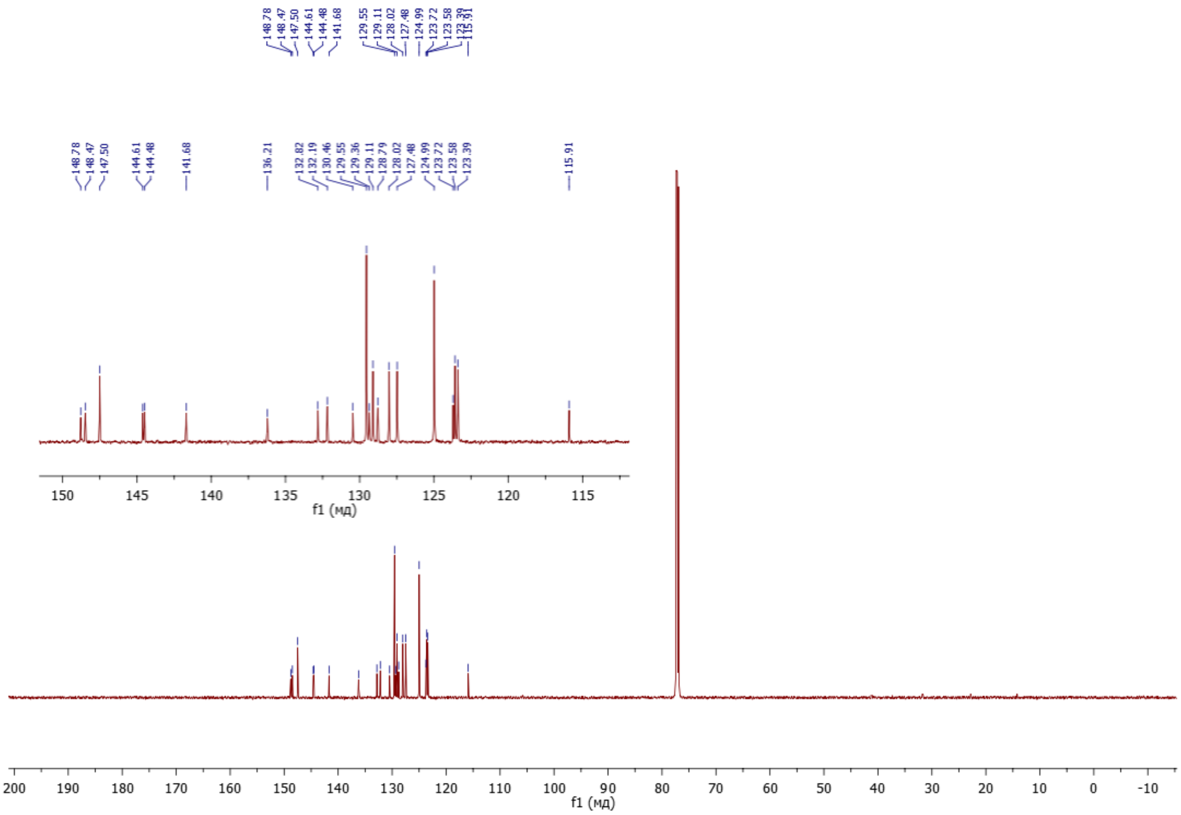
148.78
148.47
147.50
144.61
144.48
141.68
136.21
132.82
132.19
130.46
129.55
129.36
129.11
128.79
128.02
127.48
124.99
123.72
123.58
123.39
115.91
f1 (мд)

b

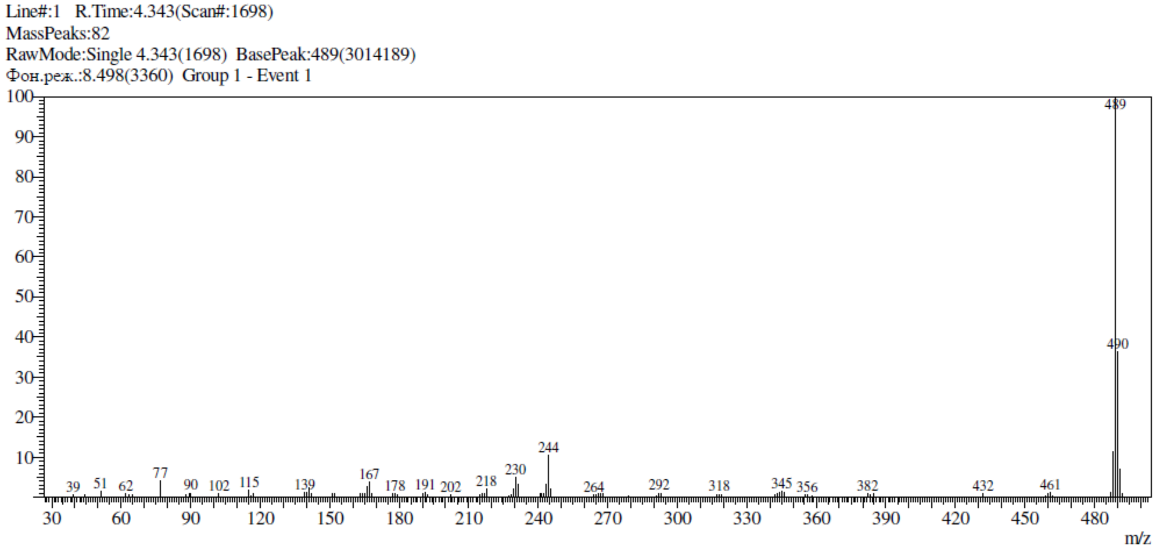

c

Figure S8. ^{1}H (a), ^{13}C (b) NMR spectra of **4b** in $DCCl_3$; mass spectrum (c) of **4b.**

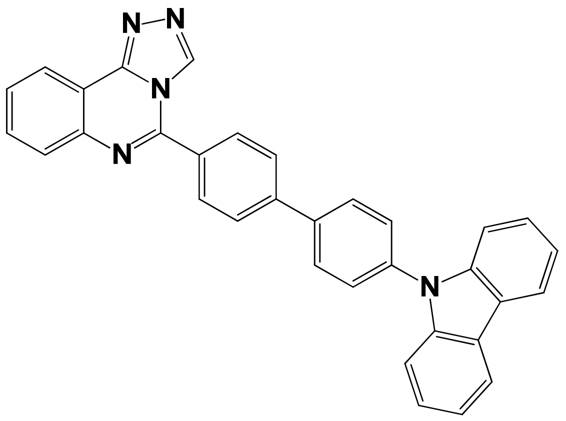

4c

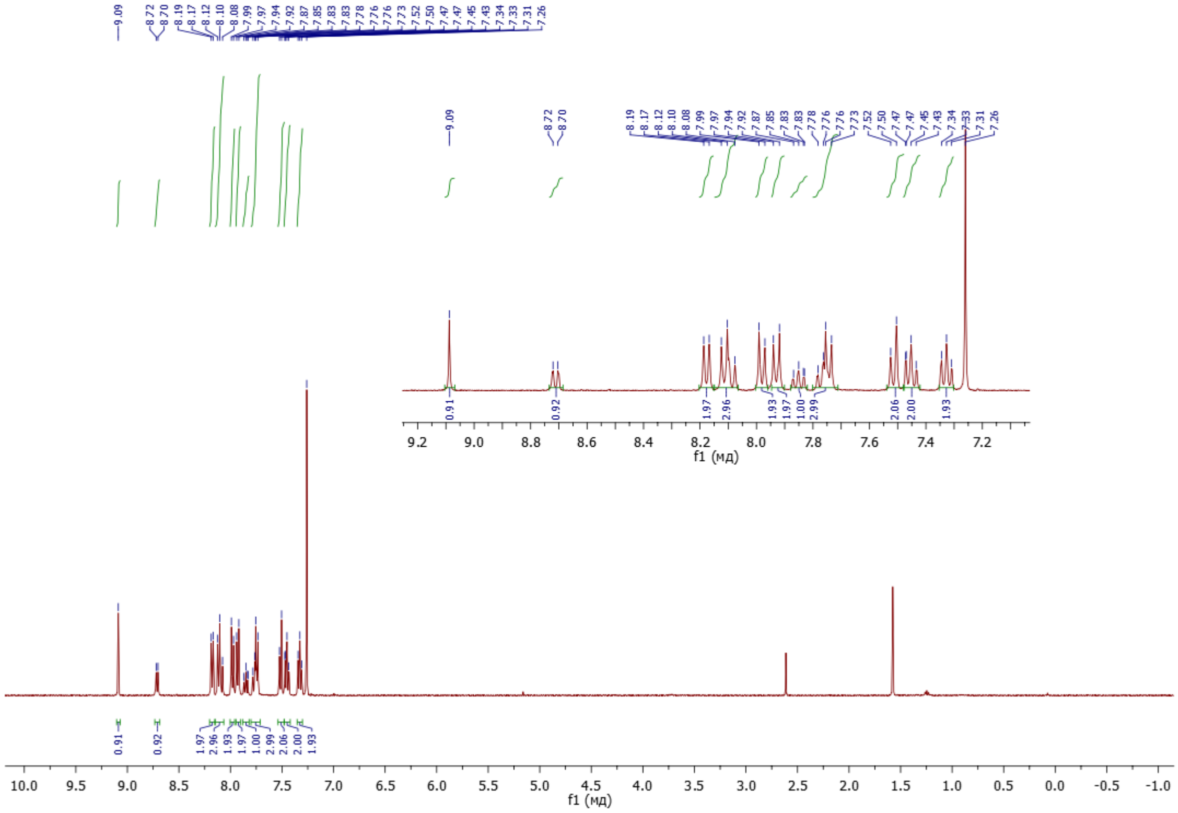
9.09 8.72 8.70 8.19 8.17 8.12 8.10 8.08 7.99 7.97 7.94 7.92 7.87 7.85 7.83 7.83 7.78 7.76 7.76 7.73 7.52 7.50 7.47 7.47 7.45 7.43 7.34 7.33 7.31 7.26
0.91 0.92 1.97 2.96 1.93 1.97 1.00 2.99 2.06 2.00 1.93
f1 (мд)

a

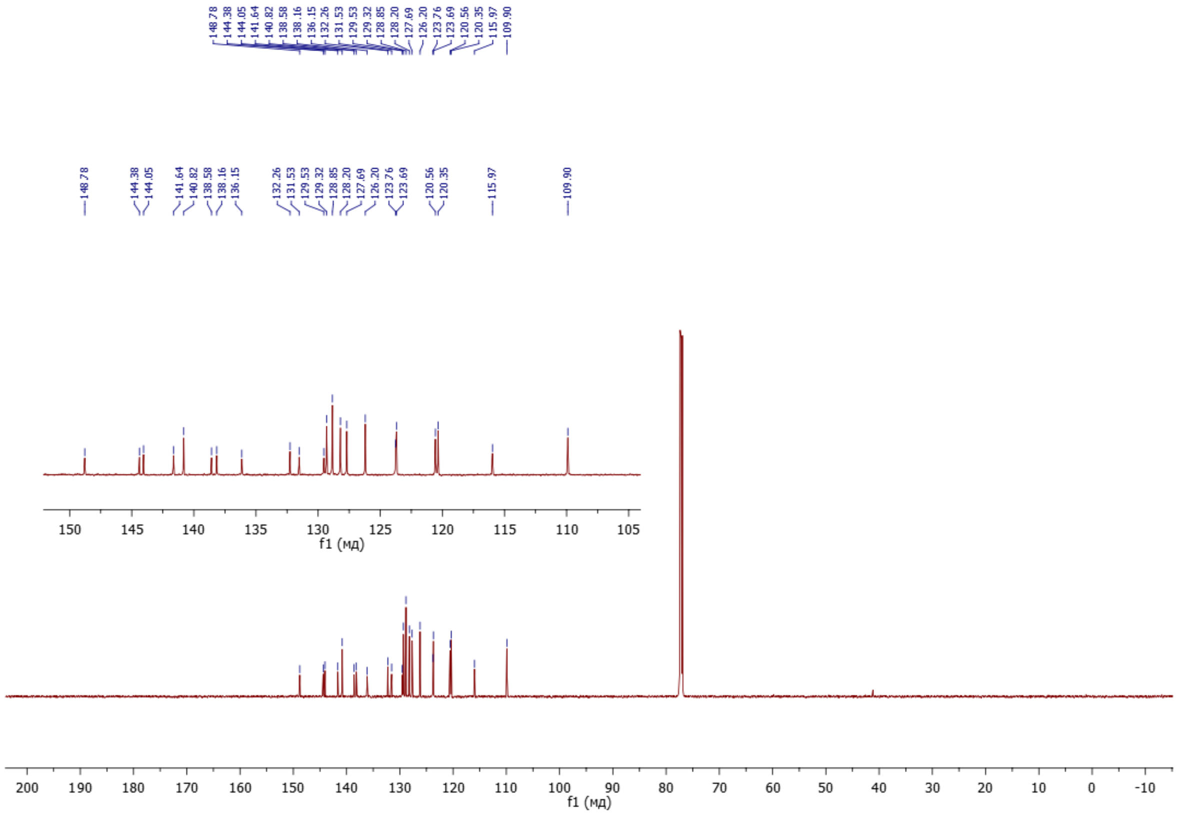
148.78 144.38 144.05 141.64 140.82 138.58 138.16 136.15 132.26 131.53 129.53 129.32 128.85 128.20 127.69 126.20 123.76 123.69 120.56 120.35 115.97 109.90
f1 (мд)

b

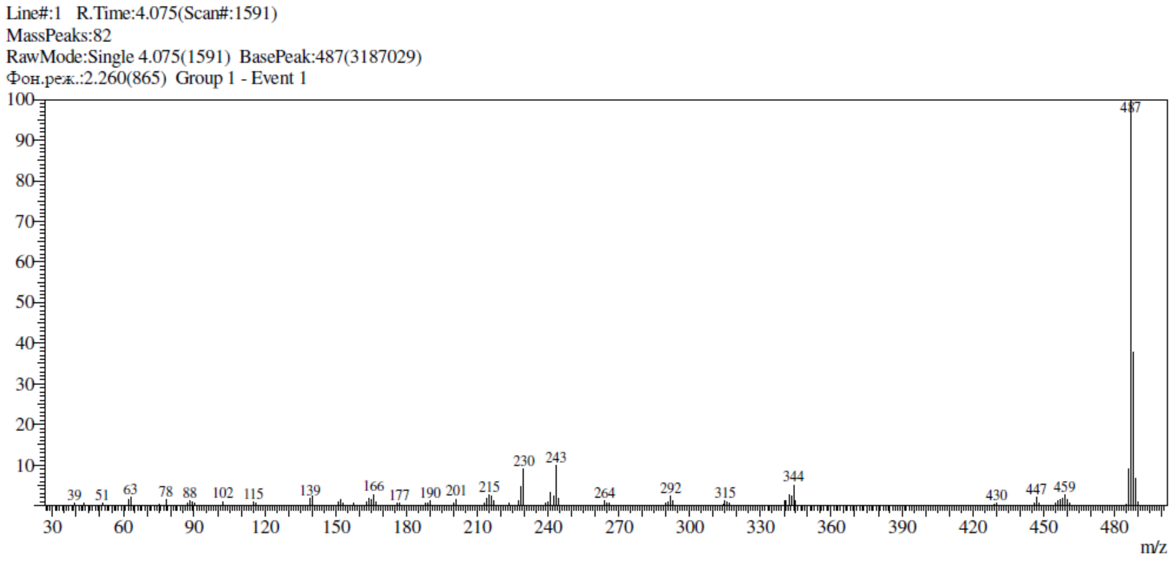

c

Figure S9. ^{1}H (a), ^{13}C (b) NMR spectra of **4c** in DCCl3; mass spectrum (c) of **4c.**

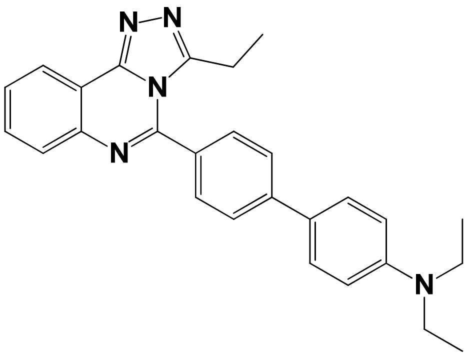

4d

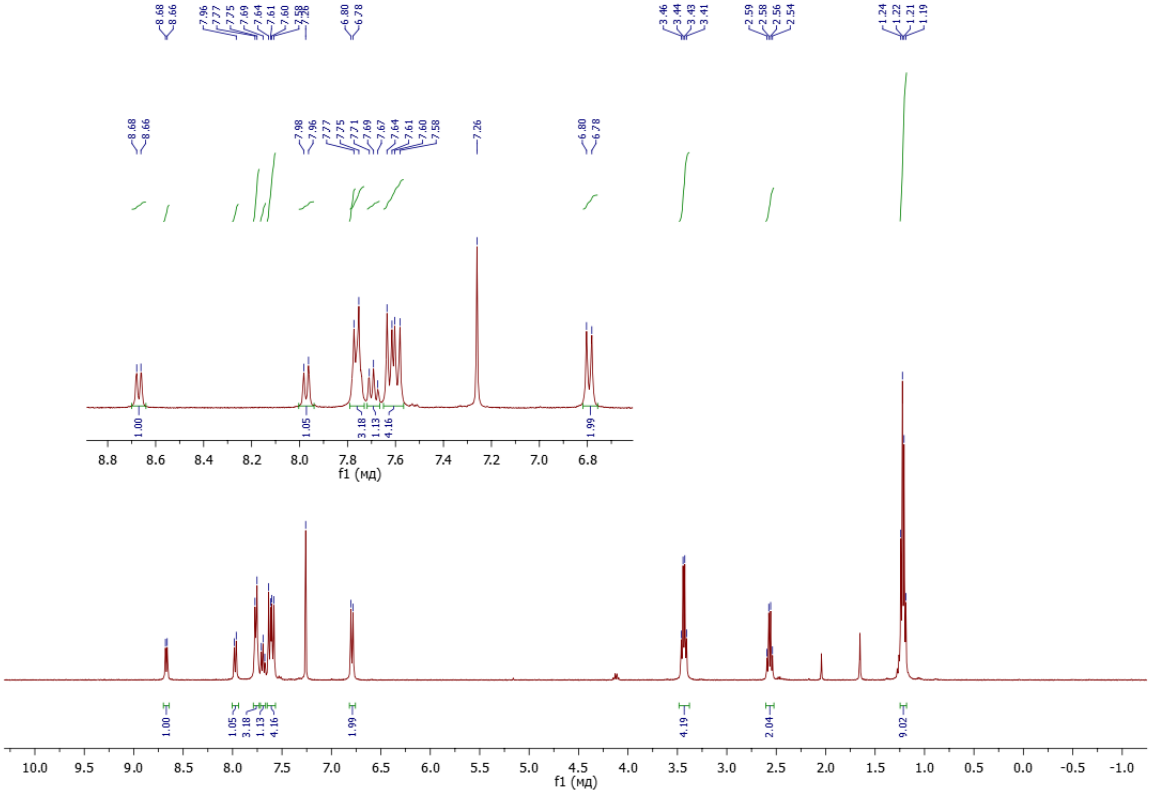
8.68
8.66
7.96
7.77
7.75
7.69
7.64
7.61
7.60
7.58
7.26
6.80
6.78
3.46
3.44
3.43
3.41
2.59
2.58
2.56
2.54
1.24
1.22
1.21
1.19
8.68
8.66
7.98
7.96
7.77
7.75
7.71
7.69
7.67
7.64
7.61
7.60
7.58
7.26
6.80
6.78
1.00
1.05
3.18
1.13
4.16
1.99
8.8
8.6
8.4
8.2
8.0
7.8
7.6
7.4
7.2
7.0
6.8
f1 (мд)
1.00
1.05
3.18
1.13
4.16
1.99
4.19
2.04
9.02
10.0
9.5
9.0
8.5
8.0
7.5
7.0
6.5
6.0
5.5
5.0
4.5
4.0
3.5
3.0
2.5
2.0
1.5
1.0
0.5
0.0
-0.5
-1.0
f1 (мд)

a

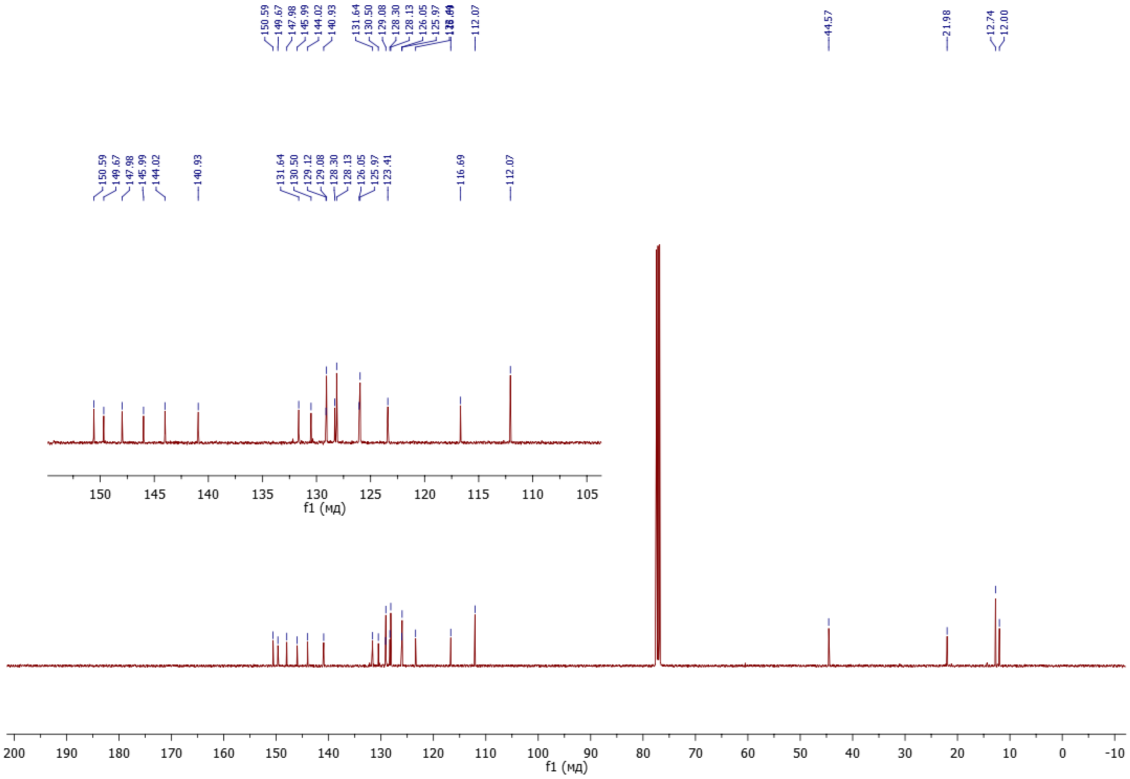
150.59
149.67
147.98
145.99
144.02
140.93
131.64
130.50
129.08
128.30
128.13
126.05
125.97
112.07
44.57
21.98
12.74
12.00
150.59
149.67
147.98
145.99
144.02
140.93
131.64
130.50
129.12
129.08
128.30
128.13
126.05
125.97
123.41
116.69
112.07
150
145
140
135
130
125
120
115
110
105
f1 (мд)
200
190
180
170
160
150
140
130
120
110
100
90
80
70
60
50
40
30
20
10
0
-10
f1 (мд)

b

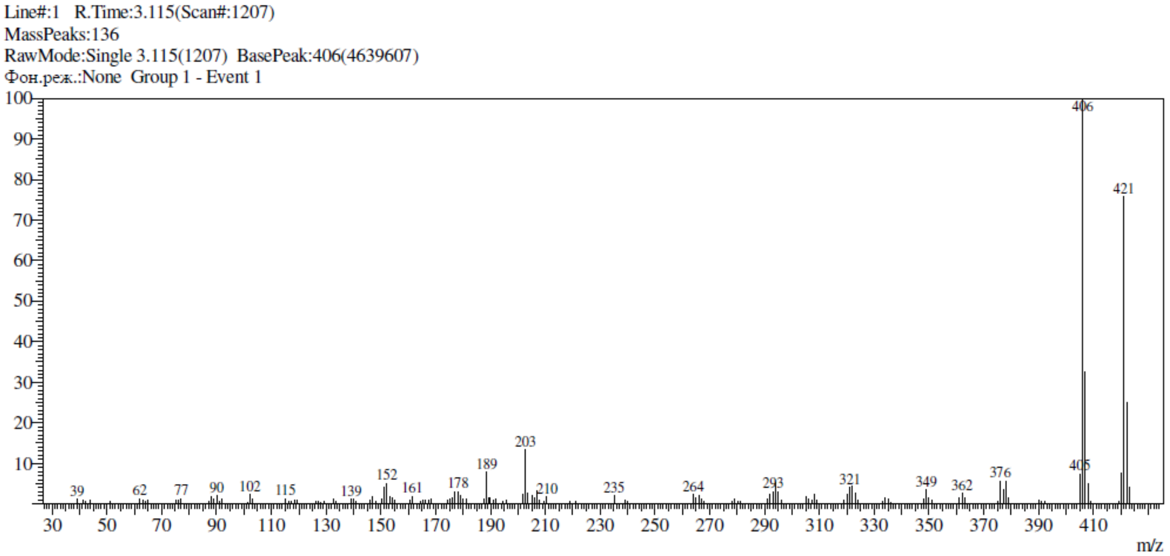

c

Figure S10. ^{1}H (a), ^{13}C (b) NMR spectra of **4d** in $DCCl_3$; mass spectrum (c) of **4d**.

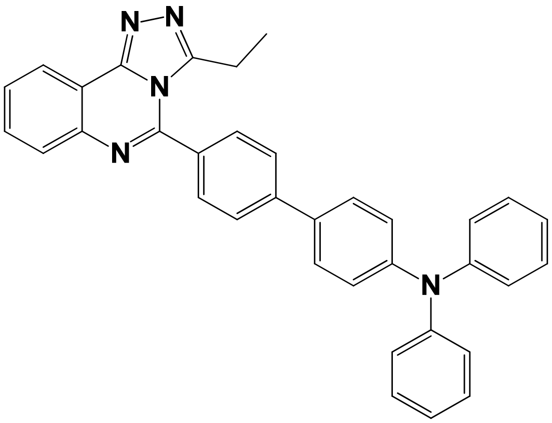

4e

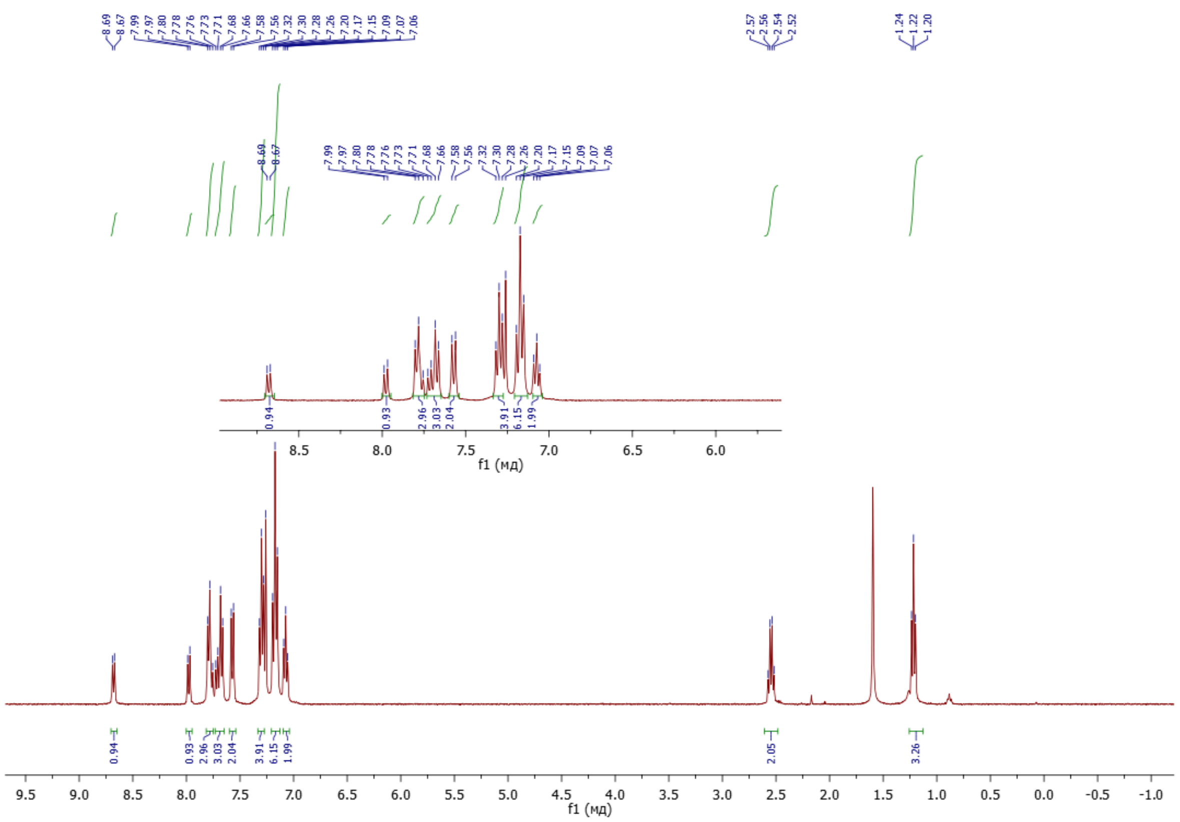

a

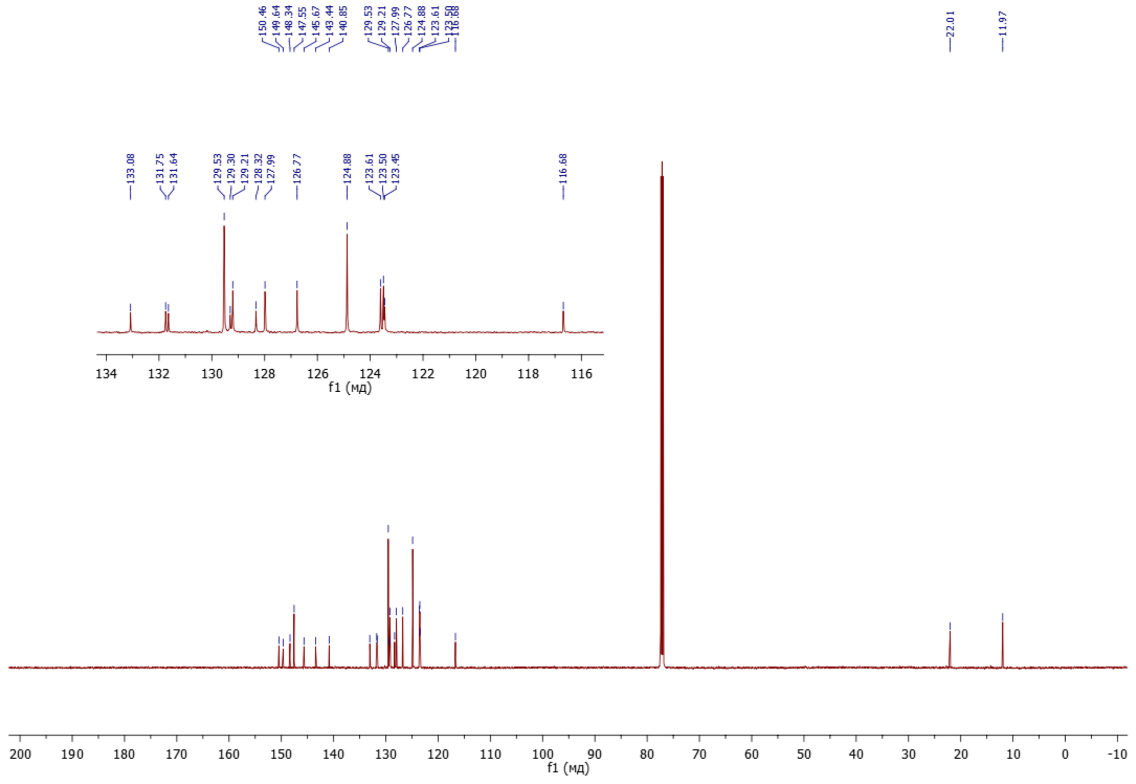

b

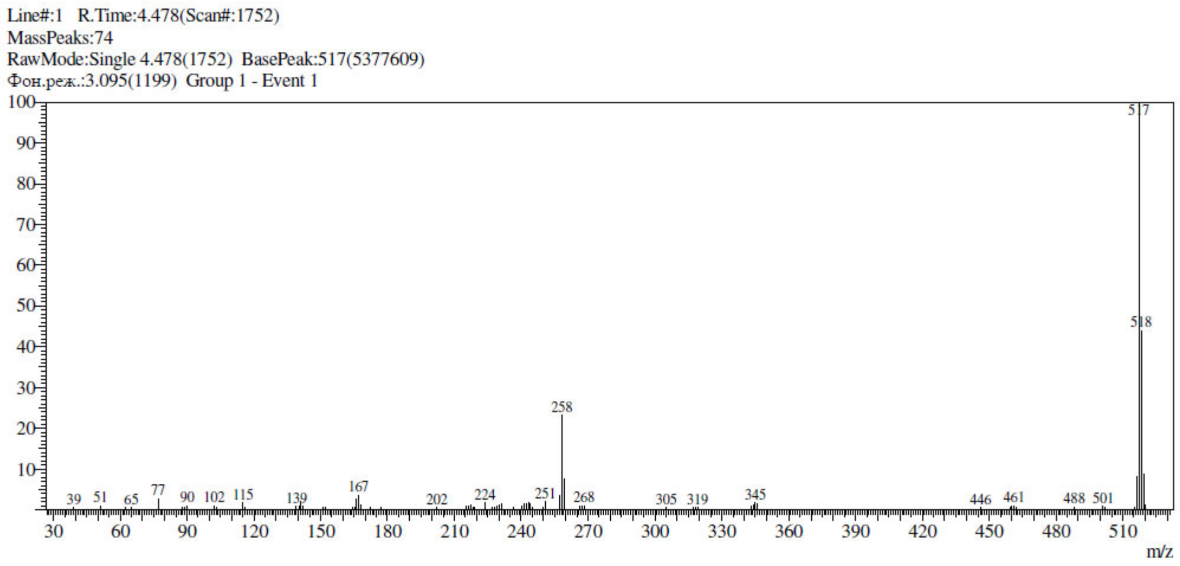

c

Figure S11. ^{1}H (a), ^{13}C (b) NMR spectra of **4e** in $DCCl_3$; mass spectrum (c) of **4e**.

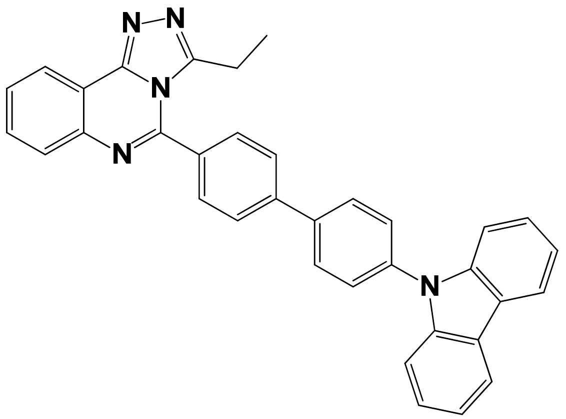

4f

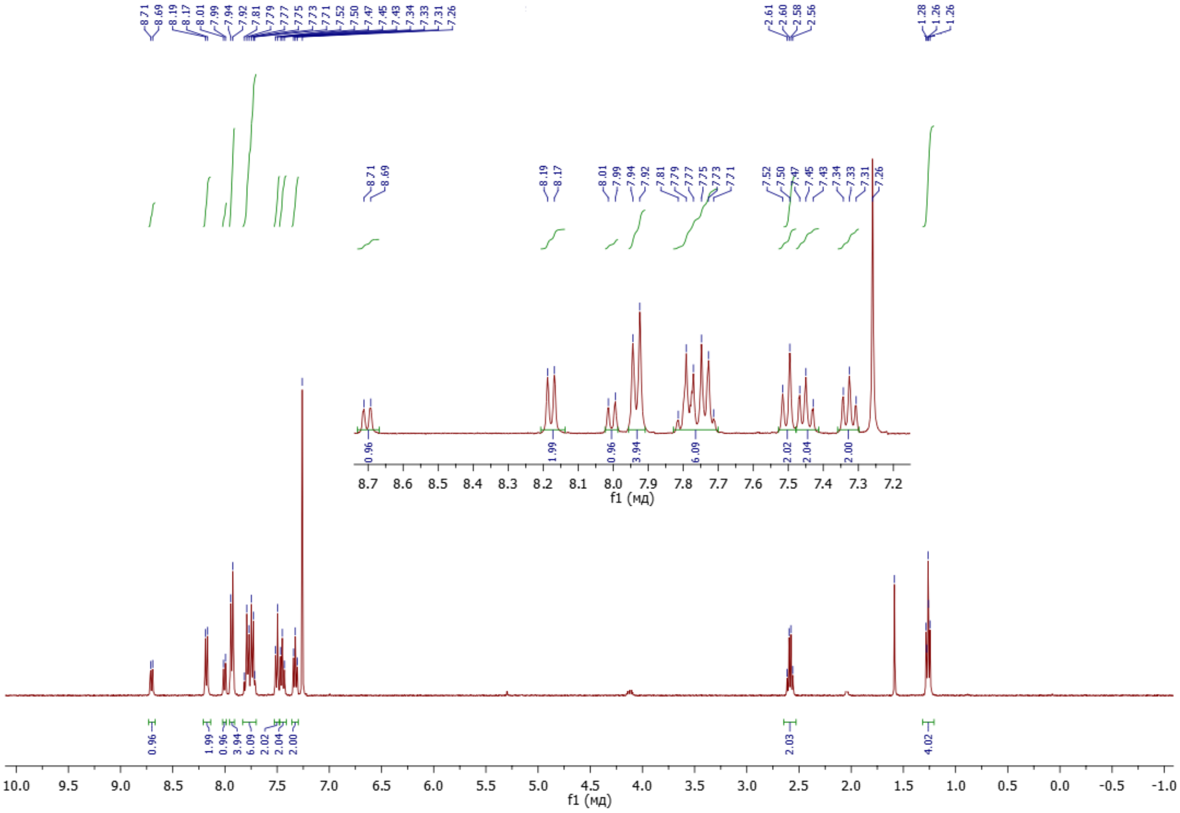

a

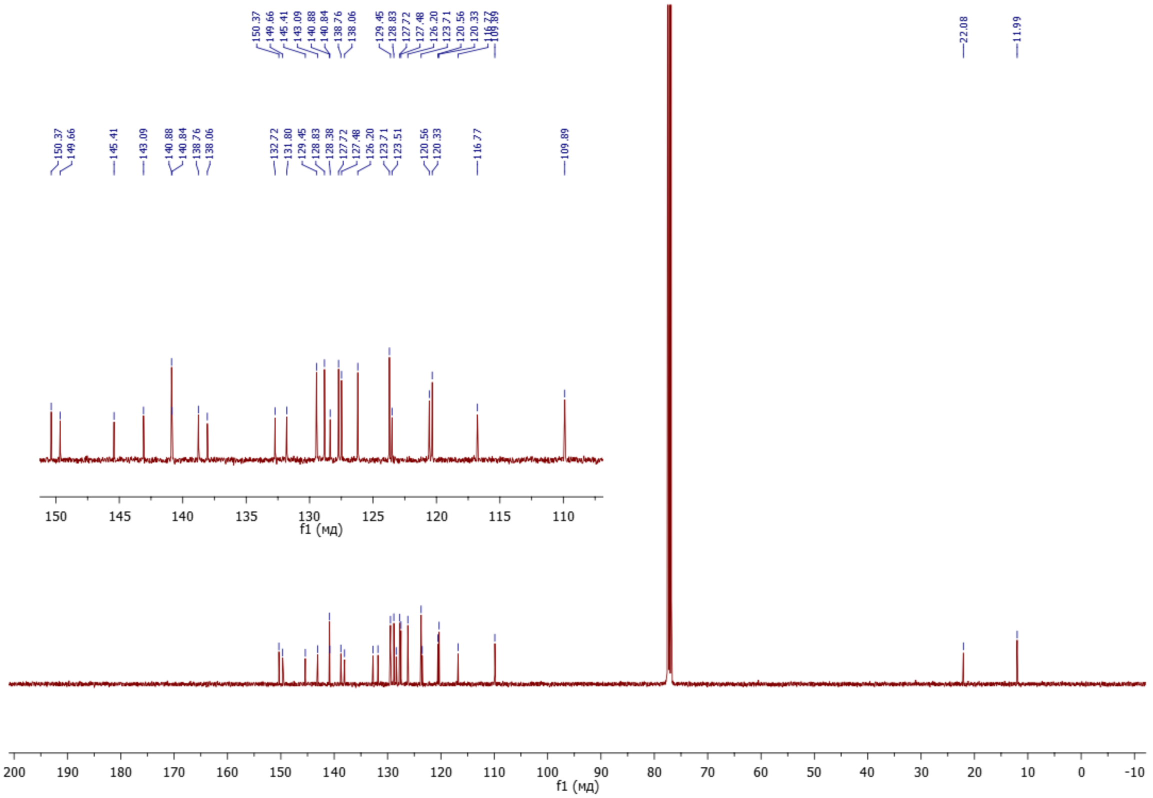

b

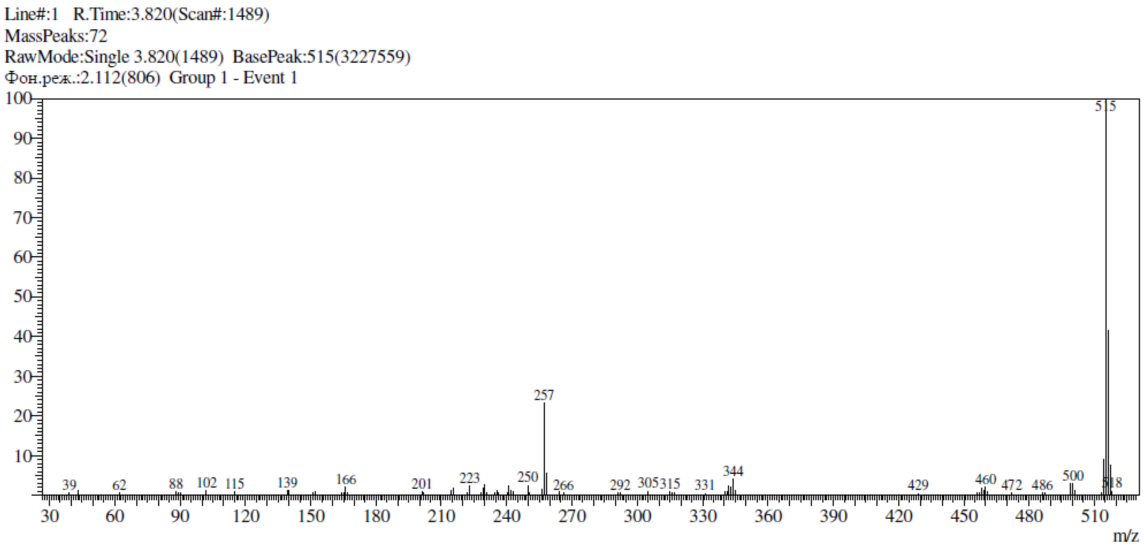

c

Figure S12. ^{1}H (a), ^{13}C (b) NMR spectra of **4f** in $DCCl_3$; mass spectrum (c) of **4f**.

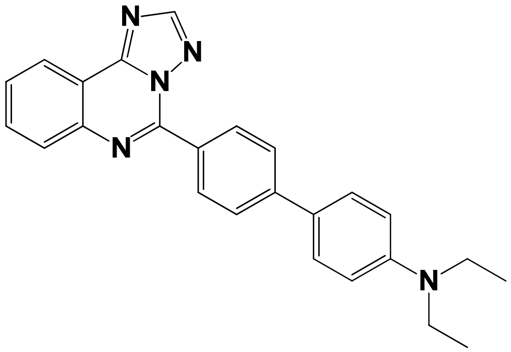

5a

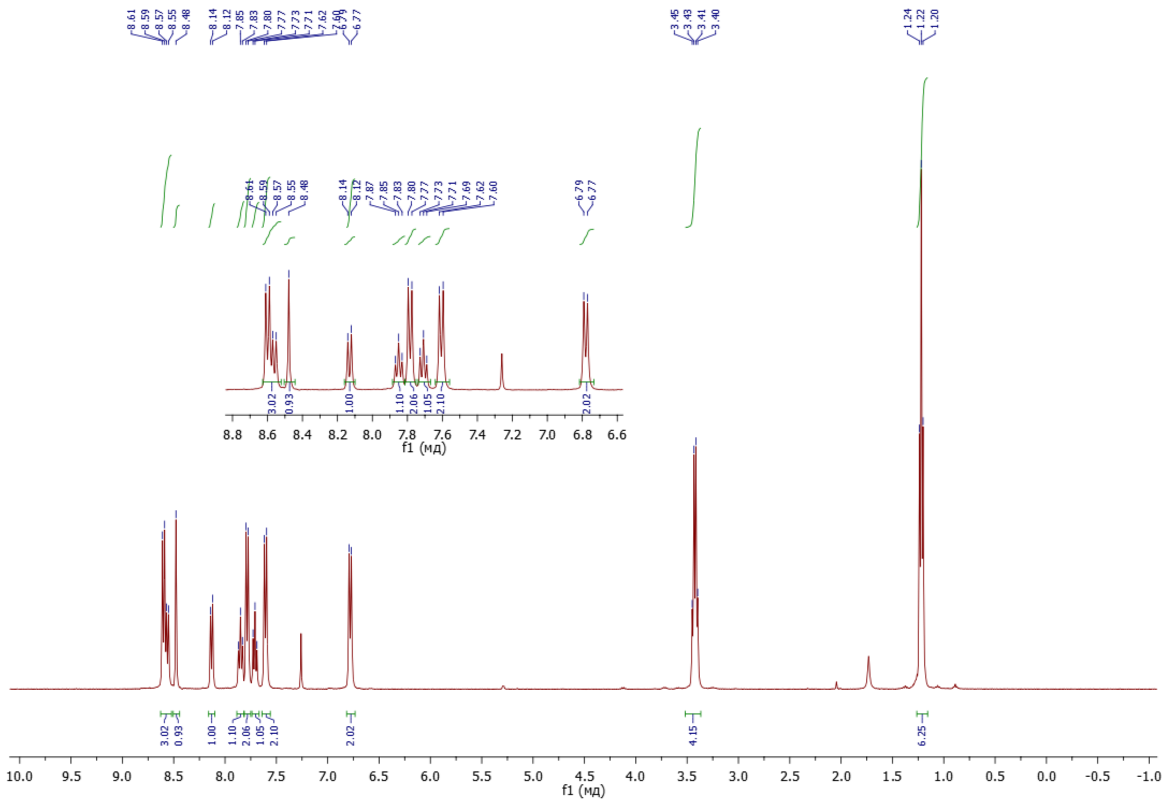

a

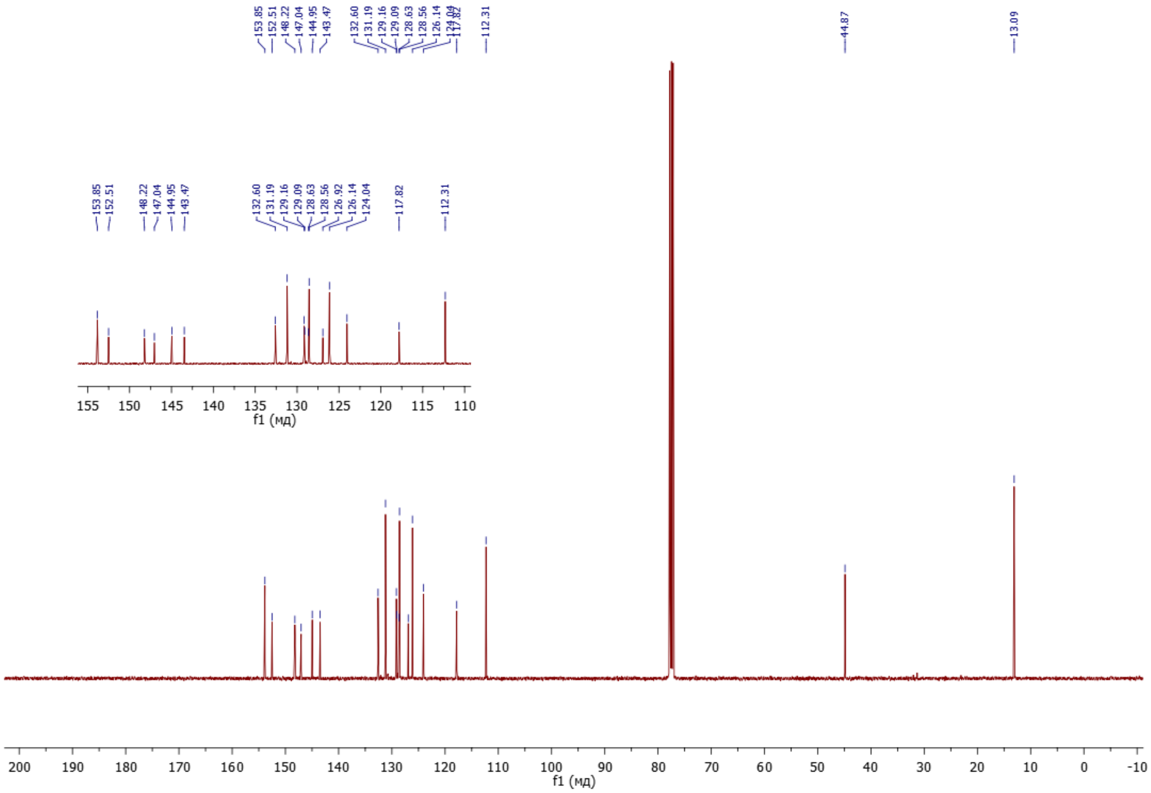

b

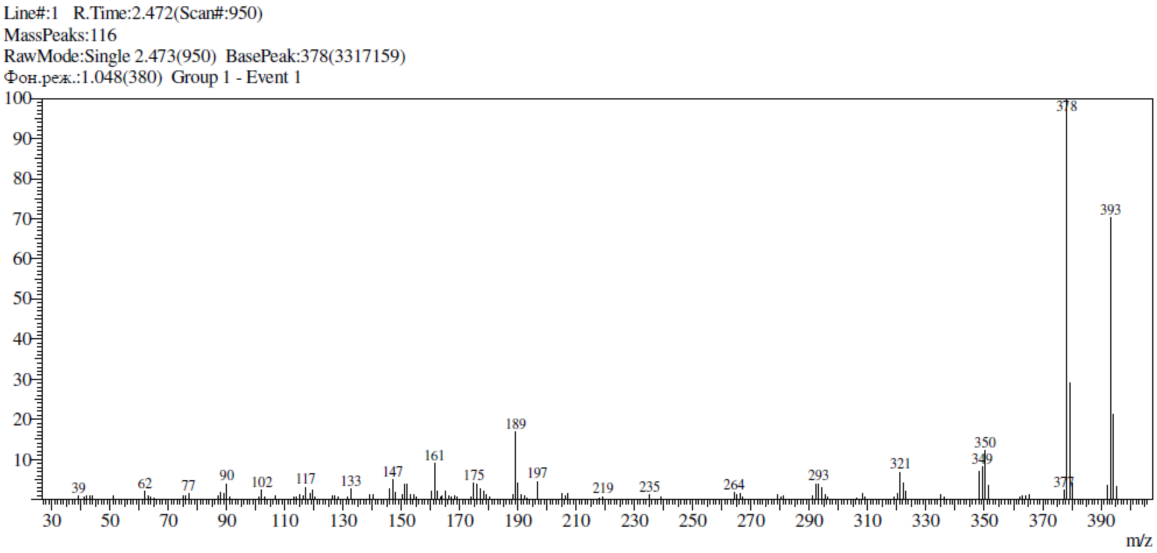

c

Figure S13. ^{1}H (a), ^{13}C (b) NMR spectra of **5a** in $DCCl_3$; mass spectrum (c) of **5a.**

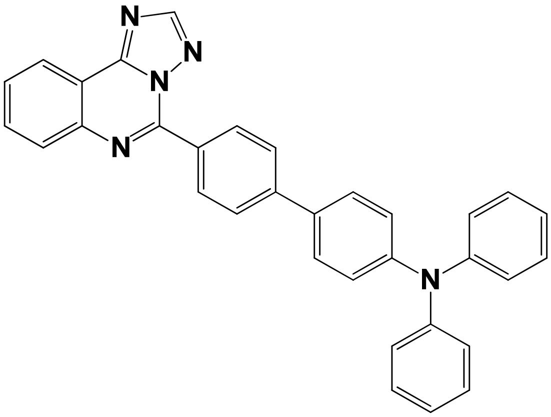

5b

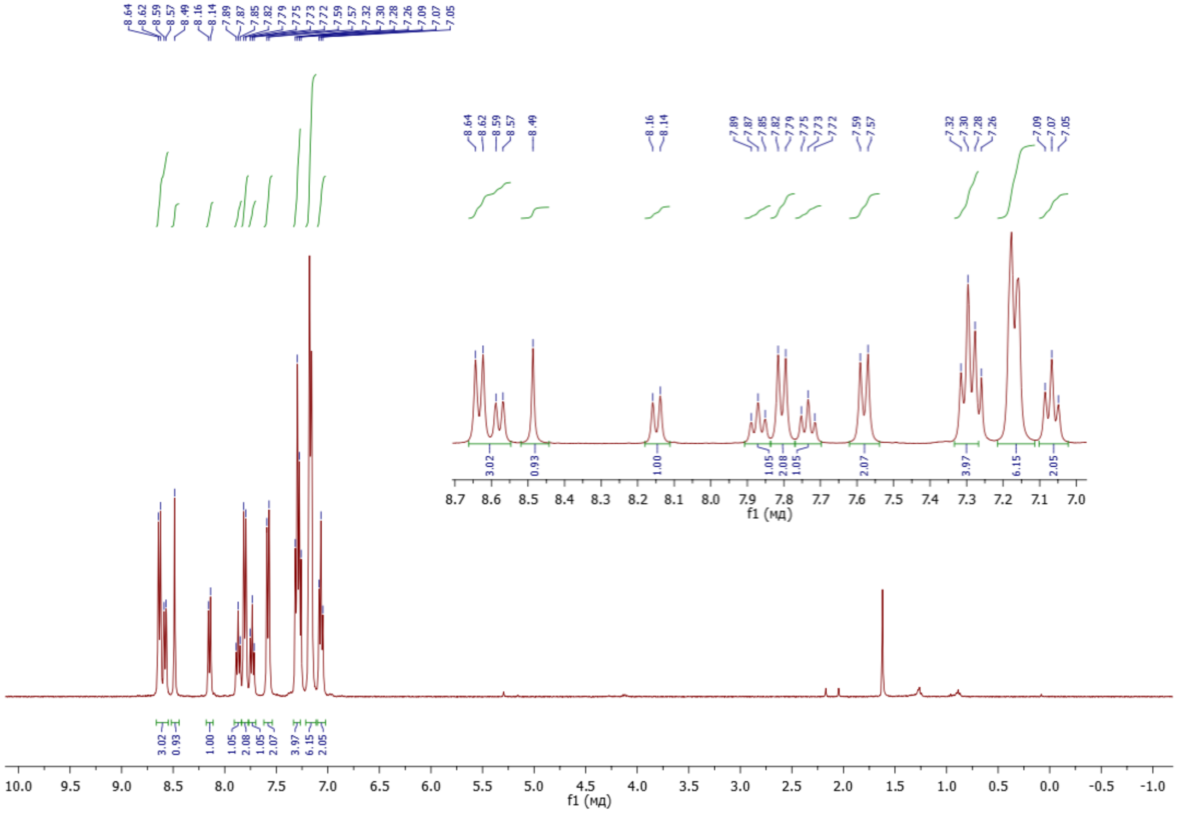

a

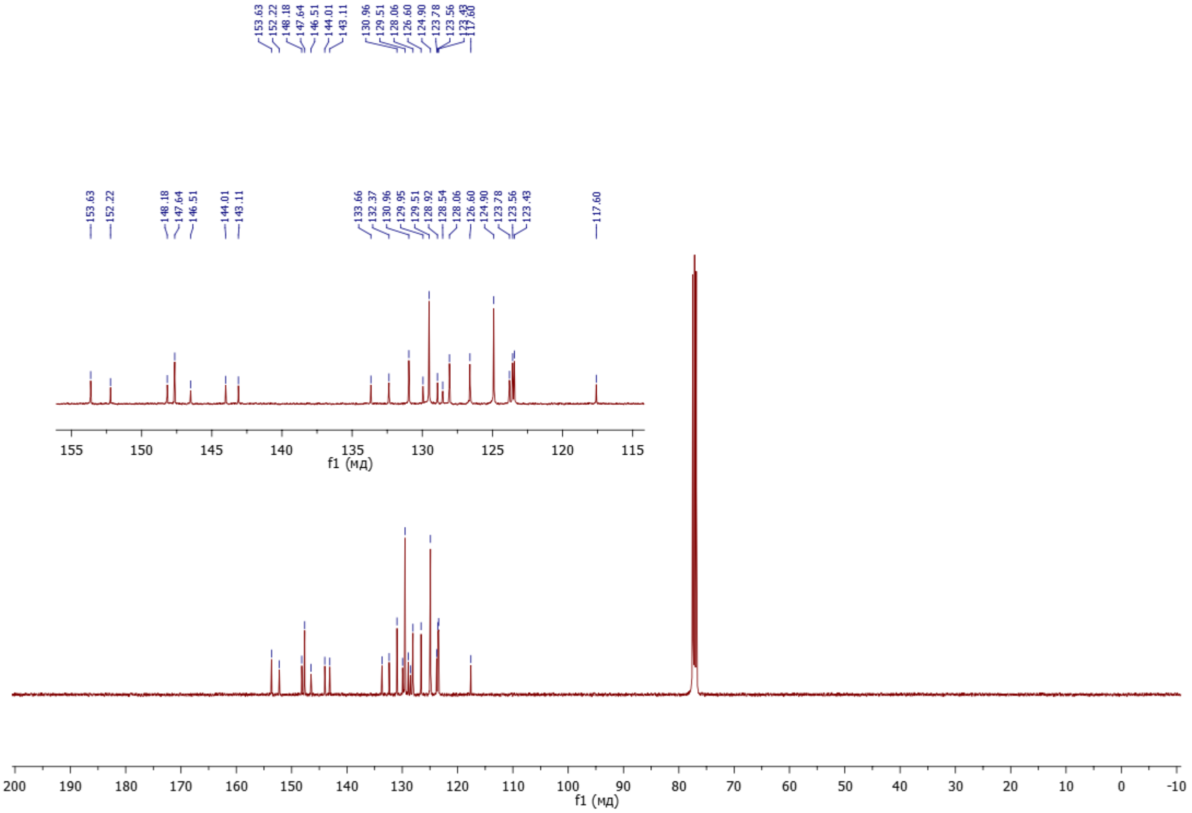

b

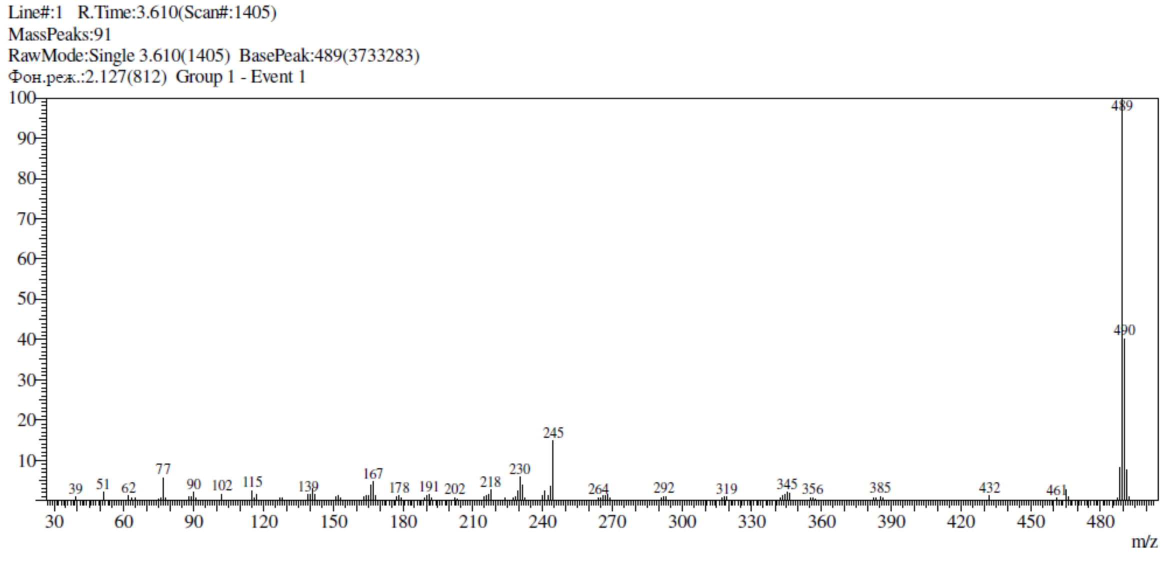

c

Figure S14. ^{1}H (a), ^{13}C (b) NMR spectra of **5b** in DCCl3; mass spectrum (c) of **5b.**

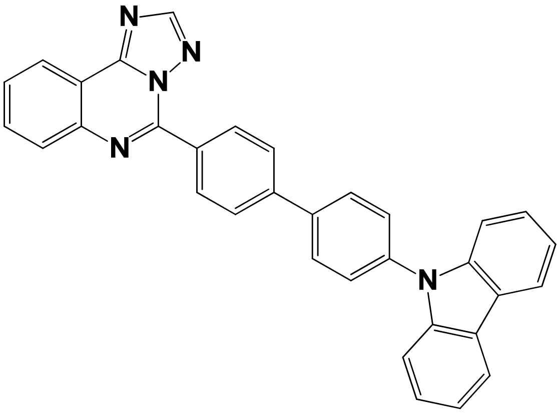

5c

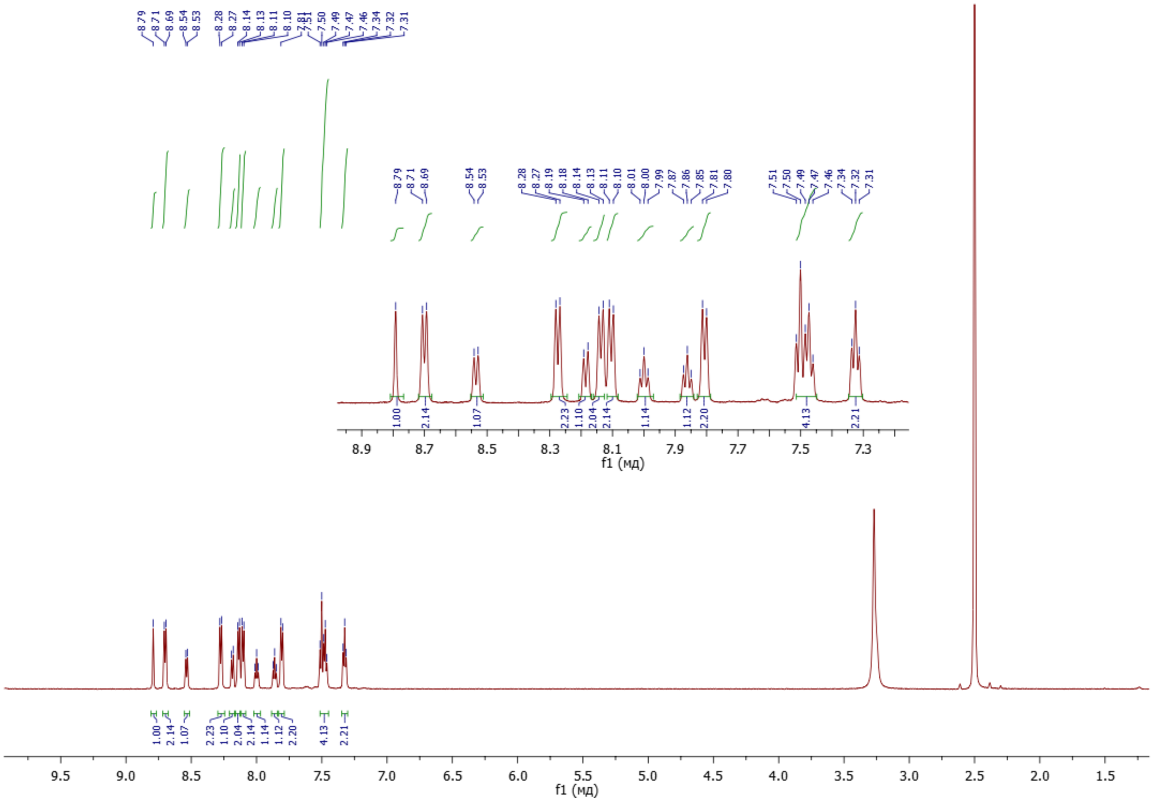

a

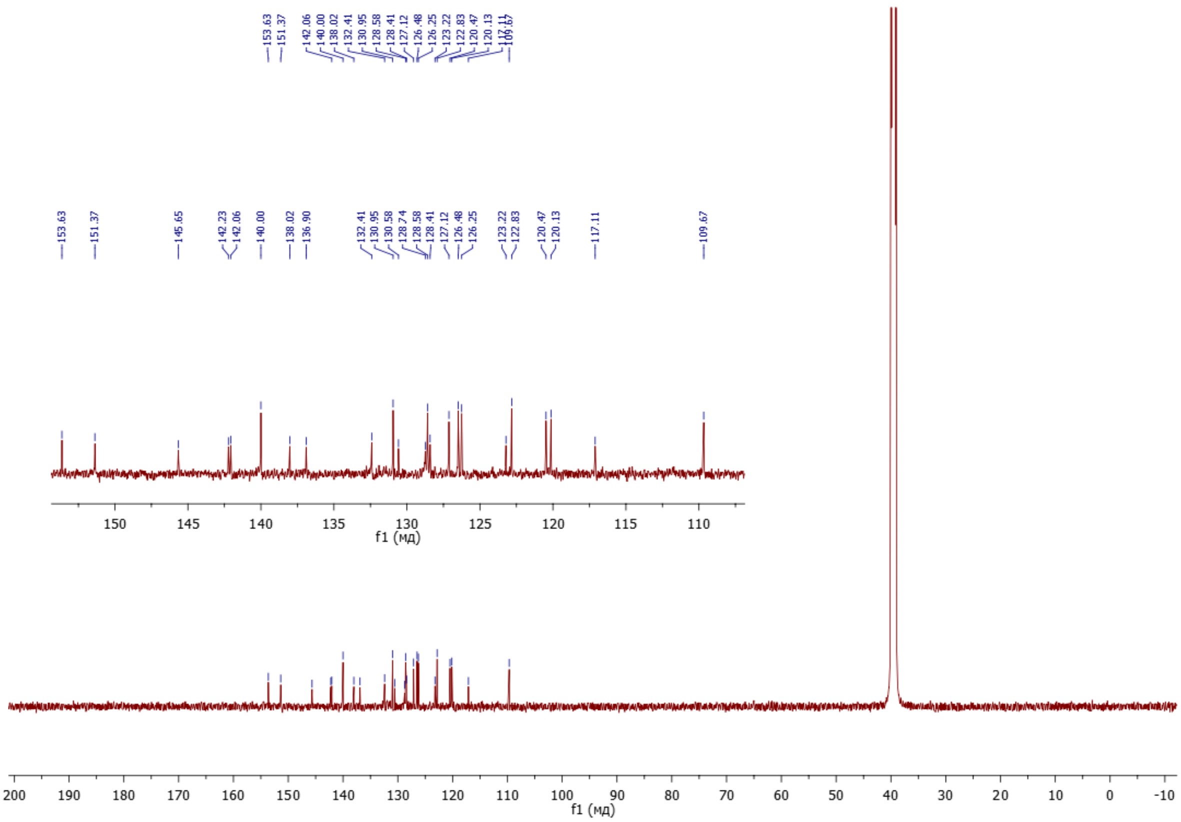

b

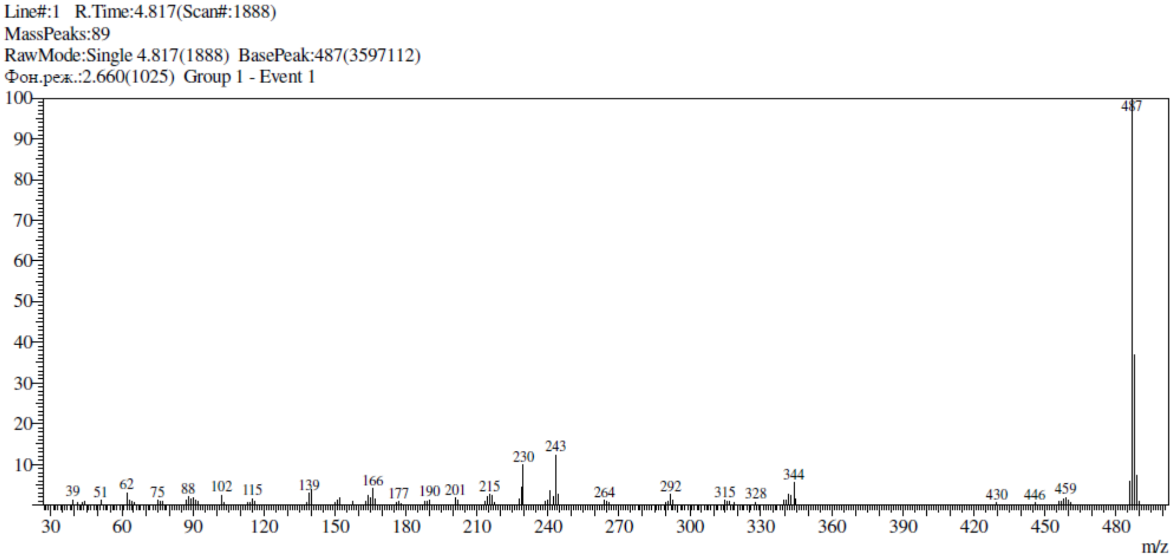

c

Figure S15. ^{1}H (a), ^{13}C (b) NMR spectra of **5c** in DMSO-d_6; mass spectrum (c) of **5c**.

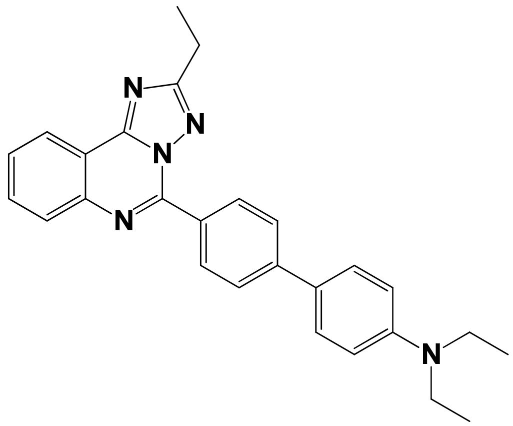

5d

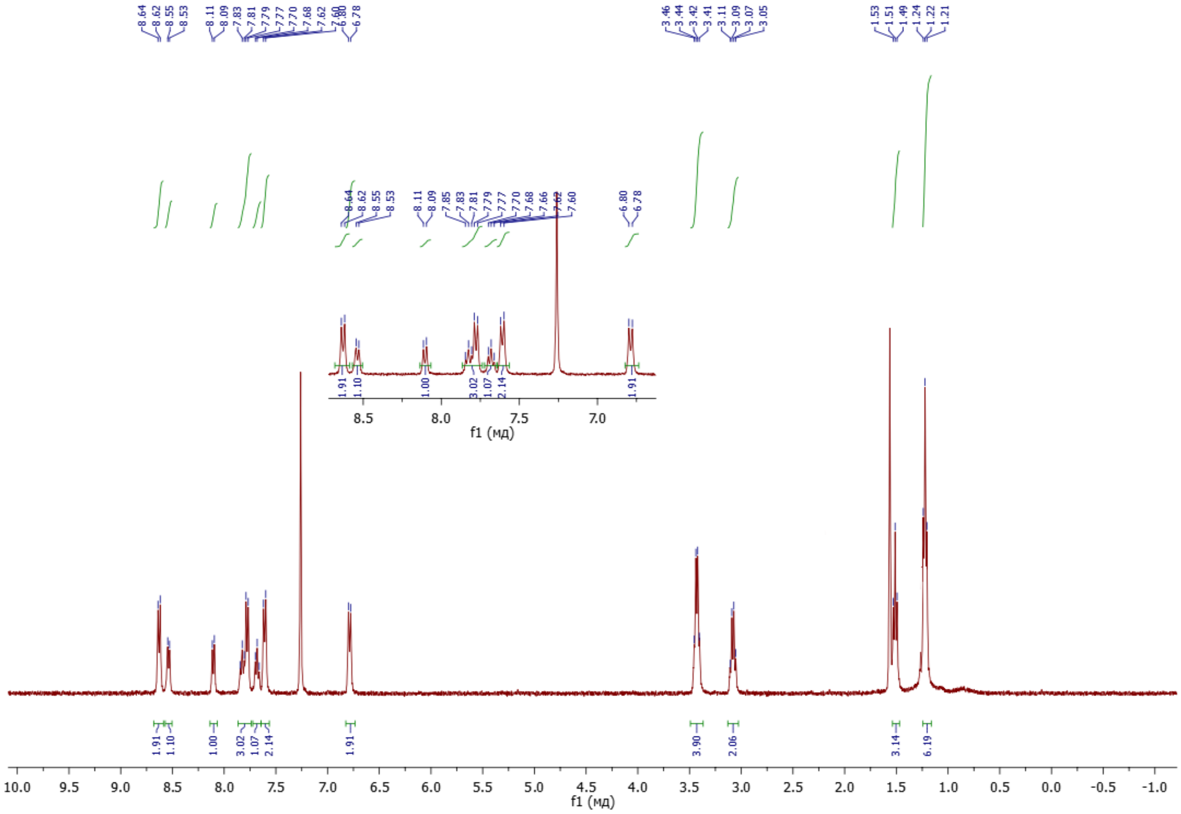

a

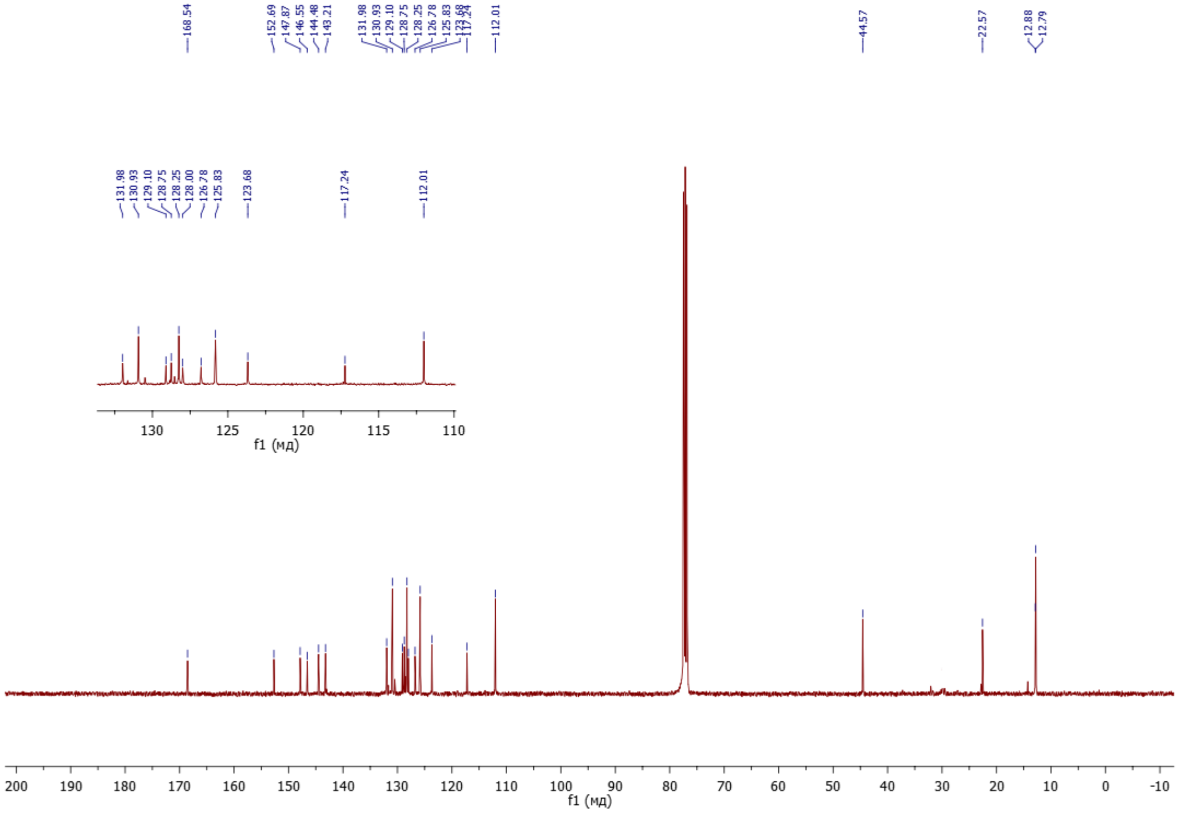

b

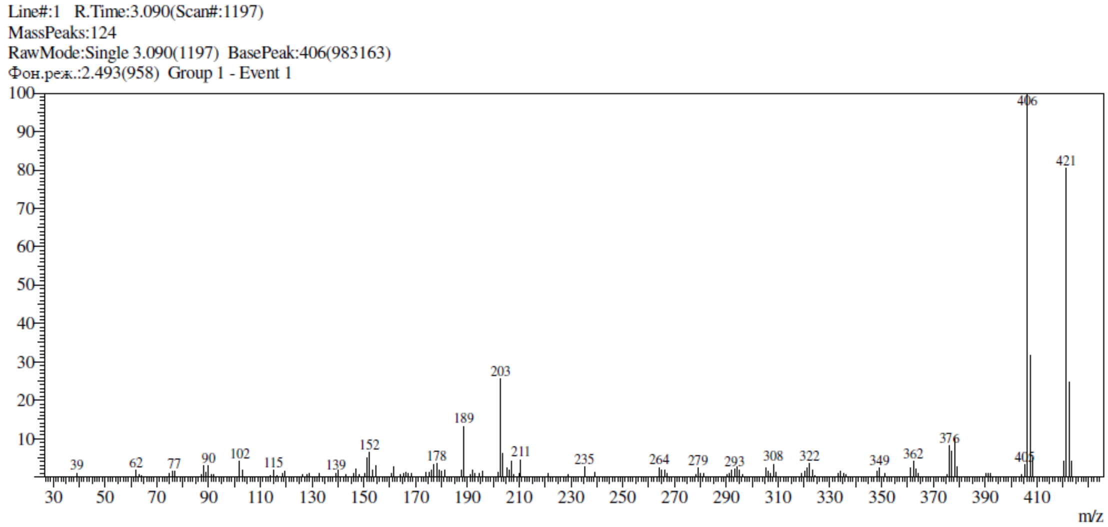

c

Figure S16. ^{1}H (a), ^{13}C (b) NMR spectra of **5d** in DCCl3; mass spectrum (c) of **5d.**

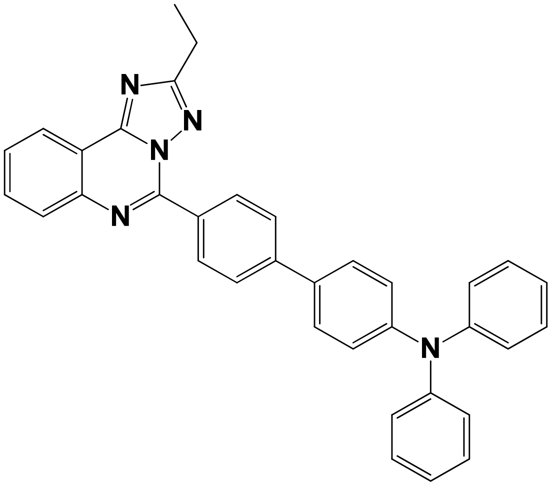

5e

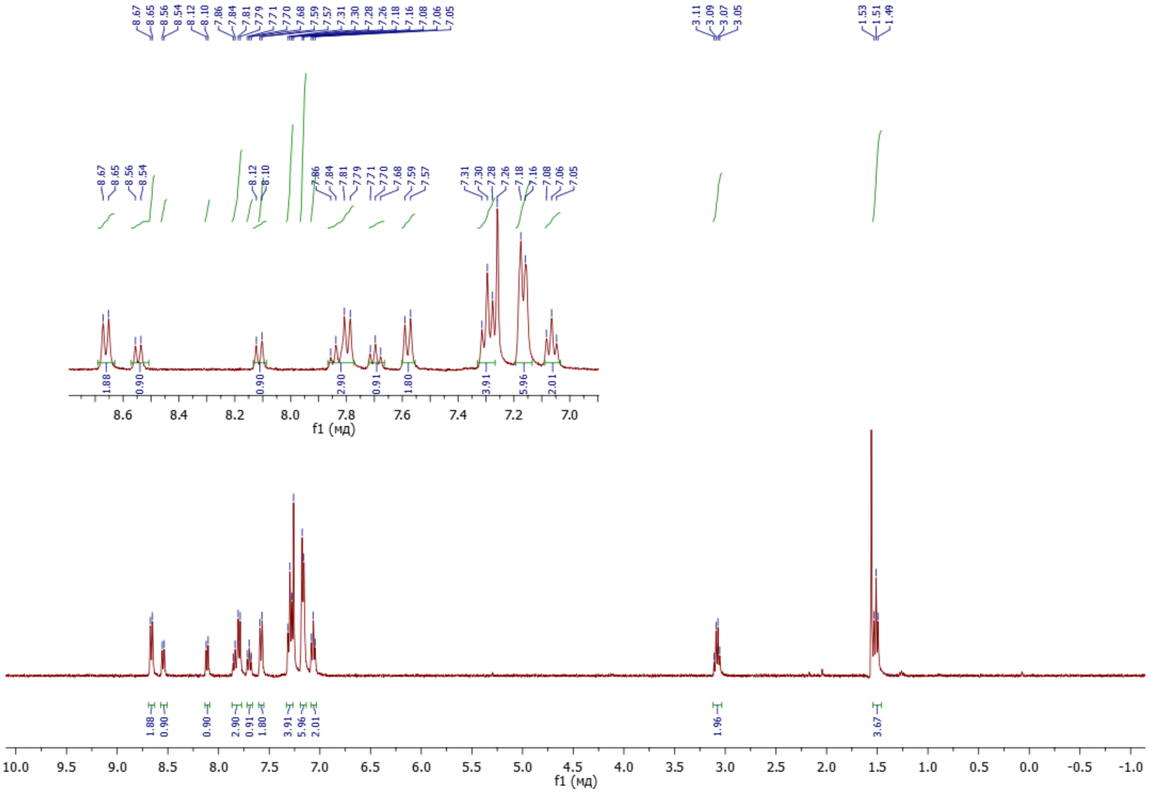

a

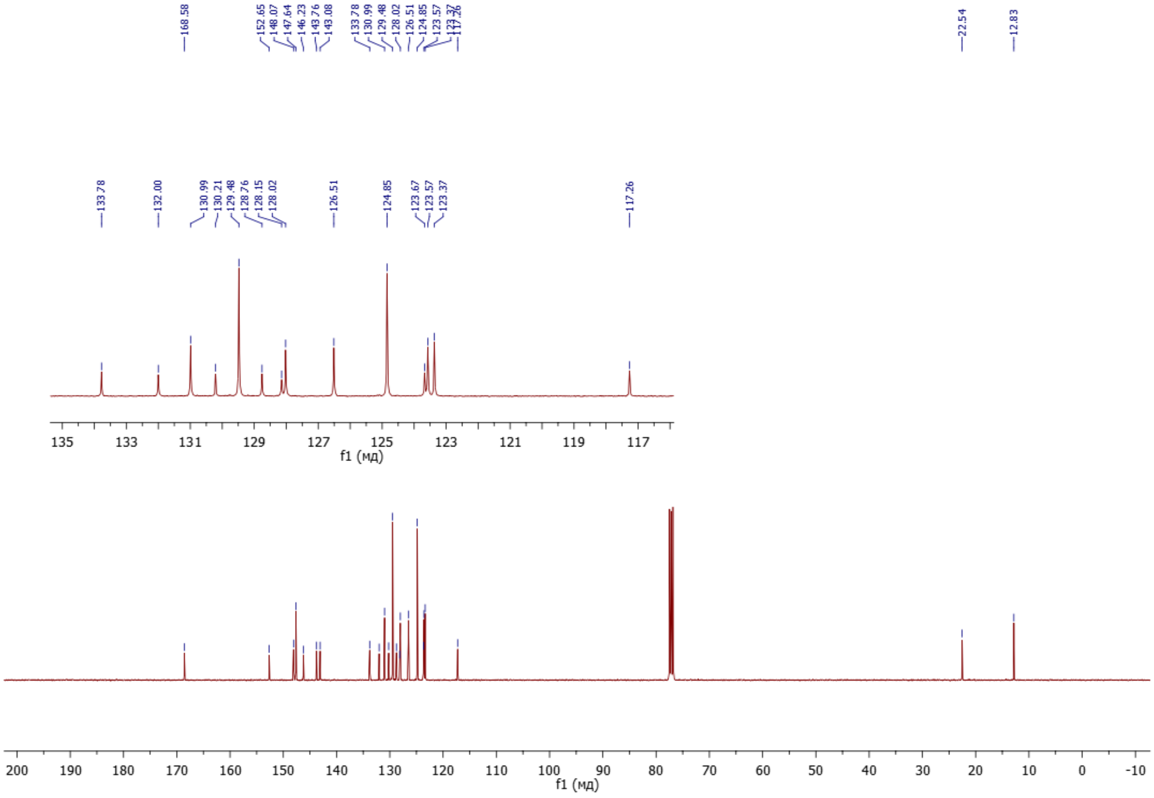

b

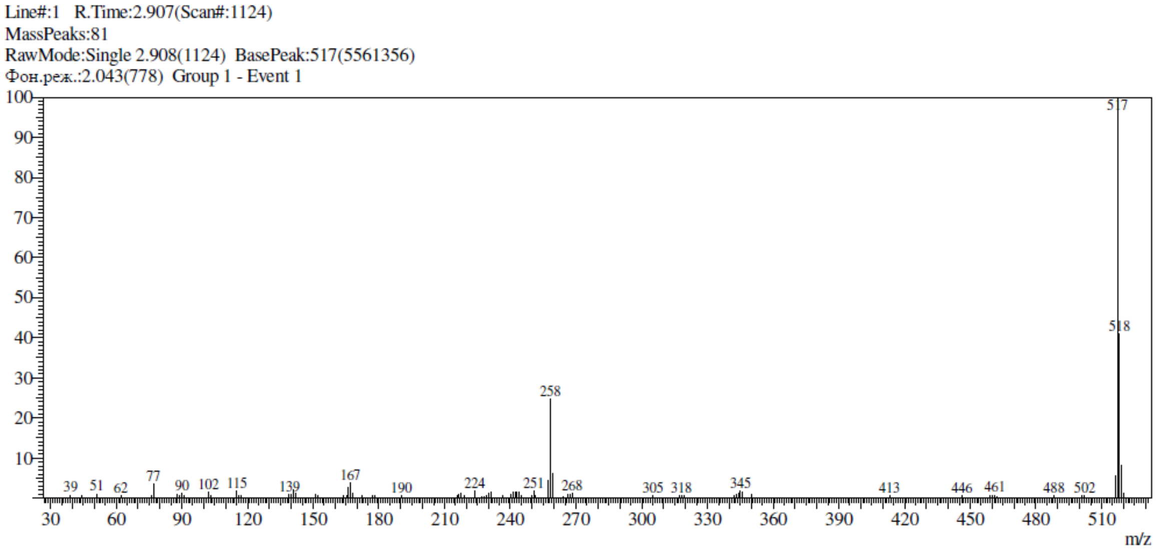

c

Figure S17. ^{1}H (a), ^{13}C (b) NMR spectra of **5e** in $DCCl_3$; mass spectrum (c) of **5e**.

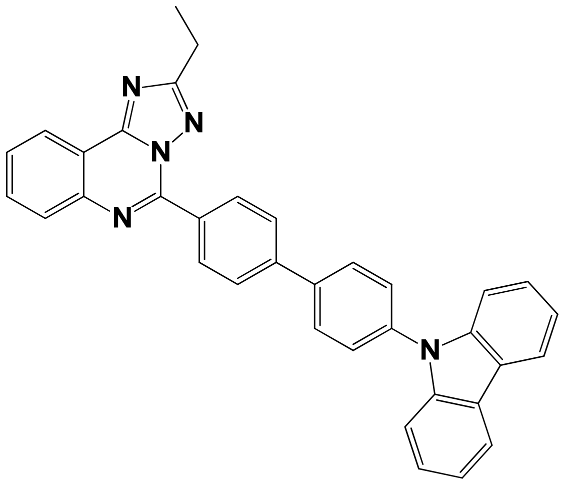

5f

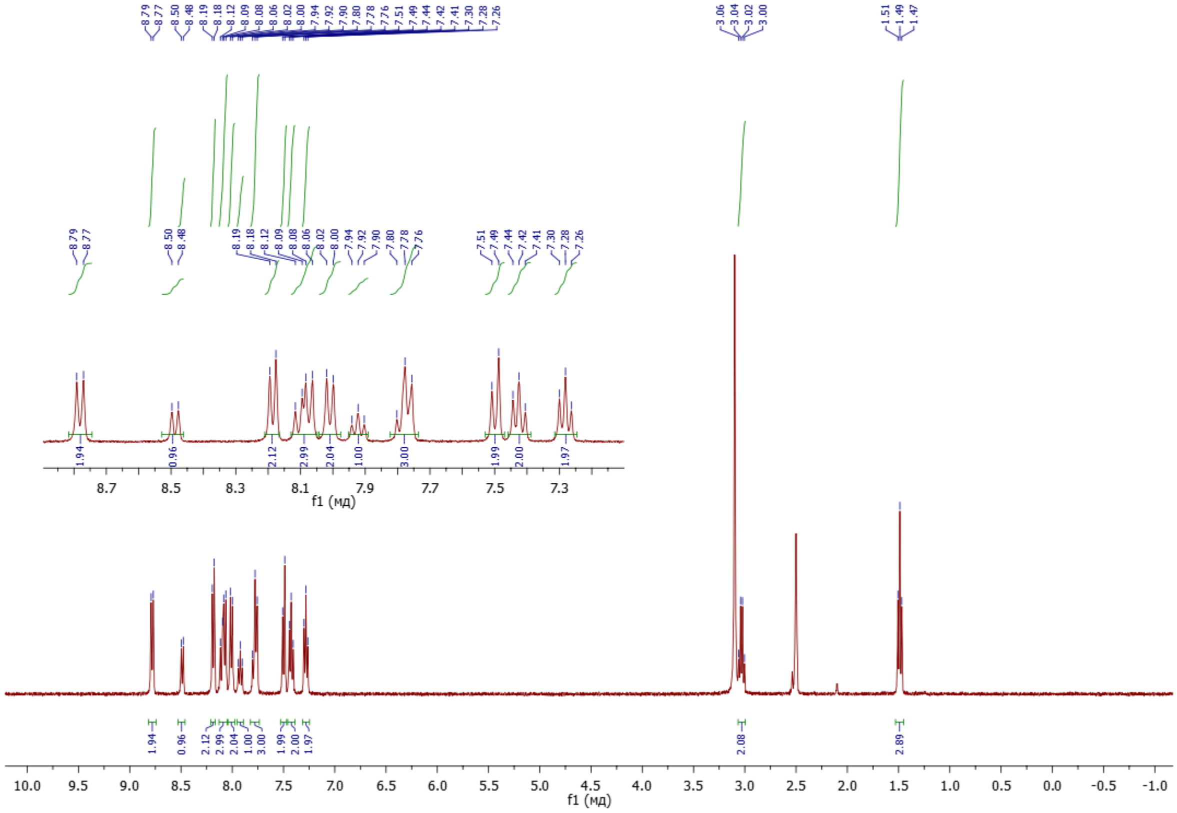

a

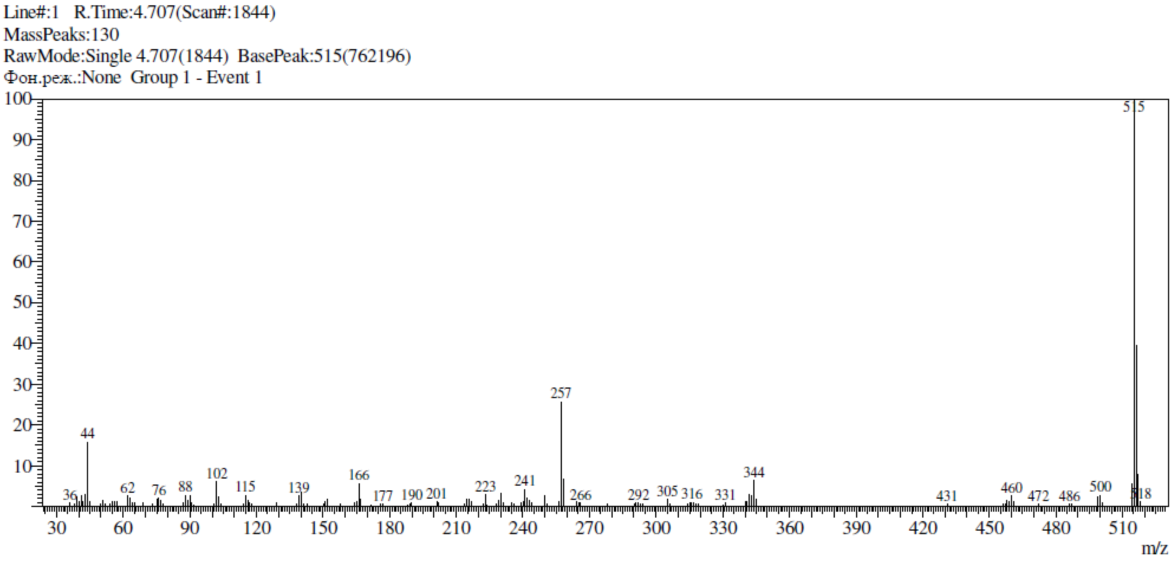

b

Figure S18. ^{1}H (a) NMR spectrum of **5f** in DMSO-d_6; mass spectrum (b) of **5f.**

2. The proposed reaction mechanism

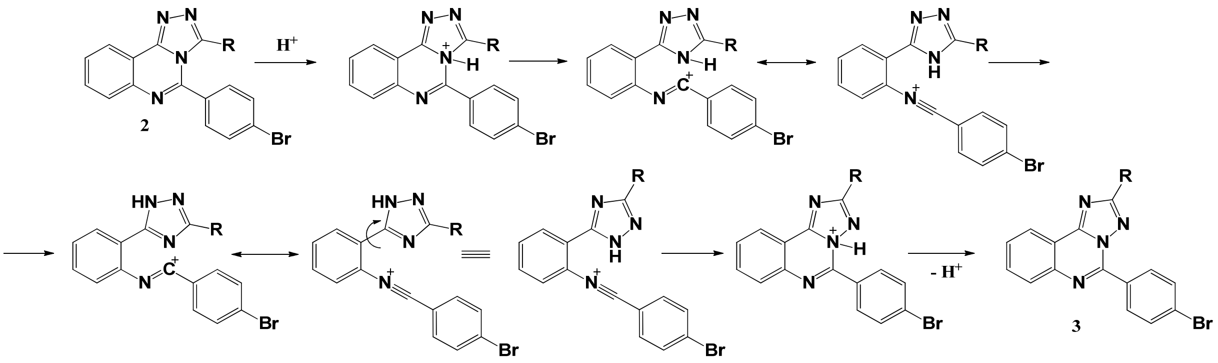

Scheme S1. The proposed mechanism of the Dimroth rearrangement [1].

3. **Crystallographic data of triazoloquinazolines**

Table S1. Selected bond lengths of compound **4a**.

**Bond**	**Bond length (Å)**	**Bond**	**Bond length (Å)**
N(3) – C(2)	1.413(2)	C(23) – C(24)	1.403(3)
N(3) – C(7)	1.380(2)	N(5) – C(4)	1.294(2)
N(3) – C(4)	1.378(2)	C(21) – C(20)	1.392(3)
N(1) – C(2)	1.289(2)	C(21) – C(22)	1.380(3)
N(1) – C(13)	1.397(2)	C(16) – C(15)	1.374(3)
N(2) – C(23)	1.376(2)	C(7) – C(8)	1.435(3)
N(2) – C(26)	1.461(3)	C(20) – C(25)	1.385(3)
N(2) – C(29)	1.462(3)	C(24) – C(25)	1.385(3)
N(6) – N(5)	1.391(2)	C(13) – C(8)	1.403(3)
N(6) – C(7)	1.310(2)	C(13) – C(12)	1.398(3)
C(14) – C(2)	1.474(3)	C(8) – C(9)	1.395(3)
C(14) – C(15)	1.394(2)	C(18) – C(19)	1.379(3)
C(14) – C(19)	1.390(3)	C(12) – C(11)	1.371(3)
C(17) – C(16)	1.387(3)	C(26) – C(28)	1.481(3)
C(17) – C(20)	1.485(3)	C(29) – C(27)	1.508(3)
C(17) – C(18)	1.397(3)	C(9) – C(10)	1.371(3)
C(23) – C(22)	1.403(3)	C(11) – C(10)	1.387(3)

Table S2. Selected bond angles of compound **4a**.

**Angle**	**(°)**	**Angle**	**(°)**
C(7) – N(3) – C(2)	121.80(16)	N(6) – C(7) – N(3)	110.89(18)
C(4) – N(3) – C(2)	134.49(17)	N(6) – C(7) – C(8)	130.6(2)
C(4) – N(3) – C(7)	103.63(16)	C(21) – C(20) – C(17)	121.8(2)
C(2) – N(1) – C(13)	120.43(17)	C(25) – C(20) – C(17)	121.50(18)
C(23) – N(2) – C(26)	121.86(19)	C(25) – C(20) – C(21)	116.63(19)
C(23) – N(2) – C(29)	121.87(17)	C(21) – C(22) – C(23)	121.54(18)
C(26) – N(2) – C(29)	115.15(18)	C(16) – C(15) – C(14)	120.57(19)
C(7) – N(6) – N(5)	106.58(17)	C(25) – C(24) – C(23)	120.6(2)
C(15) – C(14) – C(2)	123.77(19)	C(24) – C(25) – C(20)	122.66(19)
C(19) – C(14) – C(2)	118.35(17)	N(1) – C(13) – C(8)	122.66(17)
C(19) – C(14) – C(15)	117.82(18)	N(1) – C(13) – C(12)	118.34(19)
N(3) – C(2) – C(14)	119.22(17)	C(12) – C(13) – C(8)	119.00(19)
N(1) – C(2) – N(3)	120.44(17)	C(13) – C(8) – C(7)	116.22(18)
N(1) – C(2) – C(14)	120.33(18)	C(9) – C(8) – C(7)	123.4(2)
C(16) – C(17) – C(20)	121.86(17)	C(9) – C(8) – C(13)	120.32(19)
C16) – C(17) – C(18)	117.20(18)	C(19) – C(18) – C(17)	121.0(2)
C(18) – C(17) – C(20)	120.91(19)	C(18) – C(19) – C(14)	121.23(18)
N(2) – C(23) – C(22)	121.91(18)	N(5) – C(4) – N(3)	110.74(18)
N(2) – C(23) – C(24)	121.3(2)	C(11) – C(12) – C(13)	119.8(2)
C(22) – C(23) – C(24)	116.75(19)	N(2) – C(26) – C(28)	112.6(2)
C(4) – N(5) – N(6)	108.15(16)	N(2) – C(29) – C(27)	114.9(2)
C(22) – C(21) – C(20)	121.8(2)	C(10) – C(9) – C(8)	119.6(2)
C(15) – C(16) – C(17)	122.06(17)	C(12) – C(11) – C(10)	121.1(2)
N(3) – C(7) – C(8)	118.40(18)	C(9) – C(10) – C(11)	120.2(2)

Table S3. Selected bond lengths of compound **4e**.

**Bond**	**Bond length (Å)**	**Bond**	**Bond length (Å)**
N(5) – C(5)	1.387(4)	C(24) – C(25)	1.382(4)
N(5) – C(13)	1.401(4)	C(27) – C(32)	1.384(5)
N(5) – C(2)	1.394(4)	C(27) – C(28)	1.378(5)
N(12) – C(13)	1.287(4)	C(17) – C(16)	1.371(4)
N(12) – C(11)	1.400(4)	C(2) – C(1)	1.481(5)
N(2) – C(24)	1.415(4)	C(11) – C96)	1.395(5)
N(2) – C(27)	1.411(4)	C(11) – C(10)	1.391(5)
N(2) – C(38)	1.416(4)	C(26) – C(25)	1.386(4)
N(3) – N(4)	1.398(4)	C(38) – C(33)	1.386(4)
N(3) – C(2)	1.315(4)	C(38) – C(37)	1.368(5)
N(4) – C(5)	1.307(4)	C(6) – C(7)	1.402(4)
C(21) – C(18)	1.484(4)	C(1) – C(14)	1.493(5)
C(21) – C(22)	1.391(4)	C(32) – C(31)	1.384(5)
C(21) – C(26)	1.376(4)	C(10) – C(9)	1.376(5)
C(18) – C(19)	1.389(4)	C(7) – C(8)	1.356(5)
C(18) – C(17)	1.395(4)	C(8) – C(9)	1.368(6)
C(22) – C(23)	1.383(4)	C(33) – C(34)	1.400(5)
C(15) – C(13)	1.482(4)	C(34) – C(35)	1.349(6)
C(15) – C(20)	1.384(4)	C(28) – C(29)	1.382(5)
C(15) – C(16)	1.373(4)	C(30) – C(31)	1.328(6)
C(5) – C(6)	1.422(5)	C(30) – C(29)	1.354(7)
C(19) – C(20)	1.374(4)	C(37) – C(36)	1.378(5)
C(24) – C(23)	1.384(4)	C(35) – C(36)	1.360(6)

Table S4. Selected bond angles of compound **2e**.

**Angle**	**(°)**	**Angle**	**(°)**
C(5) – N(5) – C(13)	121.0(3)	C(16) – C(17) – C(18)	121.1(3)
C(5) – N(5) – C(2)	104.6(3)	N(5) – C(2) – C(1)	127.0(3)
C(2) – N(5) – C(13)	134.4(3)	N(3) – C(2) – N(5)	109.0(3)
C(13) – N(12) – C(11)	119.4(3)	N(3) – C(2) – C(1)	123.9(3)
C(24) – N(2) – C(38)	119.7(2)	C(6) – C(11) – N(12)	122.5(3)
C(27) – N(2) – C(24)	119.2(2)	C(10) – C(11) – N(12)	118.0(3)
C(27) – N(2) – C(38)	121.1(3)	C(10) – C(11) – C(6)	119.5(3)
C(2) – N(3) – N(4)	108.6(3)	C(21) – C(26) – C(25)	122.0(3)
C(5) – N(4) – N(3)	107.0(3)	C(33) – C(38) – N(2)	121.1(3)
C(22) – C(21) – C(18)	121.1(3)	C(37) – C(38) – N(2)	120.8(3)
C(26) – C(21) – C(18)	122.3(3)	C(37) – C(38) – C(33)	118.1(3)
C(26) – C(21) – C(22)	116.5(3)	C(22) – C(23) – C(24)	120.0(3)
C(19) – C(18) – C(21)	121.7(3)	C(17) – C(16) – C(15)	121.4(3)
C(19) – C(18) – C(17)	116.8(3)	C(11) – C(6) – C(5)	117.0(3)
C(17) – C(18) – C(21)	121.4(3)	C(11) – C(6) – C(7)	119.4(3)
C(23) – C(22) – C(21)	122.4(3)	C(7) – C(6) – C(5)	123.5(3)
C(20) – C(15) – C(13)	121.8(3)	C(24) – C(25) – C(26)	120.7(3)
C(16) – C(15) – C(13)	119.9(3)	C(2) – C(1) – C(14)	113.1(3)
C(16) – C(15) – C(20)	118.3(3)	C(31) – C(32) – C(27)	119.5(4)
N(5) – C(5) – C(6)	118.1(3)	C(9) – C(10) – C(11)	119.3(4)
N(4) – C(5) – N(5)	110.7(3)	C(8) – C(7) – C(6)	120.0(4)
N(4) – C(5) – C(6)	131.0(3)	C(7) – C(8) – C(9)	120.6(4)

C(20) – C(19) – C(18)	121.8(3)	C(38) – C(33) – C(34)	120.0(3)
N(5) – C(13) – C(15)	117.9(3)	C(35) – C(34) – C(33)	120.6(4)
N(12) – C(13) – N(5)	121.7(3)	C(27) – C(28) – C(29)	119.9(4)
N(12) – C(13) – C(15)	120.4(3)	C(8) – C(9) – C(10)	121.1(4)
C(23) – C(24) – N(2)	120.0(3)	C(31) – C(30) – C(29)	120.4(4)
C(25) – C(24) – N(2)	121.6(3)	C(30) – C(31) – C(32)	121.2(4)
C(25) – C(24) – C(23)	118.3(3)	C(38) – C(37) – C(36)	120.8(4)
C(19) – C(20) – C(15)	120.4(3)	C(34) – C(35) – C(36)	119.3(4)
C(32) – C(27) – N(2)	119.7(3)	C(35) – C(36) – C(37)	121.1(4)
C(28) – C(27) – N(2)	121.6(3)	C(30) – C(29) – C(28)	120.4(4)
C(28) – C(27) – C(32)	118.6(3)		

Table S5. Selected bond lengths of compound **5d**.

**Bond**	**Bond length (Å)**	**Bond**	**Bond length (Å)**
N(3) – N(4)	1.376(2)	N(2) – C(25)	1.373(3)
N(3) – C(7)	1.374(2)	N(2) – C(1)	1.438(3)
N(3) – C(2)	1.395(2)	N(2) – C(4)	1.607(6)
N(1) – C(2)	1.303(2)	C(17) – C(18)	1.374(3)
N(1) – C(13)	1.387(2)	C(8) – C(13)	1.405(3)
N(6) – C(7)	1.324(2)	C(8) – C(9)	1.397(3)
N(6) – C(5)	1.364(3)	C(13) – C(12)	1.401(3)
N(4) – C(5)	1.321(2)	C(5) – C(14)	1.495(3)
C(16) – C(2)	1.472(3)	C(27) – C(26)	1.374(3)
C(16) – C(21)	1.392(3)	C(9) – C(10)	1.369(3)
C(16) – C(17)	1.397(3)	C(25) – C(24)	1.402(3)
C(19) – C(20)	1.395(2)	C(25) – C(26)	1.402(3)
C(19) – C(22)	1.478(3)	C(24) – C(23)	1.376(3)
C(19) – C(18)	1.403(3)	C(10) – C(11)	1.396(3)
C(20) – C(21)	1.375(2)	C(11) – C(12)	1.365(3)
C(7) – C(8)	1.428(3)	C(14) – C(15)	1.490(4)
C(22) – C(27)	1.391(3)	C(1) – C(3)	1.499(4)
C(22) – C(23)	1.391(3)	C(4) – C(6)	1.508(7)

Table S6. Selected bond angles of compound **5d**.

**Angle**	**(°)**	**Angle**	**(°)**
N(4) – N(3) – C(2)	127.16(15)	C(18) – C(17) – C(16)	121.48(19)
C(7) – N(3) – N(4)	109.48(15)	C(13) – C(8) – C(7)	115.61(17)
C(7) – N(3) – C(2)	123.20(16)	C(9) – C(8) – C(7)	124.08(18)
C(2) – N(1) – C(13)	120.09(17)	C(9) – C(8) – C(13)	120.24(18)
C(7) – N(6) – C(5)	103.21(16)	C(17) – C(18) – C(19)	121.52(18)
C(5) – N(4) – N(3)	101.93(15)	N(1) – C(13) – C(8)	123.32(18)
C(21) – C(16) – C(2)	124.04(17)	N(1) – C(13) – C(12)	118.26(18)
C(21) – C(16) – C(17)	117.43(17)	C(12) – C(13) – C(8)	118.42(18)
C(17) – C(16) – C(2)	118.50(18)	N(6) – C(5) – C(14)	122.57(19)
C(20) – C(19) – C(22)	121.48(17)	N(4) – C(5) – N(6)	115.84(18)
C(20) – C(19) – C(18)	116.25(17)	N(4) – C(5) – C(14)	121.6(2)
C(18) – C(19) – C(22)	122.27(17)	C(26) – C(27) – C(22)	122.63(18)
C(21) – C(20) – C(19)	122.52(18)	C(10) – C(9) – C(8)	119.8(2)
N(3) – C(7) – C(8)	118.17(17)	N(2) – C(25) – C(24)	121.84(18)
N(6) – C(7) – N(3)	109.54(17)	N(2) – C(25) – C(26)	122.30(19)

N(6) – C(7) – C(8)	132.23(18)	C(24) – C(25) – C(26)	115.86(18)
N(3) – C(2) – C(16)	120.44(17)	C(23) – C(24) – C(25)	121.36(18)
N(1) – C(2) – N(3)	119.49(17)	C(24) – C(23) – C(22)	122.97(18)
N(1) – C(2) – C(16)	120.07(17)	C(9) – C(10) – C(11)	120.4(2)
C(27) – C(22) – C(19)	121.93(17)	C(27) – C(26) – C(25)	121.77(19)
C(27) – C(22) – C(23)	115.40(17)	C(12) – C(11) – C(10)	120.2(2)
C(23) – C(22) – C(19)	122.66(17)	C(15) – C(14) – C(5)	115.4(2)
C(25) – N(2) – C(1)	121.96(19)	C(11) – C(12) – C(13)	120.9(2)
C(25) – N(2) – C(4)	117.6(2)	N(2) – C(1) – C(3)	114.0(2)
C(1) – N(2) – C(4)	116.8(2)	C(6) – C(4) – N(2)	105.8(5)
C(20) – C(21) – C(16)	120.75(17)		

Table S7. Selected bond lengths of compound **5e**.

**Bond**	**Bond length (Å)**	**Bond**	**Bond length (Å)**
C(1) – C(2)	1.480(6)	C(15) – C(16)	1.372(3)
C(1) – C(3)	1.484(3)	C(16) – C(17)	1.395(3)
C(1) – C(4)	1.560(17)	C(17) – C(18)	1.391(3)
N(1) – N(2)	1.371(2)	C(17) – C(20)	1.476(3)
N(1) – C(5)	1.362(3)	C(18) – C(19)	1.384(3)
N(1) – C(13)	1.387(3)	C(20) – C(21)	1.387(3)
N(2) – C(3)	1.322(3)	C(20) – C(25)	1.387(3)
C(3) – N(4)	1.369(3)	C(21) – C(22)	1.375(3)
N(3) – C(23)	1.409(3)	C(22) – C(23)	1.384(3)
N(3) – C(26)	1.419(3)	C(23) – C(24)	1.387(3)
N(3) – C(32)	1.425(3)	C(24) – C(25)	1.374(3)
N(4) – C(5)	1.323(3)	C(26) – C(27)	1.384(4)
C(5) – C(6)	1.418(3)	C(26) – C(31)	1.381(4)
C(6) – C(7)	1.397(3)	C(27) – C(28)	1.376(4)
C(6) – C(11)	1.401(3)	C(28) – C(29)	1.367(5)
C(7) – C(8)	1.366(4)	C(29) – C(30)	1.376(5)
C(8) – C(9)	1.390(4)	C(30) – C(31)	1.369(4)
C(9) – C(10)	1.362(4)	C(32) – C(33)	1.378(3)
C(10) – C(11)	1.404(3)	C(32) – C(37)	1.371(3)
C(11) – N(12)	1.383(3)	C933) – C(34)	1.379(4)
N(12) – C(13)	1.293(3)	C(34) – C(35)	1.365(4)
C(13) – C(14)	1.469(3)	C(35) – C(36)	1.360(4)
C(14) – C(15)	1.386(3)	C(36) – C(37)	1.374(4)

Table S8. Selected bond angles of compound **5d**.

**Angle**	**(°)**	**Angle**	**(°)**
C(2) – C(1) – C(3)	111.6(3)	C(16) – C(15) – C(14)	121.5(2)
C(3) – C(1) – C(4)	111.4(8)	C15) – C16) – C(17)	120.7(2)
N(2) – N(1) – C(13)	127.13(19)	C(16) – C(17) – C(20)	120.6(2)
C(5) – N(1) – N(2)	109.72(18)	C(18) – C(17) – C(16)	117.4(2)
C(5) – N(1) – C(13)	123.07(18)	C(18) – C(17) – C(20)	122.0(2)
C(3) – N(2) – N(1)	101.91(19)	C(19) – C(18) – C(17)	121.8(2)
N(2) – C(3) – C(1)	121.8(2)	C(14) – C(19) – C(18)	120.1(2)
N(2) – C(3) – N(4)	115.6(2)	C(21) – C(20) – C(17)	120.66(19)
N(4) – C(3) – C(1)	122.6(2)	C(21) – C(20) – C(25)	116.9(2)
C(23) – N(3) – C(26)	121.25(19)	C(25) – C(20) – C(17)	122.4(2)

C(23) – N(3) – C(32)	118.4(2)	C(22) – C(21) – C(20)	121.8(2)
C(26) – N(3) – C(32)	118.4(2)	C(21) – C(22) – C(23)	120.8(2)
C(5) – N(4) – C(3)	102.8(2)	C(22) – C(23) – N(3)	121.9(2)
N(1) – C(5) – C(6)	118.4(2)	C(22) – C(23) – C(24)	117.9(2)
N(4) – C(5) – N(1)	109.99(19)	C(24) – C(23) – N(3)	120.2(2)
N(4) – C(5) – C(6)	131.6(2)	C(25) – C(24) – C(23)	120.9(2)
C(7) – C(6) – C(5)	124.1(2)	C(24) – C(25) – C(20)	121.7(2)
C(7) – C(6) – C(11)	120.1(2)	C(27) – C(26) – N(3)	120.5(2)
C(11) – C(6) – C(5)	115.8(2)	C(31) – C(26) – N(3)	120.8(2)
C(8) – C(7) – C(6)	119.6(2)	C(31) – C(26) – C(27)	118.6(3)
C(7) – C(8) – C(9)	120.7(3)	C(28) – C(27) – C(26)	120.1(3)
C(10) – C(9) – C(8)	120.7(2)	C(29) – C(28) – C(27)	121.1(3)
C(9) – C(10) – C(11)	120.0(2)	C(28) – C(29) – C(30)	118.7(3)
C(6) – C(11) – C(10)	118.9(2)	C(31) – C(30) – C(29)	121.0(3)
N(12) – C(11) – C(6)	122.8(2)	C(30) – C(31) – C(26)	120.5(3)
N(12) – C(11) – C(10)	118.3(2)	C(33) – C(32) – N(3)	120.7(2)
C(13) – N(12) – C(11)	120.3(2)	C(37) – C(32) – N(3)	120.5(2)
N(1) – C(13) – C(14)	120.94(19)	C(37) – C(32) – C(33)	118.8(2)
N(12) – C(13) – N(1)	119.6(2)	C(32) – C(33) – C(34)	120.4(3)
N(12) – C(13) – C(14)	119.4(2)	C(35) – C(34) – C(33)	120.1(3)
C(15) – C(14) – C(13)	116.9(2)	C(36) – C(35) – C(34)	119.8(3)
C(19) – C(14) – C(13)	124.6(2)	C(35) – C(36) – C(37)	120.4(3)
C(19) – C(14) – C(15)	118.5(2)	C(32) – C(37) – C(36)	120.5(2)

4. Absorption and emission spectra of fluorophores in toluene and MeCN

<table>
<tr><th>Compound</th><th>Toluene</th><th>MeCN</th></tr>
<tr><td>4a</td><td>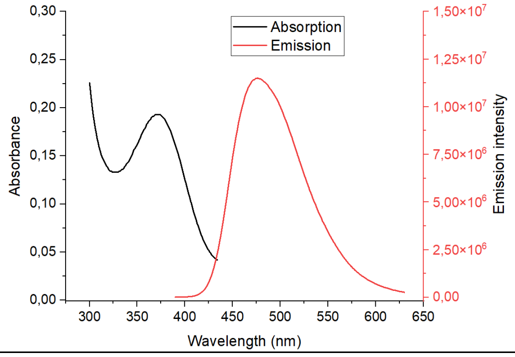
</td><td>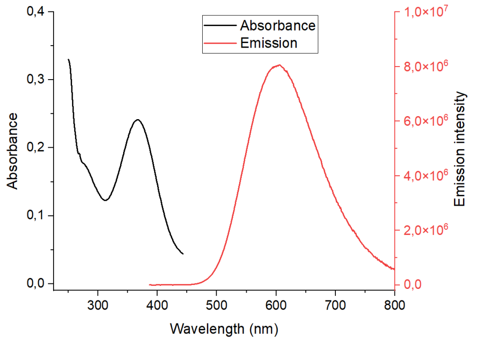
</td></tr>
<tr><td>4b</td><td>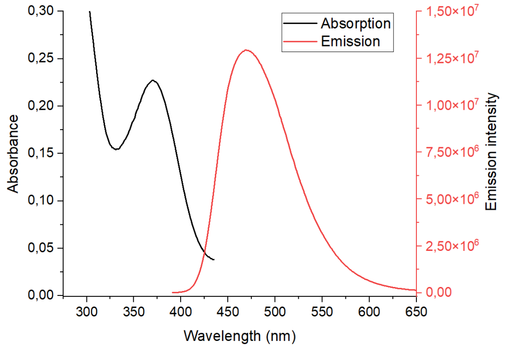
</td><td>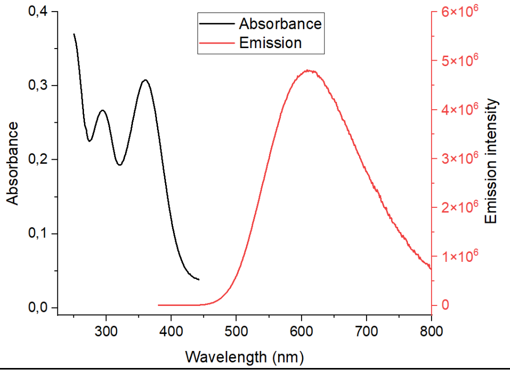
</td></tr>
<tr><td>4c</td><td>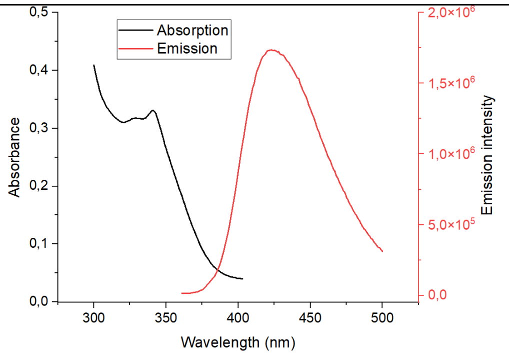
</td><td>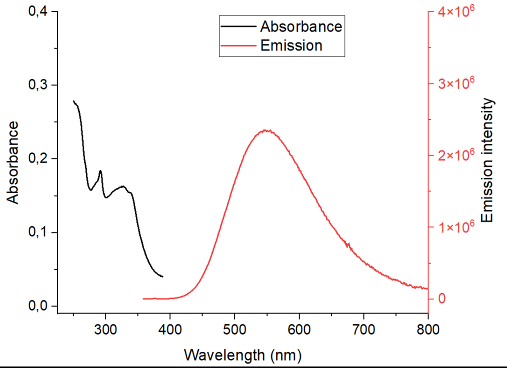
</td></tr>
<tr><td>4d</td><td>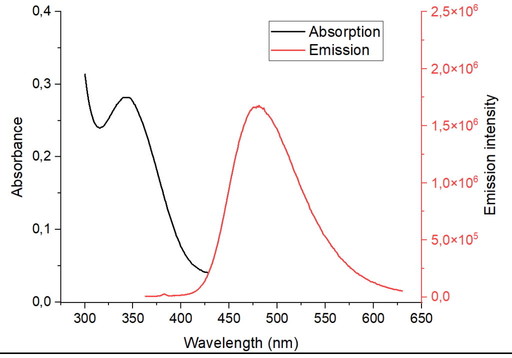
</td><td>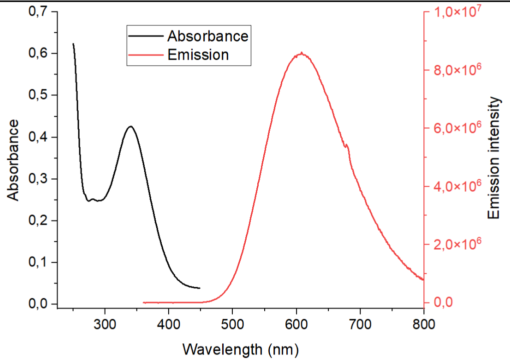
</td></tr>
</table>

<table>
<tr><td>4e</td><td>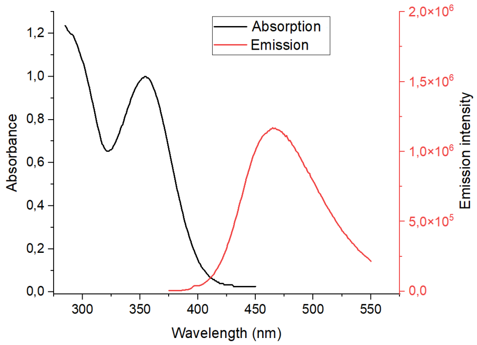
</td><td>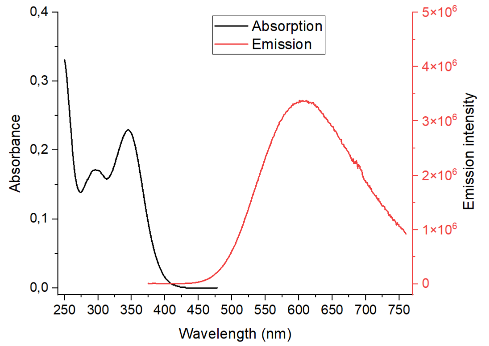
</td></tr>
<tr><td>4f</td><td>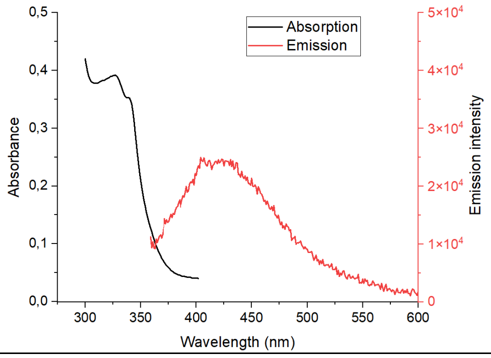
</td><td>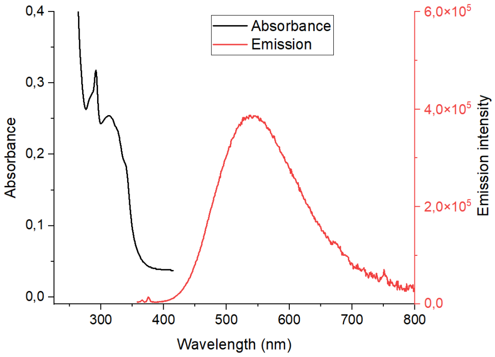
</td></tr>
<tr><td>5a</td><td>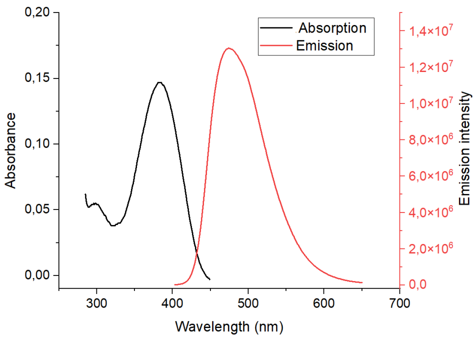
</td><td>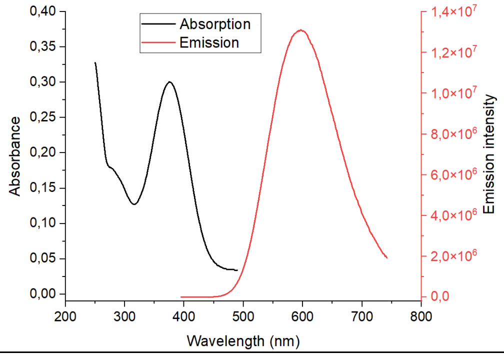
</td></tr>
<tr><td>5b</td><td>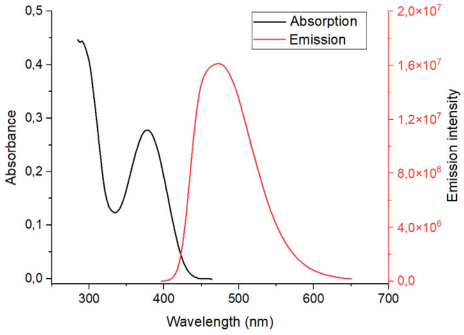
</td><td>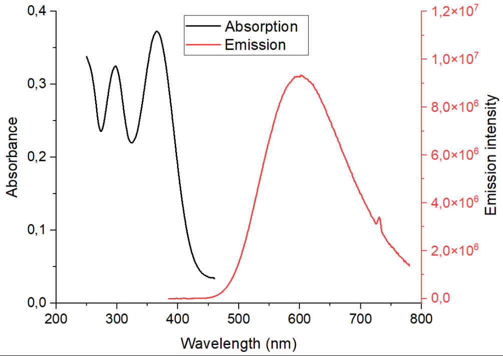
</td></tr>
</table>

5c

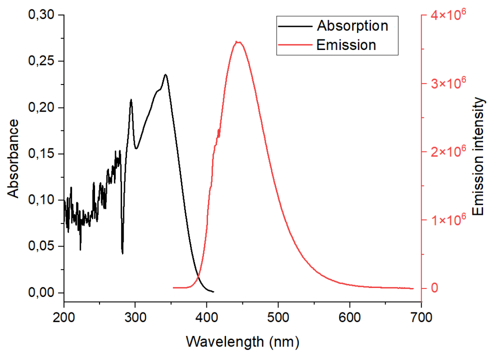

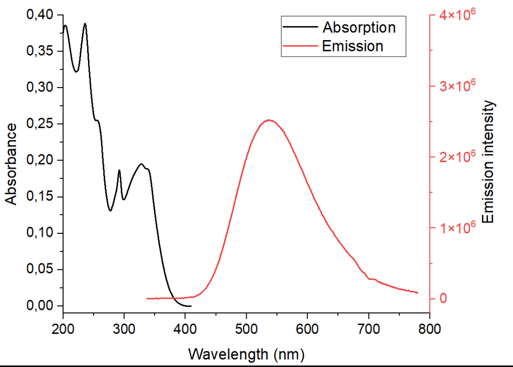

5d

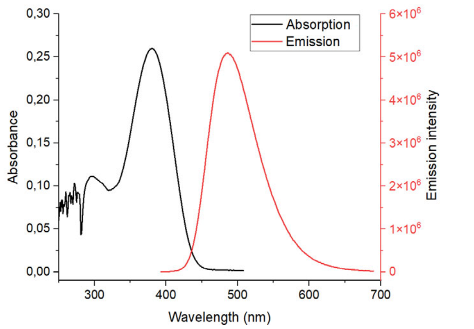

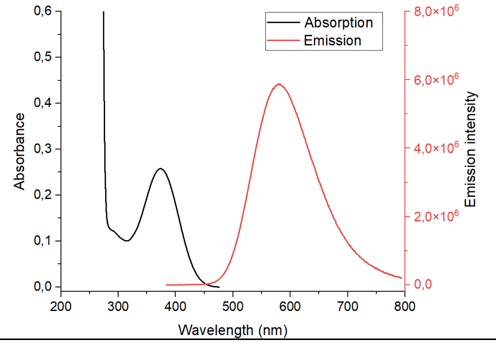

5e

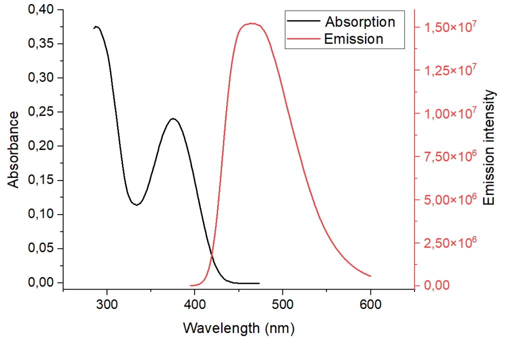

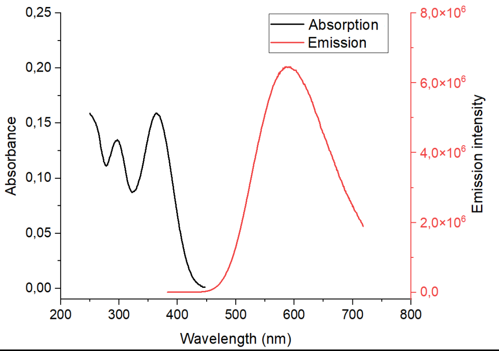

5f

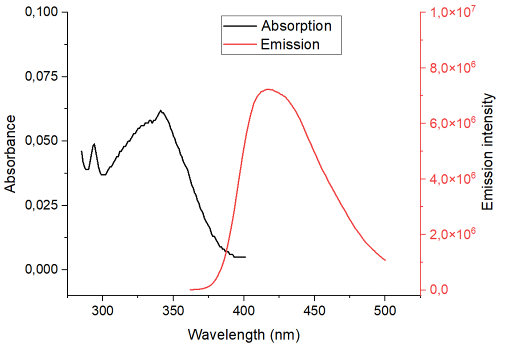

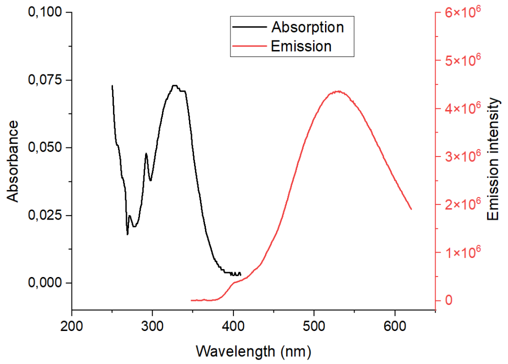

Figure S19. Absorption and emission spectra of compounds **4a-f** and **5a-f** in toluene and in MeCN

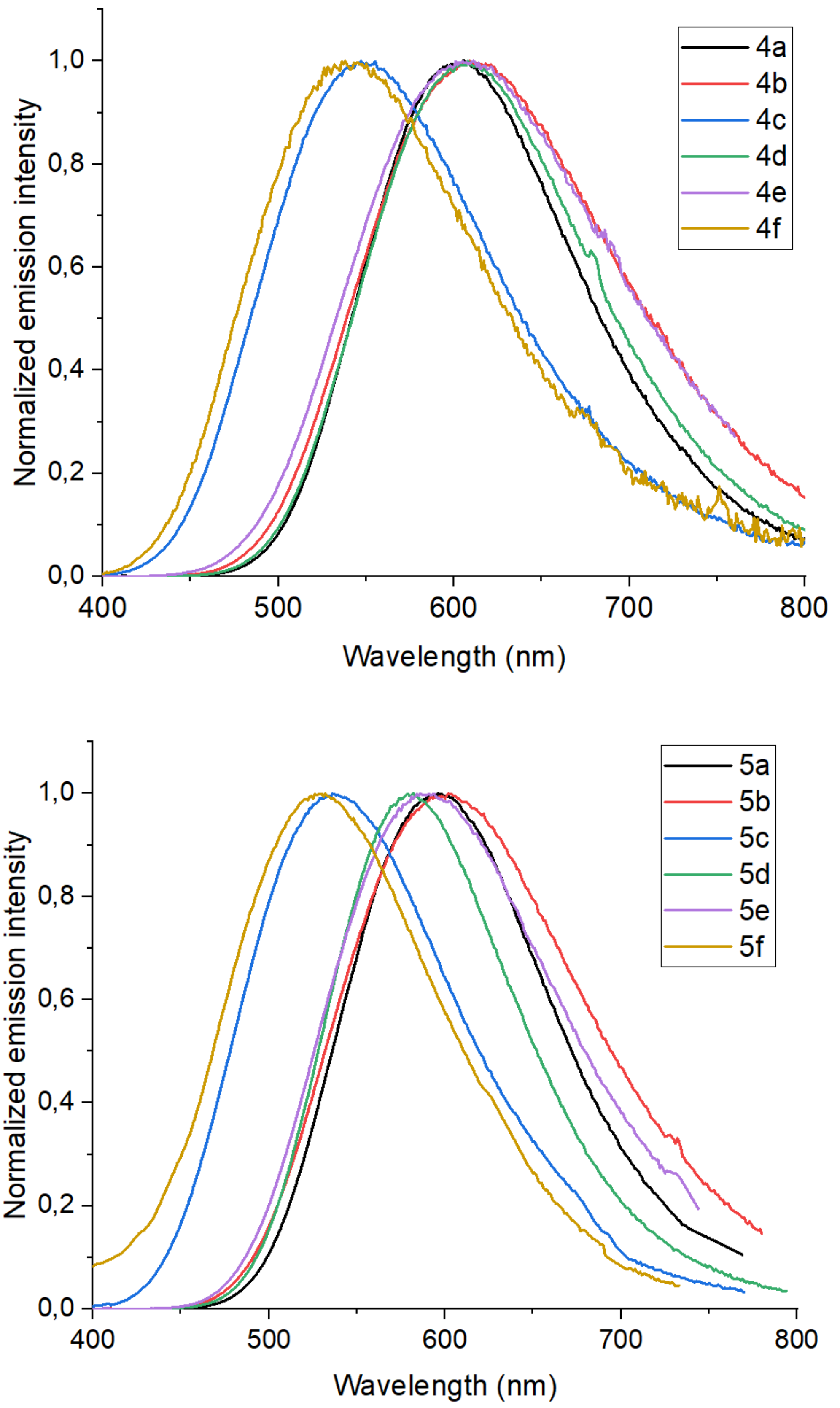

Figure S20. Combined normalized emission spectra of compounds **4a-f** and **5a-f** in MeCN.

Table S9. Detailed data of the fluorescence lifetime measurements of **4a-f** and **5a-f** (C = 2×10^{-6} M) in toluene.

Compound	τ_1, ns[a]	α_1[b]	τ_2, ns[a]	α_2[b]	τ, ns[a]	χ^2[d]
**4a**	1.737249	100			**1.73**	0.8548584
**4b**	1.852698	100			**1.85**	0.9048021
**4c**	0.136076	73.45	1.492942	26.55	**0.49**	1.246706
**4d**	0.220293	100			**0.22**	1.251333
**4e**	0.422897	17.77	1.696579	82.23	**1.47**	1.037248
**5a**	1.682252	100			**1.68**	0.9323888
**5b**	1.746369	100			**1.74**	0.9317798
**5c**	1.261517	100			**1.26**	0.990872
**5d**	1.604809	100			**1.60**	0.9300374
**5e**	1.663215	100			**1.66**	0.8256598
**5f**	1.112374	100			**1.11**	0.7840446

[a] Decay time, [b] Fractional contribution, [c] Weighted average decay time $\tau_{av} = \Sigma (\tau_i \times \alpha_i)$, [d] Quality of fitting

4a

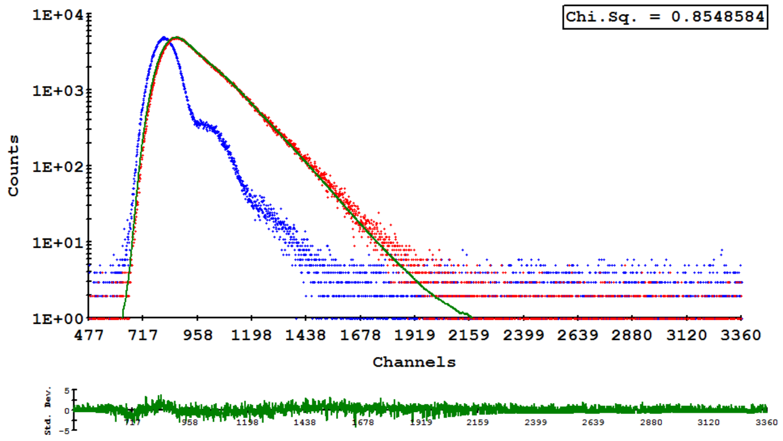
Chi.Sq. = 0.8548584
1E+04
1E+03
1E+02
1E+01
1E+00
Counts
477 717 958 1198 1438 1678 1919 2159 2399 2639 2880 3120 3360
Channels
Std. Dev.

4b

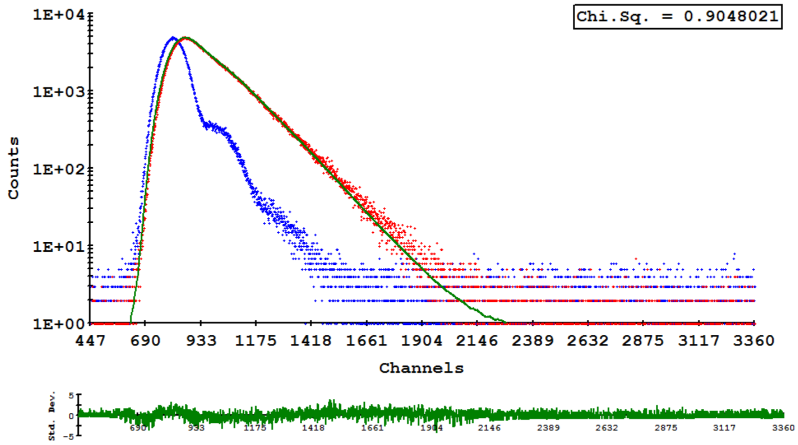
Chi.Sq. = 0.9048021
1E+04
1E+03
1E+02
1E+01
1E+00
Counts
447 690 933 1175 1418 1661 1904 2146 2389 2632 2875 3117 3360
Channels
Std. Dev.

4c

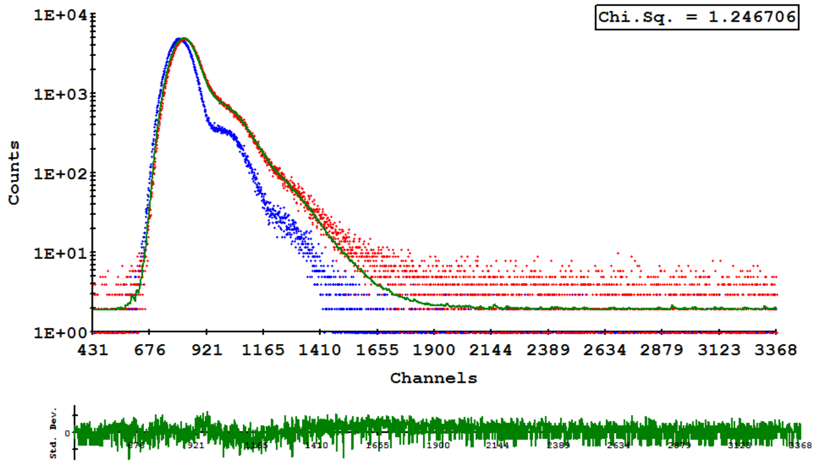
Chi.Sq. = 1.246706
1E+04
1E+03
1E+02
1E+01
1E+00
Counts
431 676 921 1165 1410 1655 1900 2144 2389 2634 2879 3123 3368
Channels
Std. Dev.

<table>
<tr><td>4d</td><td>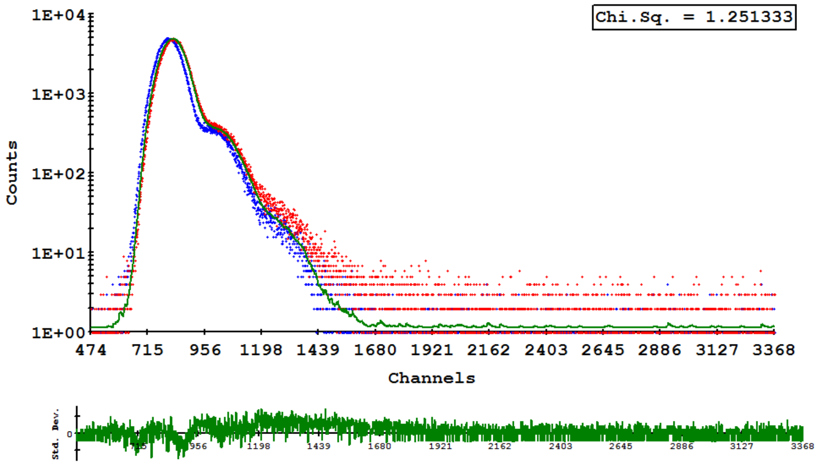
</td></tr>
<tr><td>4e</td><td>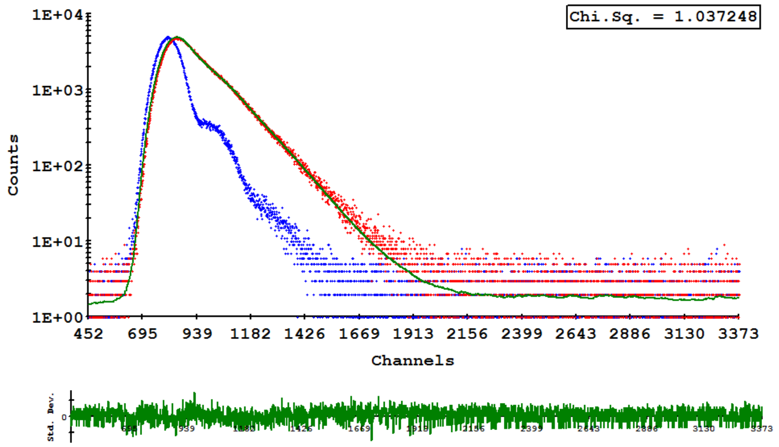
</td></tr>
<tr><td>5a</td><td>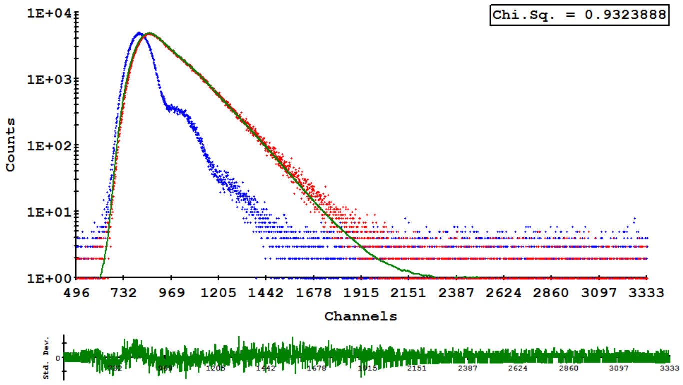
</td></tr>
</table>

**5b**	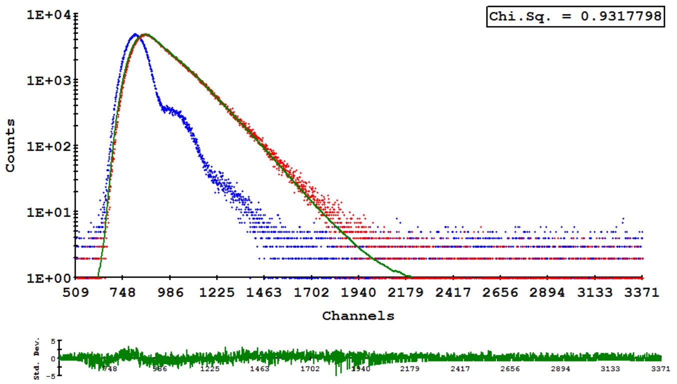Chi.Sq. = 0.9317798<br>Counts<br>Channels<br>Std. Dev.
**5c**	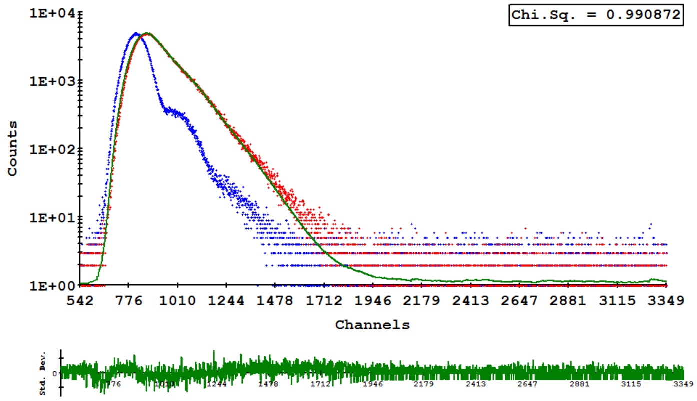Chi.Sq. = 0.990872<br>Counts<br>Channels<br>Std. Dev.
**5d**	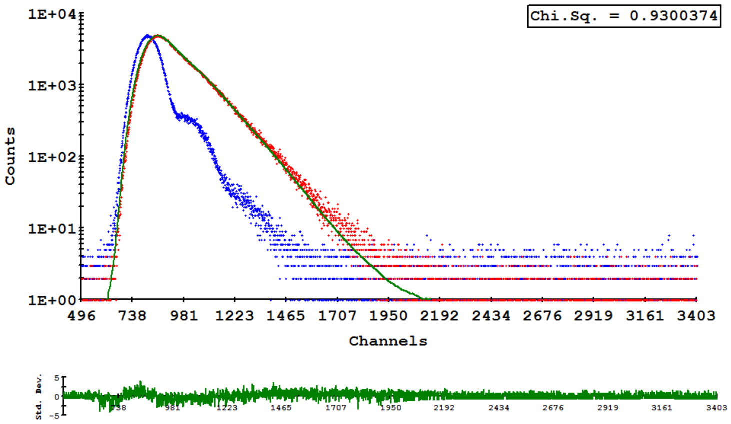Chi.Sq. = 0.9300374<br>Counts<br>Channels<br>Std. Dev.

5e

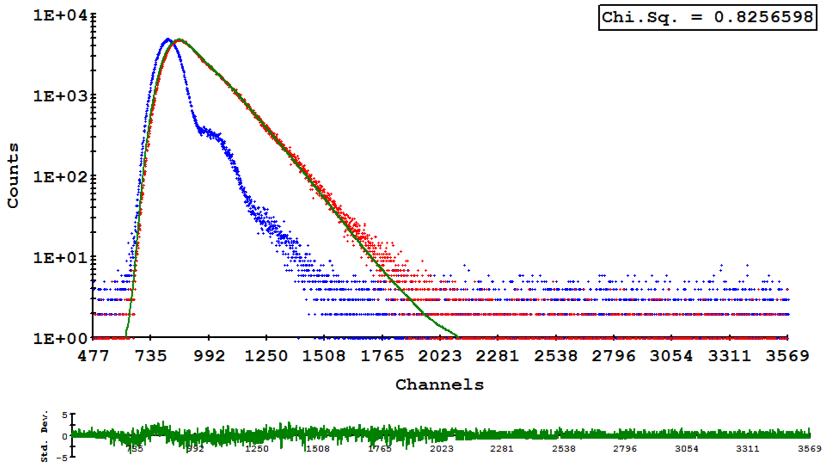

5f

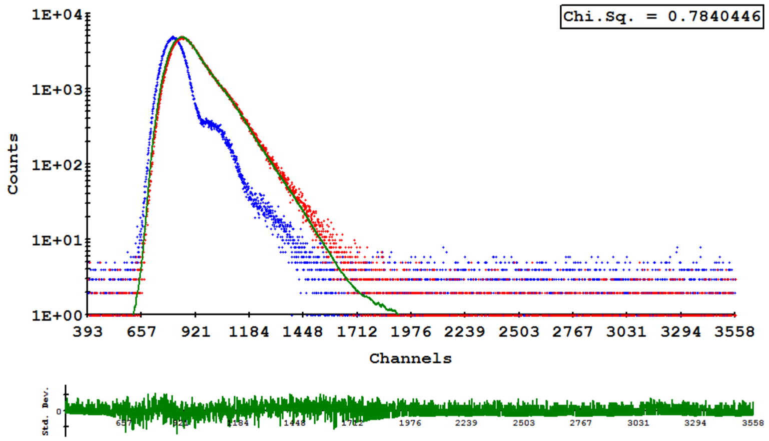

Figure S21. Time-resolved fluorescence lifetime decay profiles of **4a-f** and **5a-f** in toluene, instrumental response function (IRF, blue).

5. Solvatochromic study for compounds 4 and 5

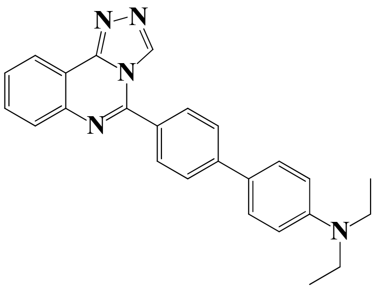

4a

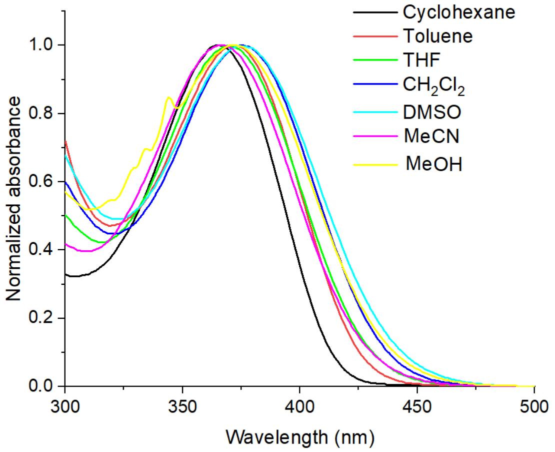

a

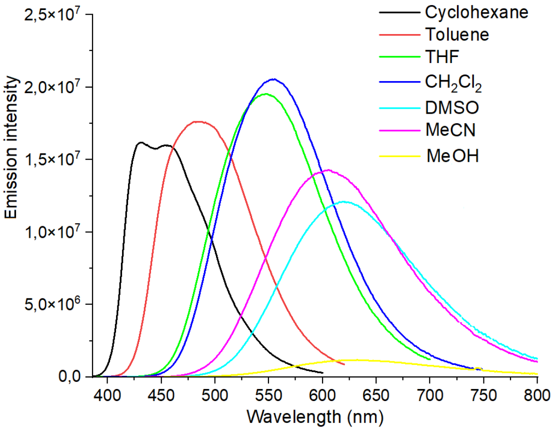

b

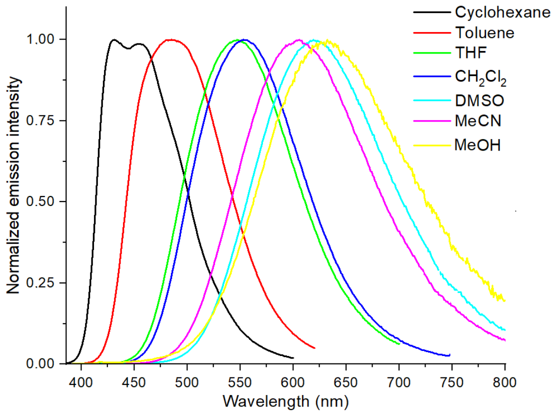

c

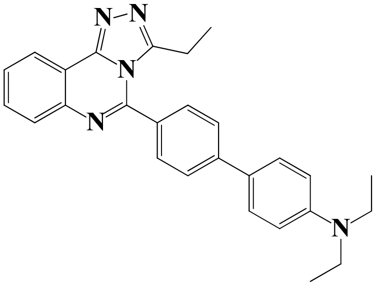

4d

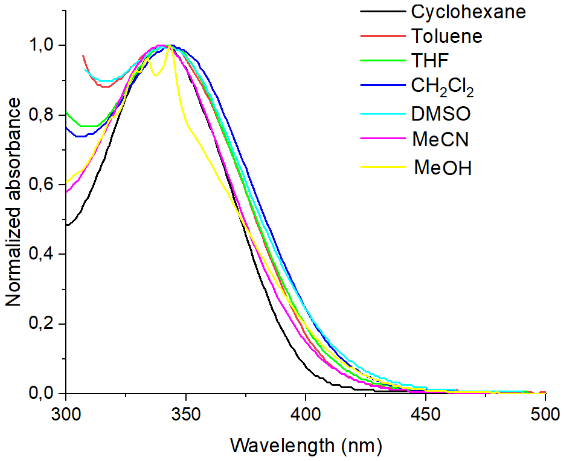

a

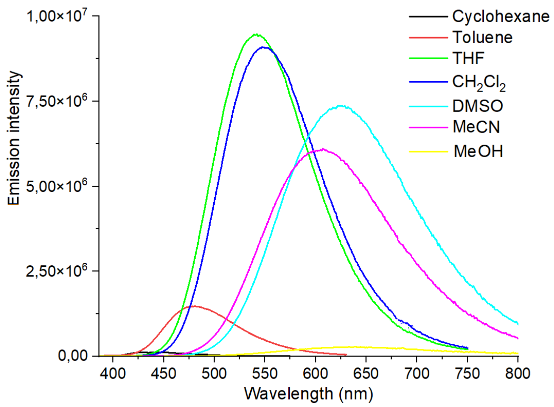
Emission intensity
Wavelength (nm)
Cyclohexane
Toluene
THF
CH_2Cl_2
DMSO
MeCN
MeOH

b

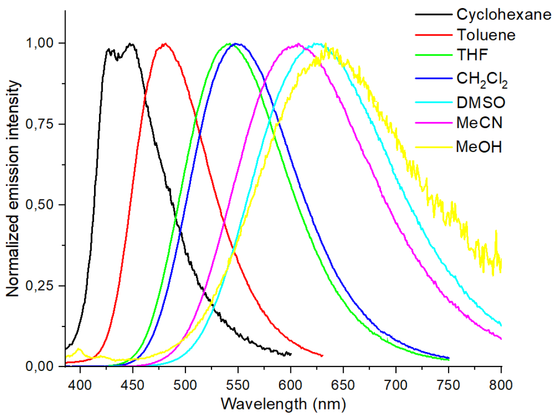
Normalized emission intensity
Wavelength (nm)
Cyclohexane
Toluene
THF
CH_2Cl_2
DMSO
MeCN
MeOH

c

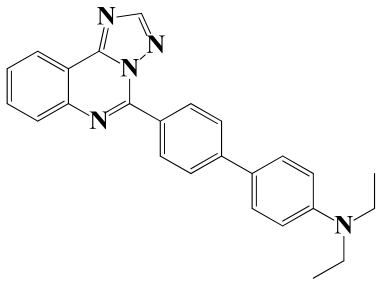

5a

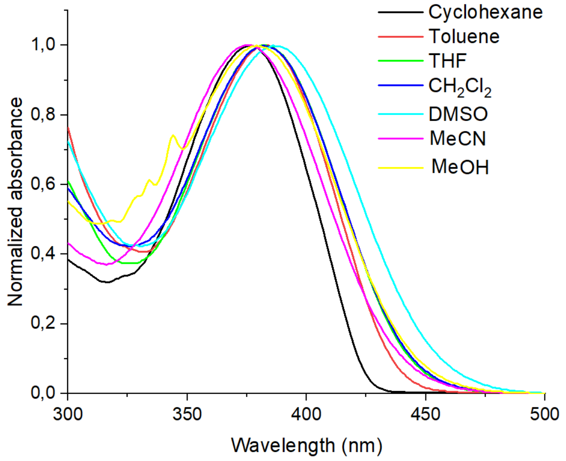
Normalized absorbance
Wavelength (nm)
Cyclohexane
Toluene
THF
CH_2Cl_2
DMSO
MeCN
MeOH

a

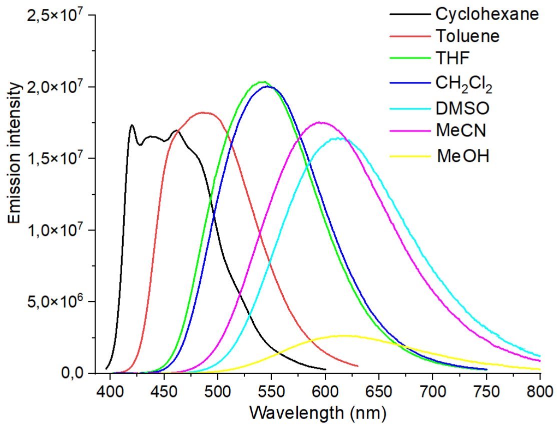
Emission intensity
Wavelength (nm)
Cyclohexane
Toluene
THF
CH_2Cl_2
DMSO
MeCN
MeOH

b

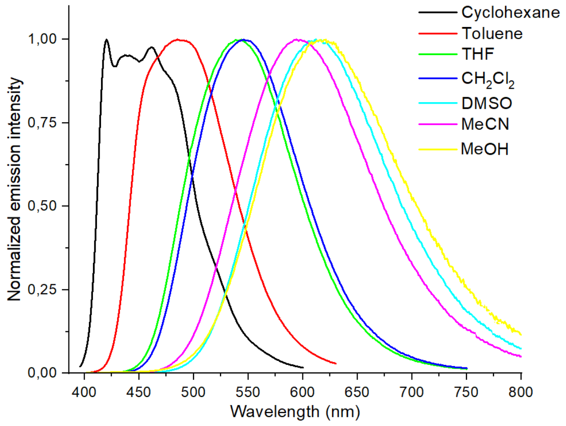
Normalized emission intensity
Wavelength (nm)
Cyclohexane
Toluene
THF
CH_2Cl_2
DMSO
MeCN
MeOH

c

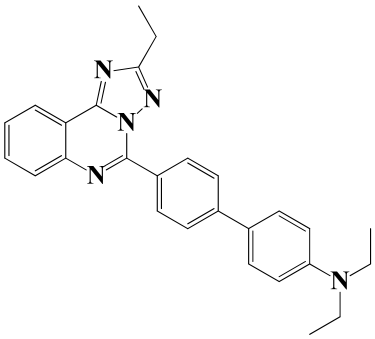

5d

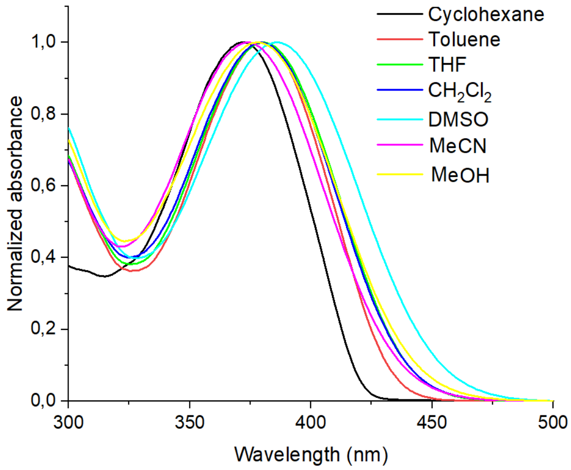
Cyclohexane
Toluene
THF
CH_2Cl_2
DMSO
MeCN
MeOH
Normalized absorbance
0,0
0,2
0,4
0,6
0,8
1,0
300
350
400
450
500
Wavelength (nm)

a

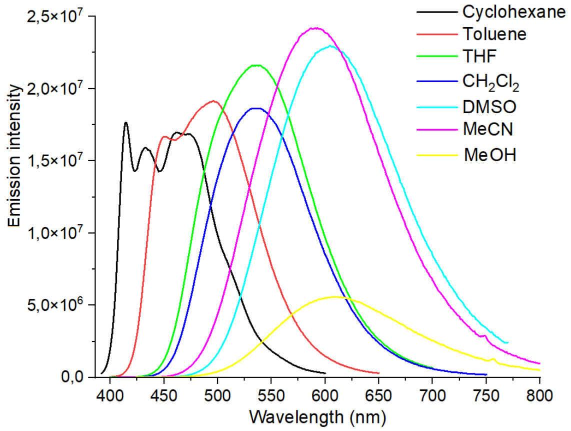
Cyclohexane
Toluene
THF
CH_2Cl_2
DMSO
MeCN
MeOH
Emission intensity
0,0
$5,0\times10^6$
$1,0\times10^7$
$1,5\times10^7$
$2,0\times10^7$
$2,5\times10^7$
400
450
500
550
600
650
700
750
800
Wavelength (nm)

b

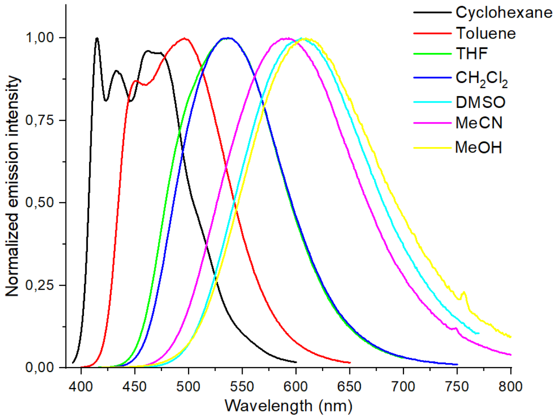
Cyclohexane
Toluene
THF
CH_2Cl_2
DMSO
MeCN
MeOH
Normalized emission intensity
0,00
0,25
0,50
0,75
1,00
400
450
500
550
600
650
700
750
800
Wavelength (nm)

c

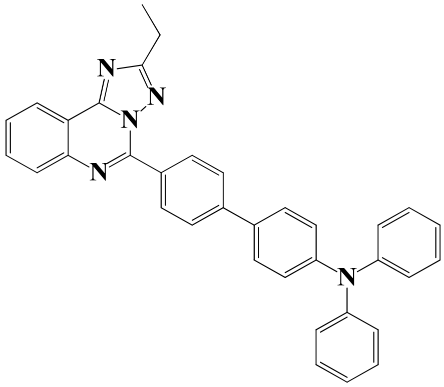

5e

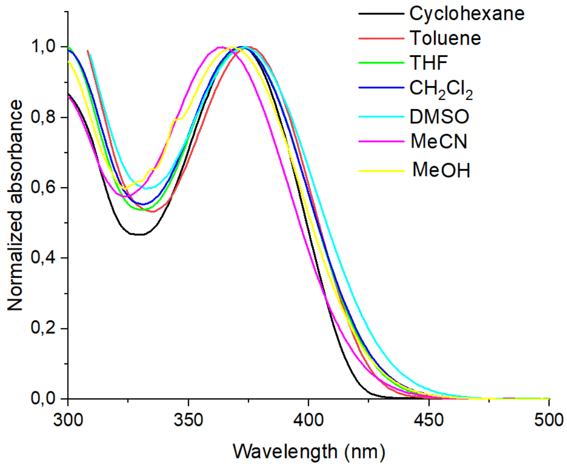
Cyclohexane
Toluene
THF
CH_2Cl_2
DMSO
MeCN
MeOH
Normalized absorbance
0,0
0,2
0,4
0,6
0,8
1,0
300
350
400
450
500
Wavelength (nm)

a

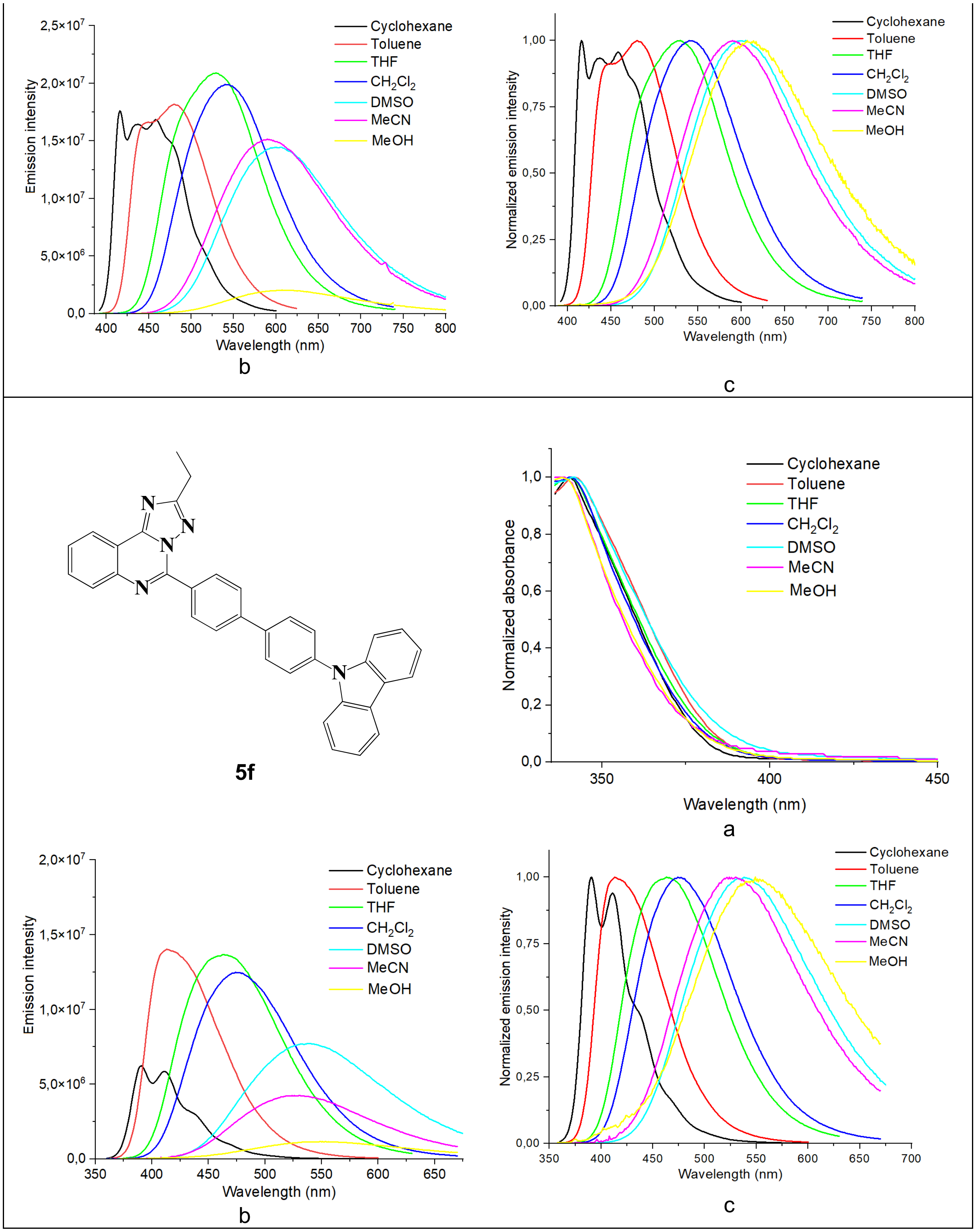

Figure S22. Normalized absorption (a), emission (b) and normalized emission (c) spectra of compounds **4a, 4d, 5a, 5d-f** in different solvents.

Table S10. Orientation polarizability for solvents (Δf), absorption and emission maxima ($λ_{abs}$, $λ_{em}$, nm) and Stokes shift (nm, cm^{-1}) of **4a** in different solvents.

Solvent	Δf	$λ_{abs}$, nm	$λ_{em}$, nm	Stokes shift, nm	Stokes shift, cm^{-1}
Cyclohexane	-0,003	366	432, **455**	89	5344
Toluene	0,013	371	476	105	5946
THF	0,208	369	548	179	8852
CH_2Cl_2	0,219	376	555	179	8578
DMSO	0,263	374	623	249	10687
MeCN	0,307	366	605	239	10793
MeOH	0,308	372	635	263	11134

Table S11. Orientation polarizability for solvents (Δf), absorption and emission maxima ($λ_{abs}$, $λ_{em}$, nm) and Stokes shift (nm, cm^{-1}) of **4d** in different solvents.

Solvent	Δf	$λ_{abs}$, nm	$λ_{em}$, nm	Stokes shift, nm	Stokes shift, cm^{-1}
Cyclohexane	-0,003	340	420, 447	107	7040
Toluene	0,013	343	481	138	8364
THF	0,208	343	541	198	10670
CH_2Cl_2	0,219	344	550	206	10888
DMSO	0,263	340	626	286	13437
MeCN	0,307	340	608	268	12964
MeOH	0,308	343	640	297	13530

Table S12. Orientation polarizability for solvents (Δf), absorption and emission maxima ($λ_{abs}$, $λ_{em}$, nm) and Stokes shift (nm, cm^{-1}) of **5a** in different solvents.

Solvent	Δf	$λ_{abs}$, nm	λem, nm	Stokes shift, nm	Stokes shift, cm^{-1}
Cyclohexane	-0,003	376	420, 440, **462**	86	4951
Toluene	0,013	383	479	96	5233
THF	0,208	382	543	161	7762
CH_2Cl_2	0,219	382	547	165	7896
DMSO	0,263	386	614	228	9620
MeCN	0,307	375	598	223	9944
MeOH	0,308	379	620	241	10256

Table S13. Orientation polarizability for solvents (Δf), absorption and emission maxima ($λ_{abs}$, $λ_{em}$, nm) and Stokes shift (nm, cm^{-1}) of **5d** in different solvents.

Solvent	Δf	$λ_{abs}$, nm	$λ_{em}$, nm	Stokes shift, nm	Stokes shift, cm^{-1}
Cyclohexane	-0,003	372	415, 434, **469**	97	5560
Toluene	0,013	380	486	106	5740
THF	0,208	380	537	157	7694
CH_2Cl_2	0,219	380	537	157	7694
DMSO	0,263	386	606	220	9405
MeCN	0,307	374	579	205	9467
MeOH	0,308	378	612	234	10115

Table S14. Orientation polarizability for solvents (Δf), absorption and emission maxima (λ_{abs}, λ_{em}, nm) and Stokes shift (nm, cm^{-1}) of **5e** in different solvents.

Solvent	Δf	λ_{abs}, nm	λem, nm	Stokes shift, nm	Stokes shift, cm^{-1}
Cyclohexane	-0,003	372	416, 437, **459**	87	5095
Toluene	0,013	375	465	190	5161
THF	0,208	372	529	157	7978
CH_2Cl_2	0,219	372	543	171	8466
DMSO	0,263	374	604	230	10182
MeCN	0,307	363	593	230	10685
MeOH	0,308	369	613	244	10787

Table S15. Orientation polarizability for solvents (Δf), absorption and emission maxima (λ_{abs}, λ_{em}, nm) and Stokes shift (nm, cm^{-1}) of **5f** in different solvents.

Solvent	Δf	λ_{abs}, nm	λ_{em}, nm	Stokes shift, nm	Stokes shift, cm^{-1}
Cyclohexane	-0,003	340	391, **411**	71	5081
Toluene	0,013	342	420	78	5430
THF	0,208	341	464	123	7774
CH_2Cl_2	0,219	340	477	137	8447
DMSO	0,263	341	540	199	10807
MeCN	0,307	339	530	191	10631
MeOH	0,308	338	553	215	11503

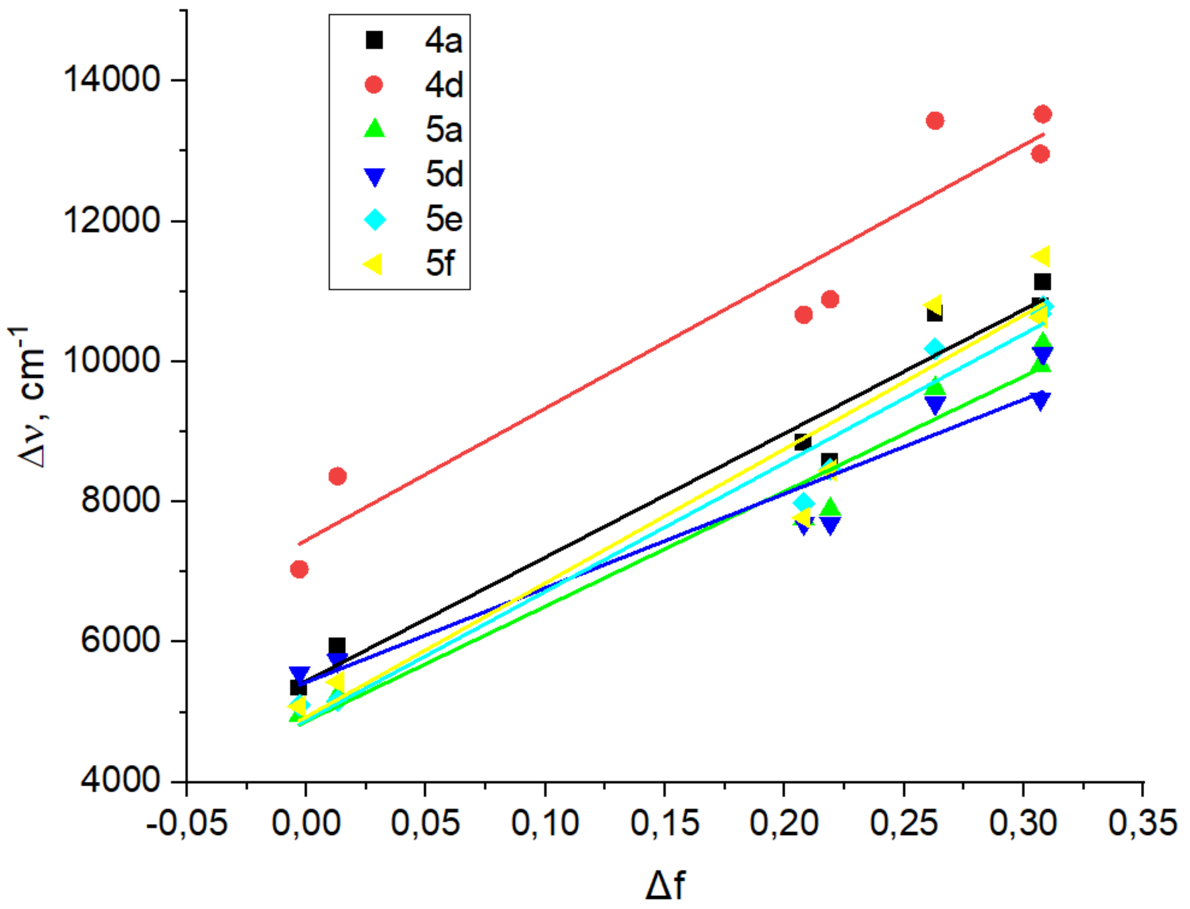

Figure S23. Lippert-Mataga plot of fluorophores **4a, 4d, 5a, 5d, 5e** and **5f** in cyclohexane, toluene, THF, CH_2Cl_2, DMSO, MeCN and MeOH.

6. Cyclic voltammograms for compounds 4a-f and 5a-f

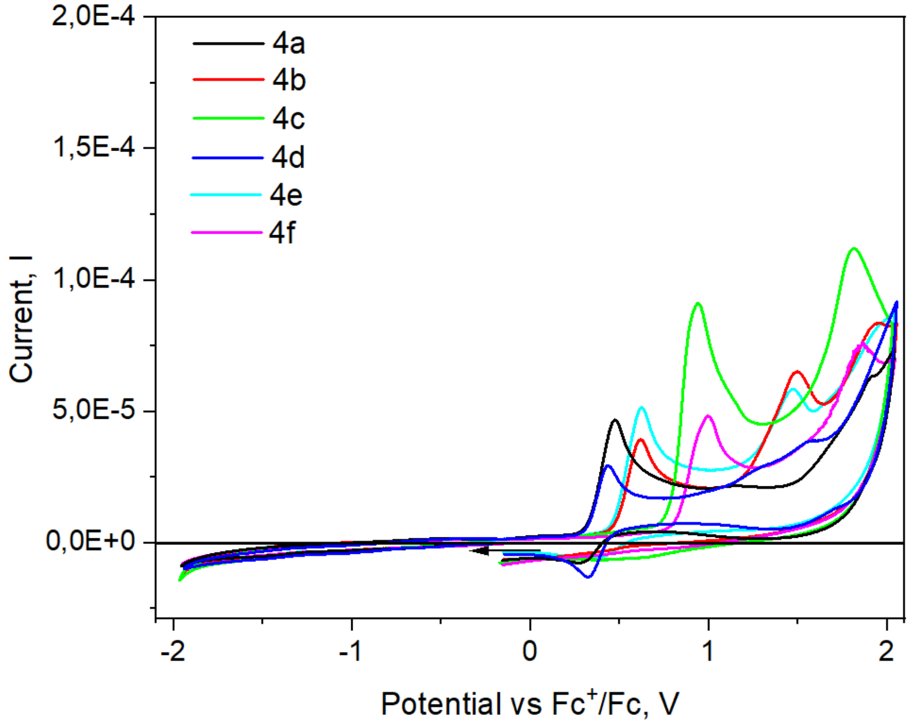

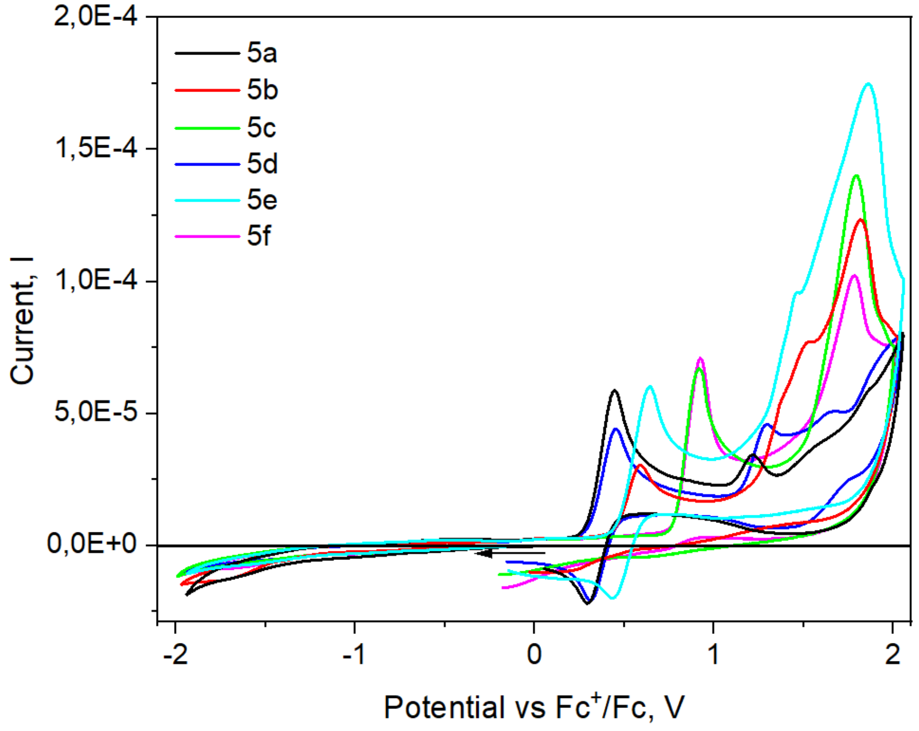

Figure S24. General view of voltammograms for **4a-f** and **5a-f** (0.1 M Bu_4NPF_6 in CH_2Cl_2 as supporting electrolyte, scan rate 100 mV/s, platinum disk working electrode, glassy carbon disk counter electrode, Ag/$AgNO_3$ pseudoreference electrode, Fc^+/Fc couple as an internal standard).

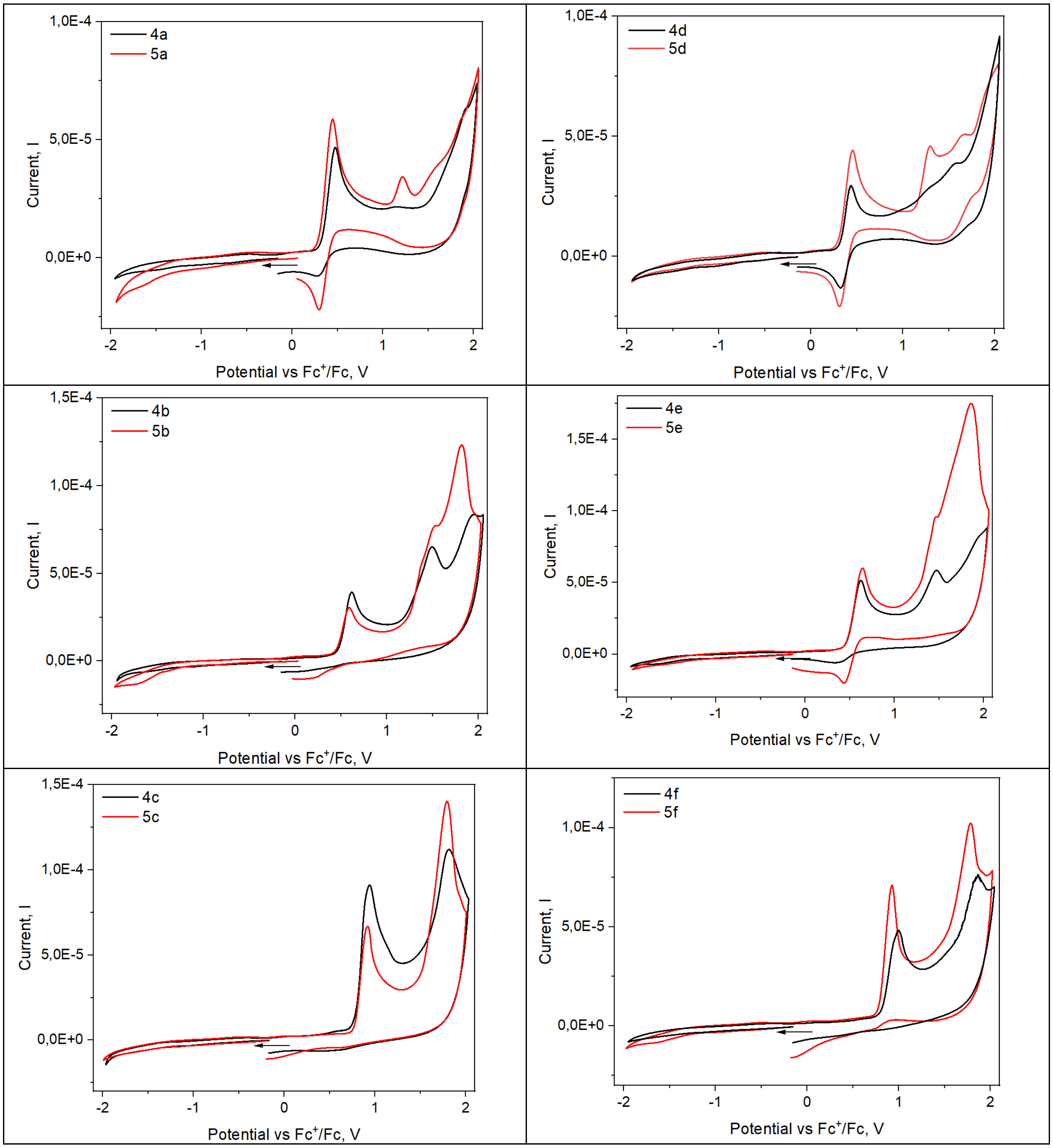

Figure S25. Comparison of voltammograms for each pair of isomers.

7. Theoretical calculations

Table S16. The electronic distribution in HOMO/LUMO of **4a-f** and **5a-f** calculated for gas phase.

Compound	HOMO, eV	LUMO, eV	Eg
**4a**	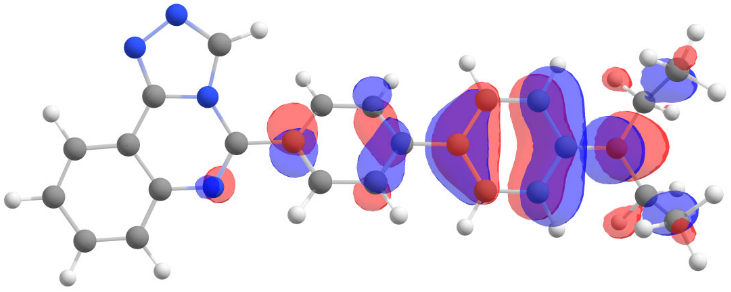 -5.61	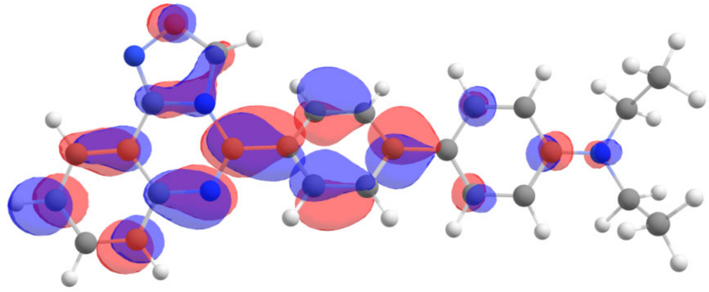 -1.87	-3.74
**4b**	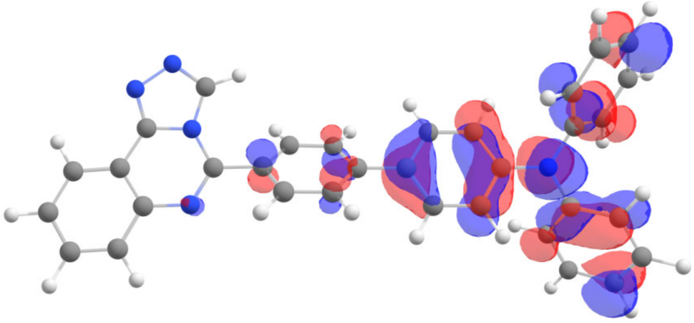 -5.60	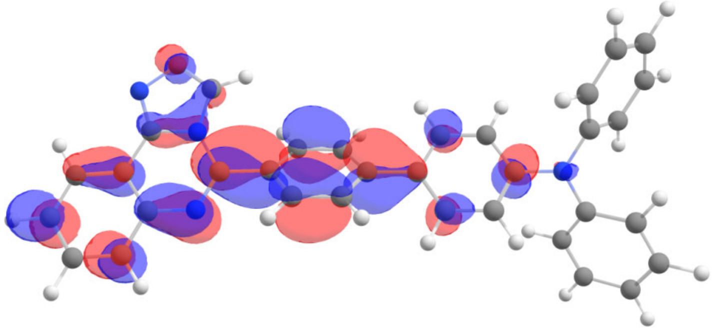 -2.02	-3.58

**4c**	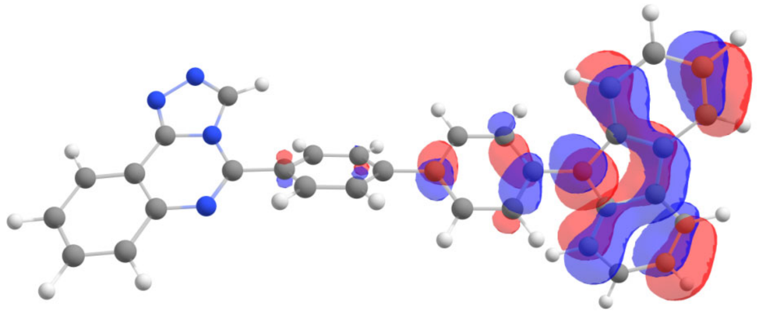 -5.98	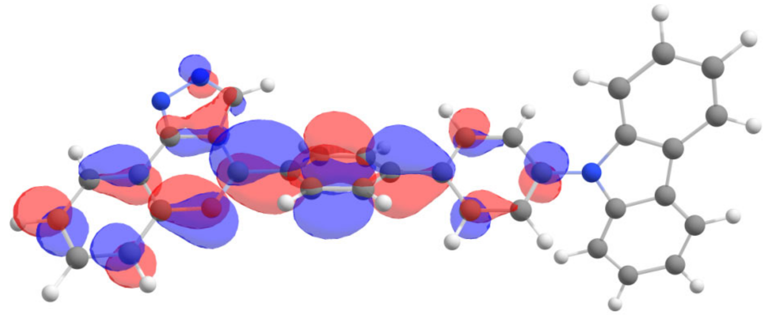 -2.21	-3.77
**4d**	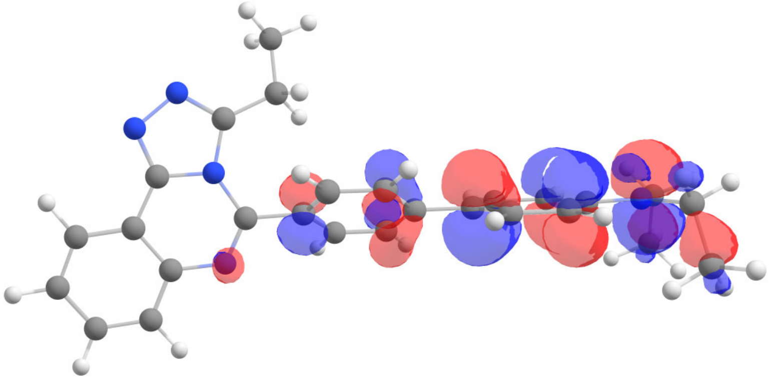 -5.58	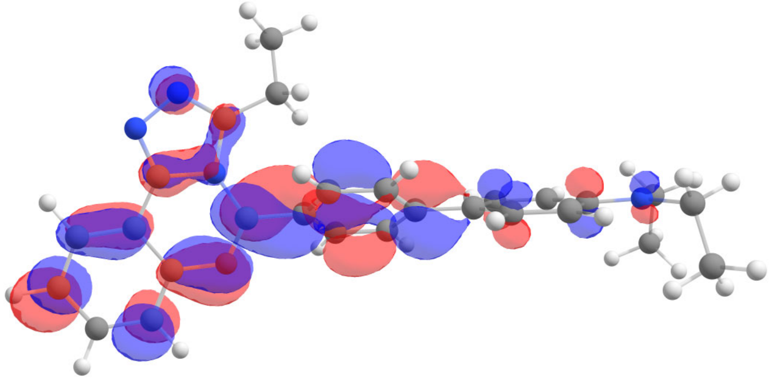 -1.64	-3.94
**4e**	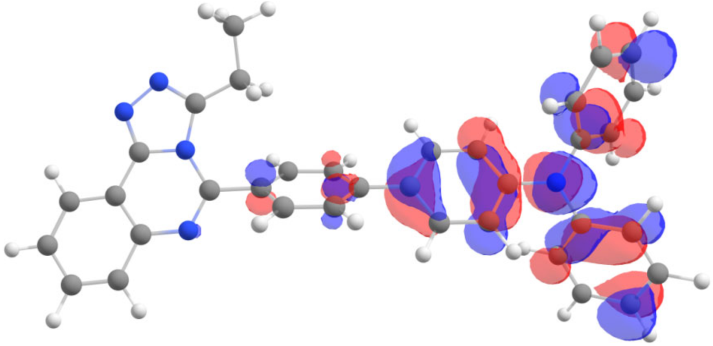 -5.58	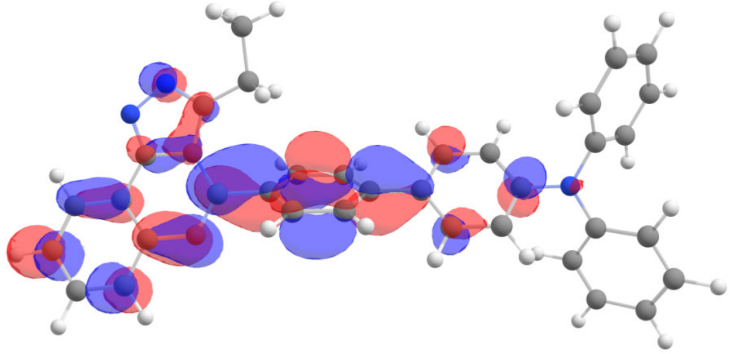 -1.82	-3.76

**4f**	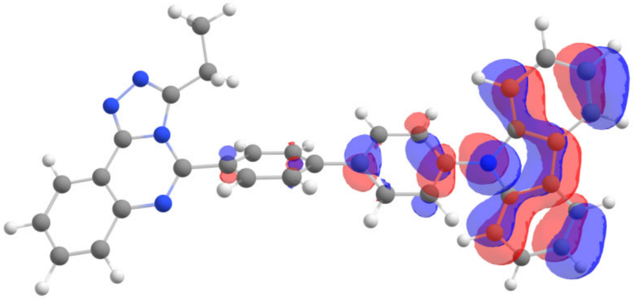<br>-5.96	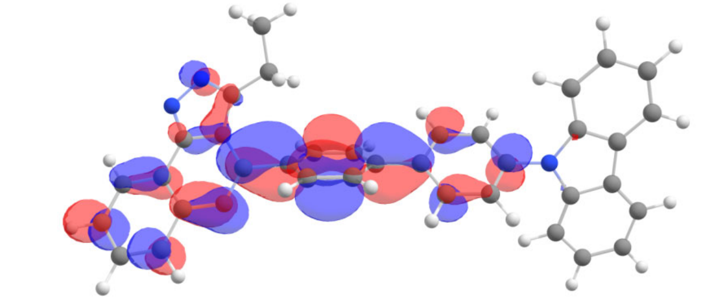<br>-2.01	-3.95
**5a**	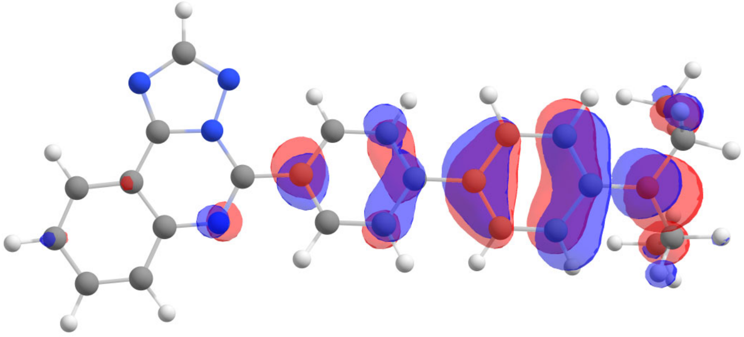<br>-5.44	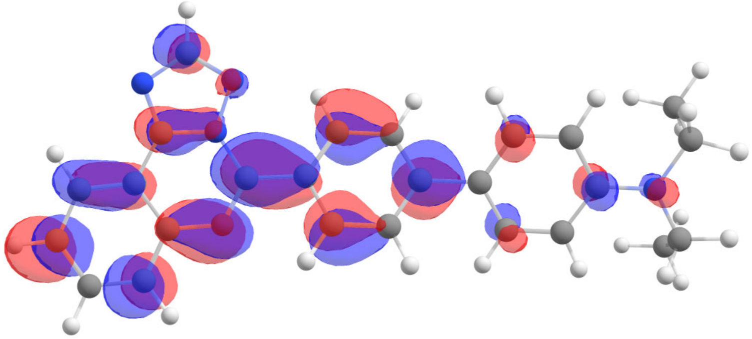<br>-1.84	-3.6
**5b**	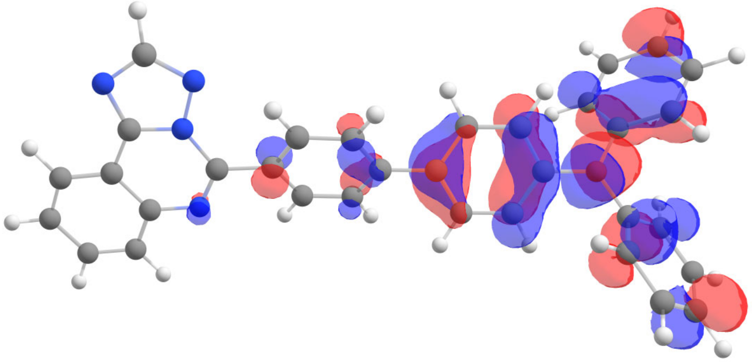<br>-5.47	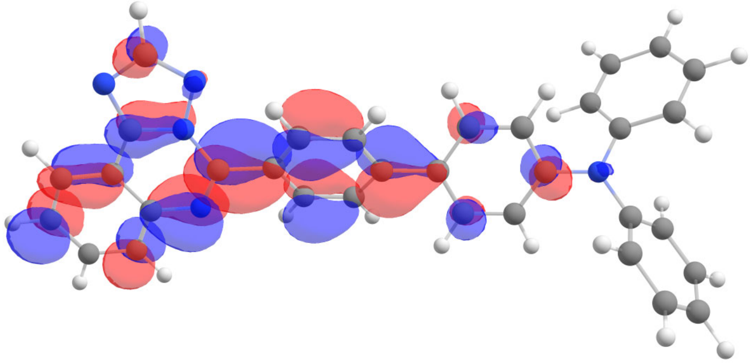<br>-2.00	-3.47

**5c**	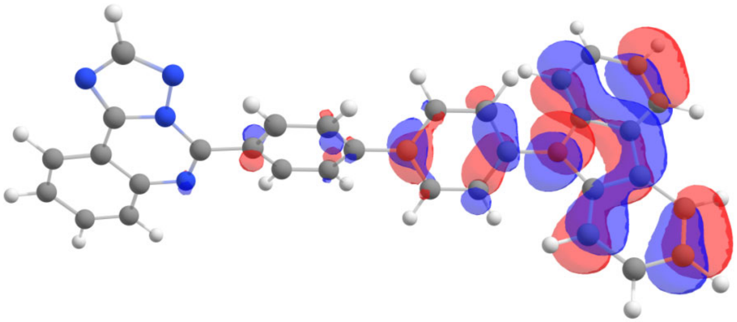 -5.86	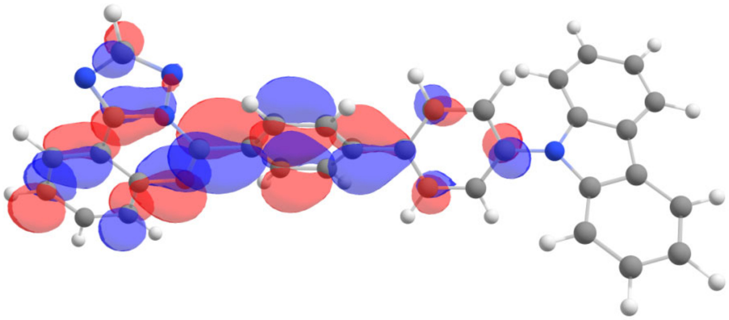 -2.15	-3.71
**5d**	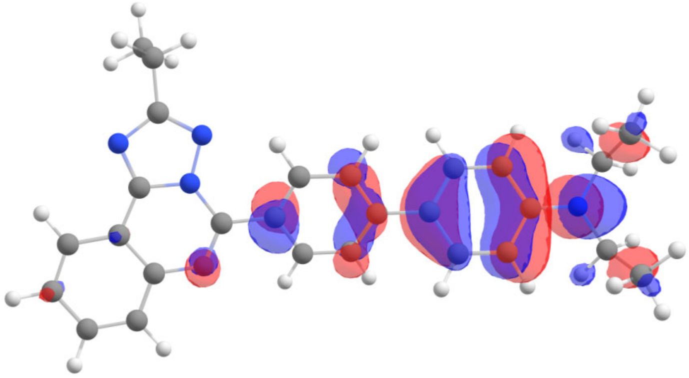 -5.41	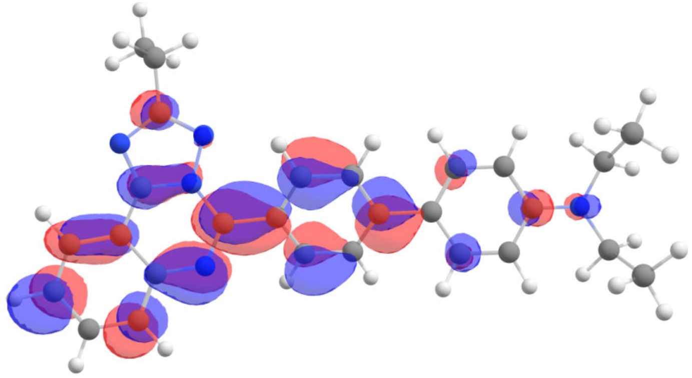 -1.75	-3.66
**5e**	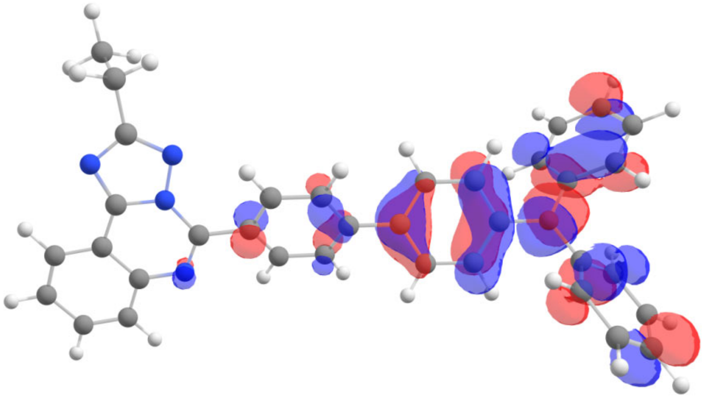	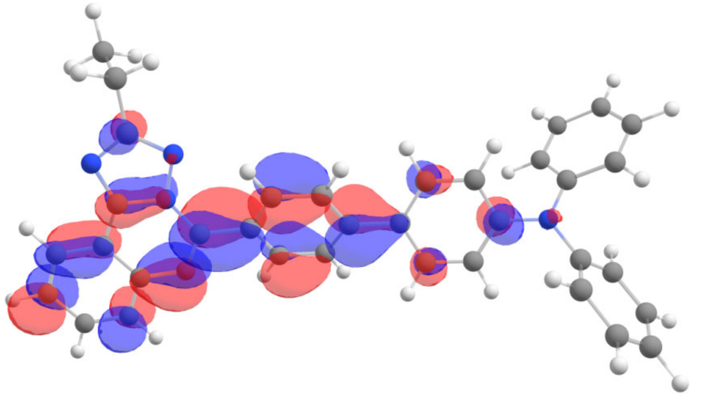	-3.53

	-5.45	-1.92	
**5f**	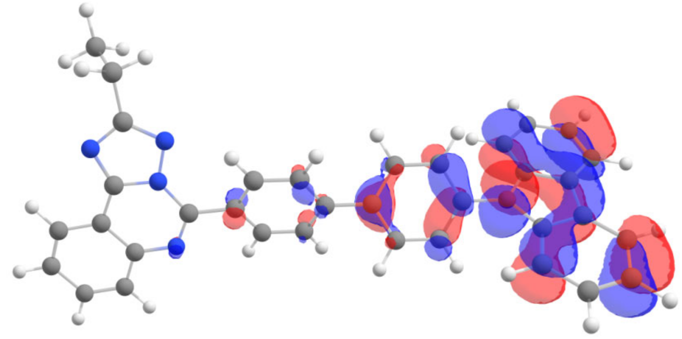 -5.85	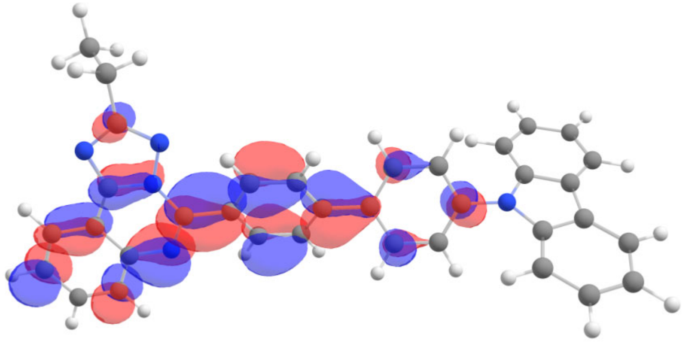 -2.07	-3.78

Table S17. The optimized geometries of **4a-f** and **5a-f** in gas phase, toluene and MeCN

#	S_0	S_1
**4a**	Gas phase 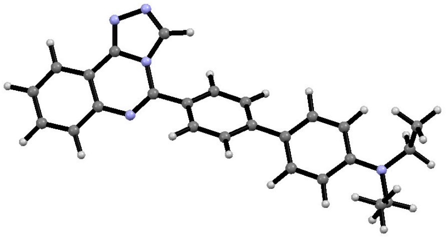	Gas phase 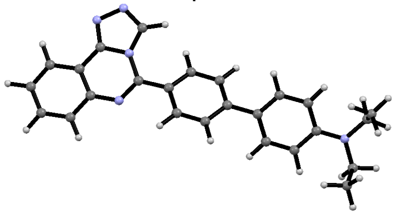
		Toluene 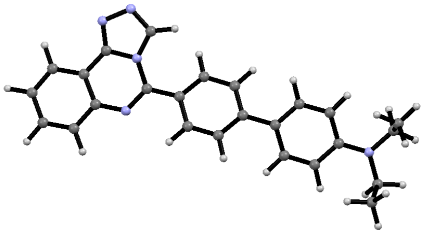
		MeCN 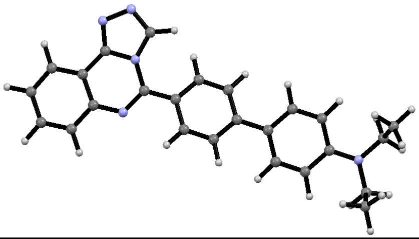
**4b**	Gas phase 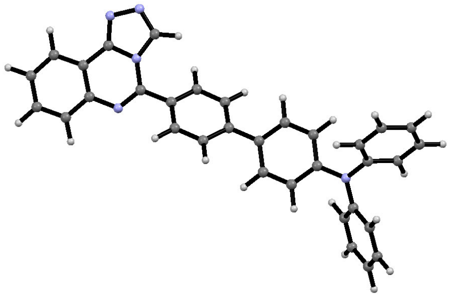	Gas phase 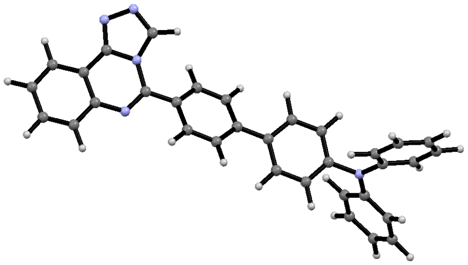
		Toluene 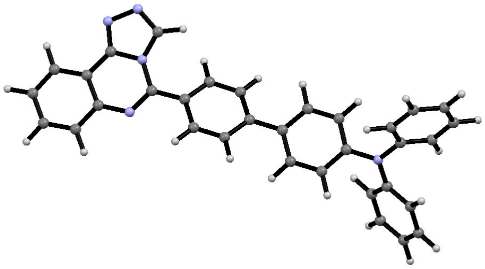
		MeCN 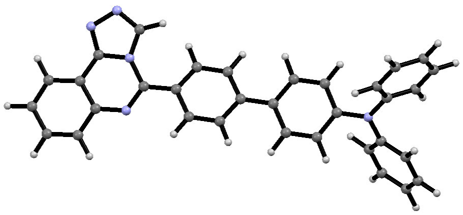
**4c**	Gas phase	Gas phase

<table>
<tr><td rowspan="3"></td><td rowspan="3">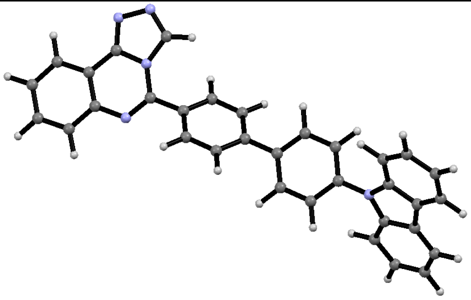</td><td>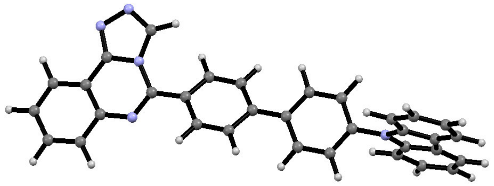</td></tr>
<tr><td>Toluene
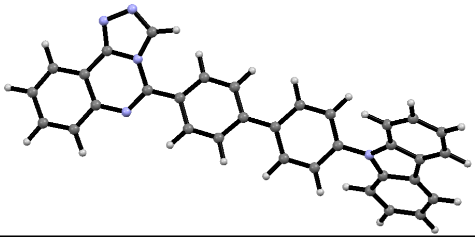</td></tr>
<tr><td>MeCN
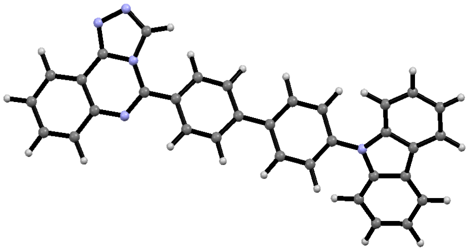</td></tr>
<tr><td rowspan="3">4d</td><td rowspan="3">Gas phase
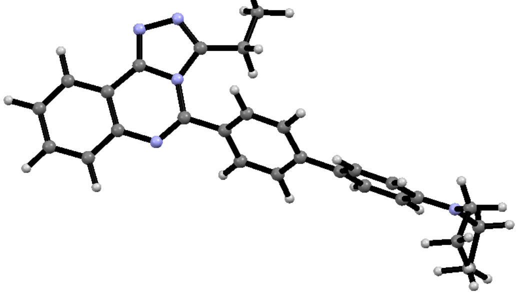</td><td>Gas phase
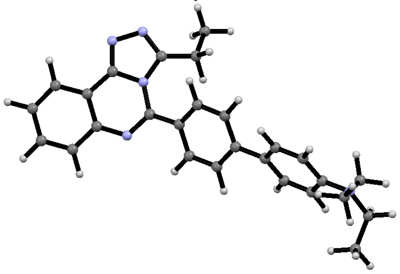</td></tr>
<tr><td>Toluene
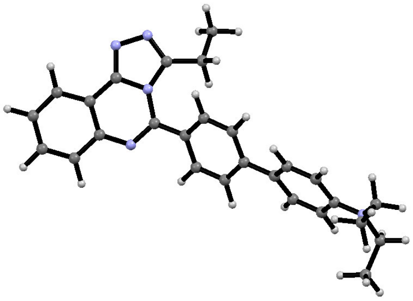</td></tr>
<tr><td>MeCN
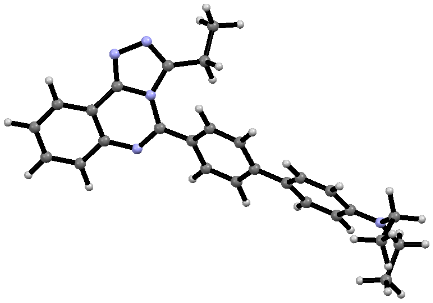</td></tr>
</table>

<table>
<tr><td rowspan="3">4e</td><td rowspan="3">Gas phase
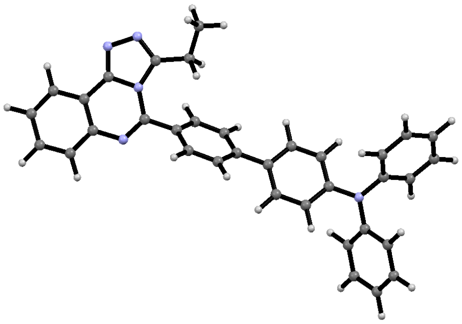</td><td>Gas phase
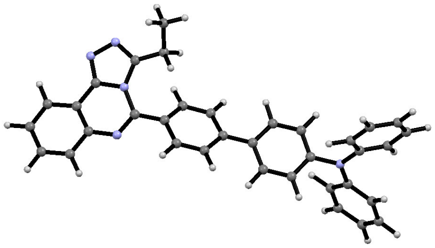</td></tr>
<tr><td>Toluene
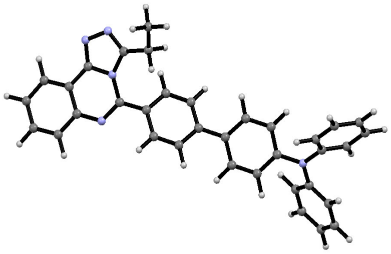</td></tr>
<tr><td>MeCN
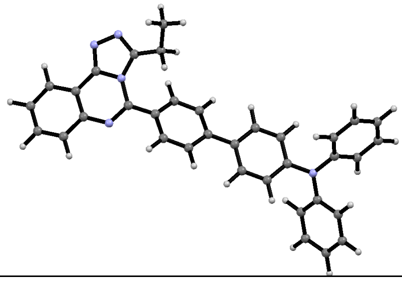</td></tr>
<tr><td rowspan="3">4f</td><td rowspan="3">Gas phase
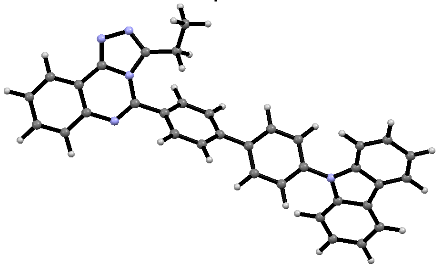</td><td>Gas phase
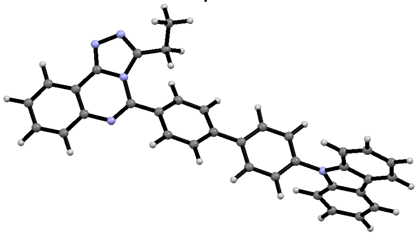</td></tr>
<tr><td>Toluene
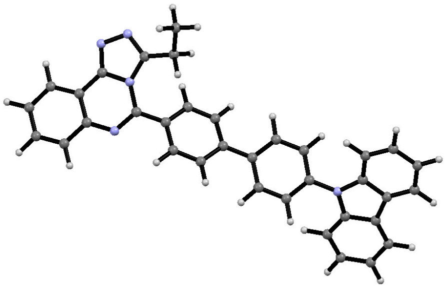</td></tr>
<tr><td>MeCN
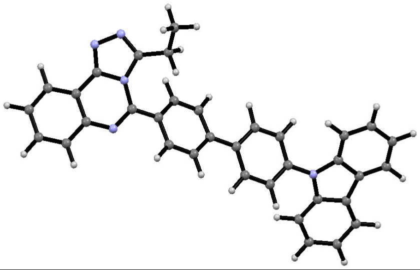</td></tr>
</table>

**5a**	Gas phase 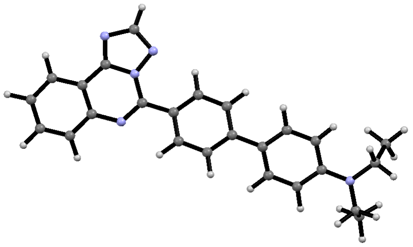	Gas phase 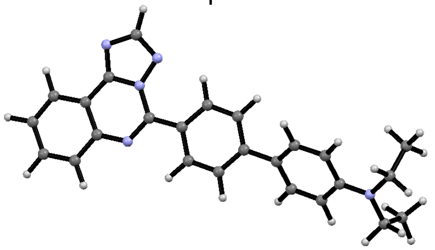
		Toluene 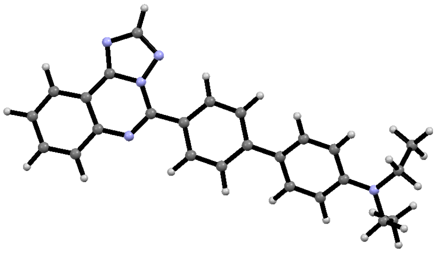
		MeCN 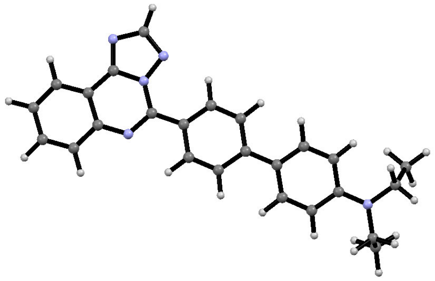
**5b**	Gas phase 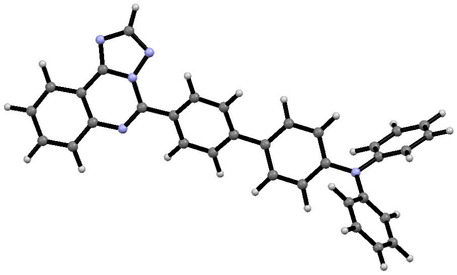	Gas phase 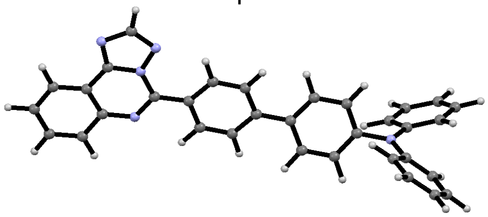
		Toluene 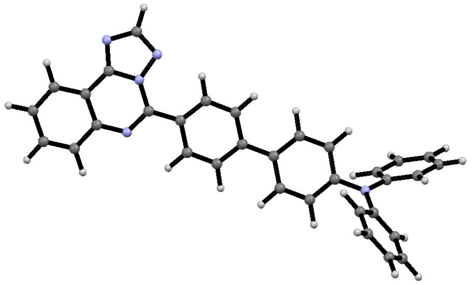
		MeCN 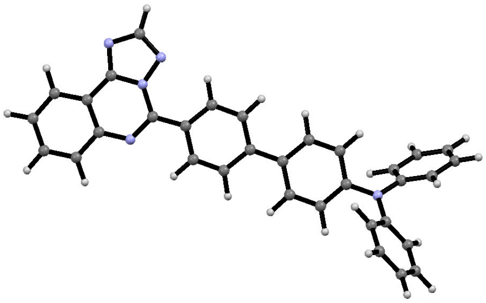

<table>
<tr><td rowspan="3">5c</td><td rowspan="3">Gas phase
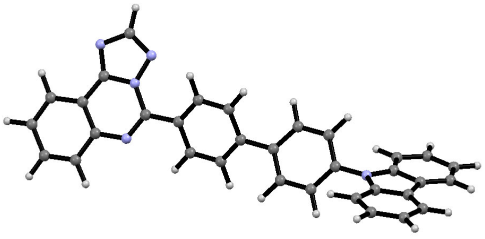</td><td>Gas phase
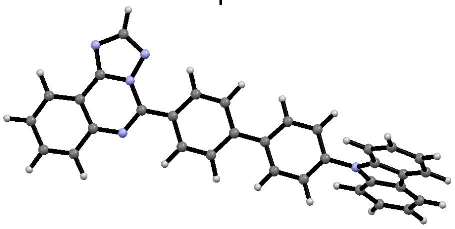</td></tr>
<tr><td>Toluene
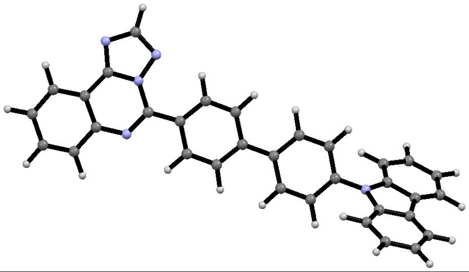</td></tr>
<tr><td>MeCN
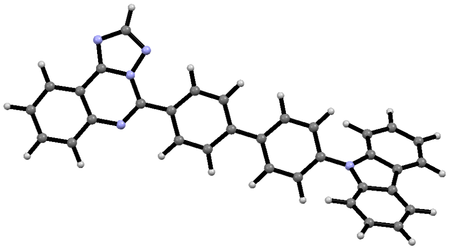</td></tr>
<tr><td rowspan="3">5d</td><td rowspan="3">Gas phase
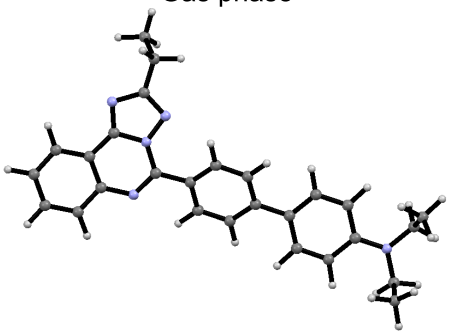</td><td>Gas phase
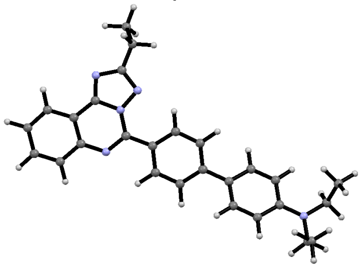</td></tr>
<tr><td>Toluene
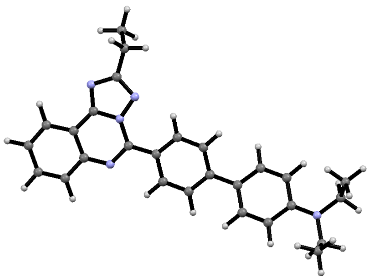</td></tr>
<tr><td>MeCN
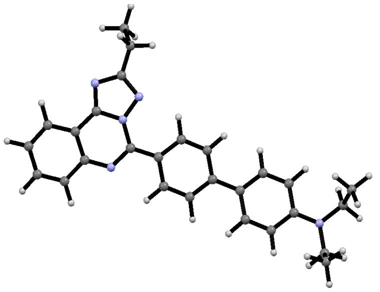</td></tr>
</table>

<table>
<tr><td rowspan="3">5e</td><td rowspan="3">Gas phase
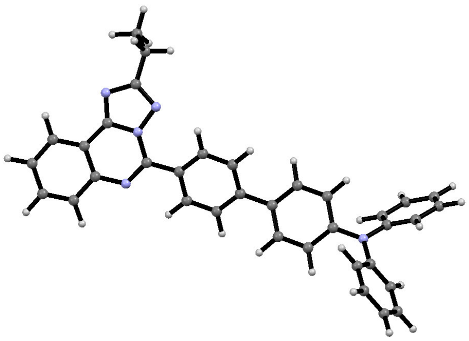</td><td>Gas phase
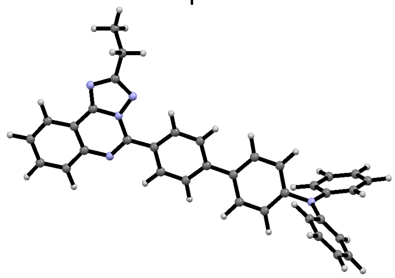</td></tr>
<tr><td>Toluene
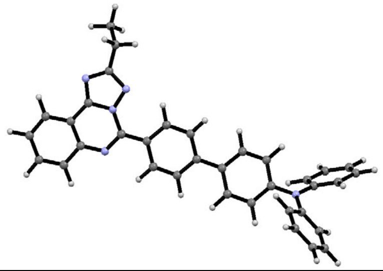</td></tr>
<tr><td>MeCN
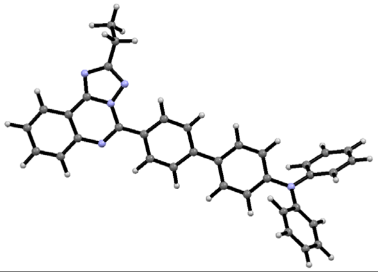</td></tr>
<tr><td rowspan="3">5f</td><td rowspan="3">Gas phase
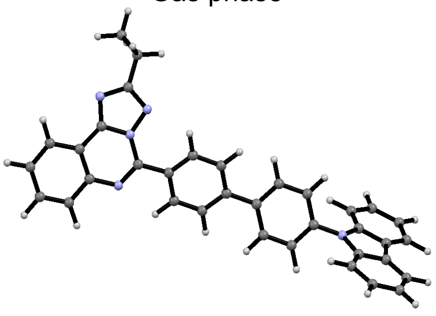</td><td>Gas phase
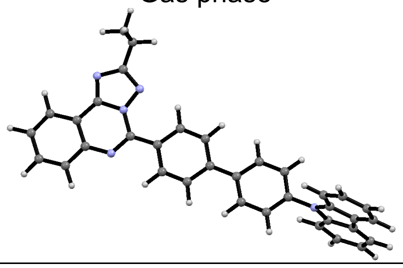</td></tr>
<tr><td>Toluene
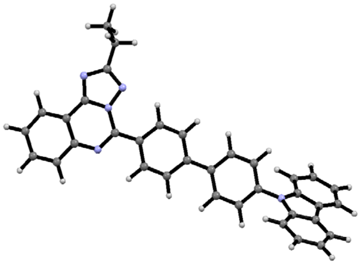</td></tr>
<tr><td>MeCN</td></tr>
</table>

		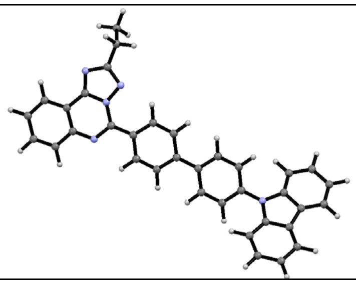

Table S18. Selected dihedral angles (α) and bond lengths (L) in the ground and excited states of **4a-f** and **5a-f** in toluene and MeCN.

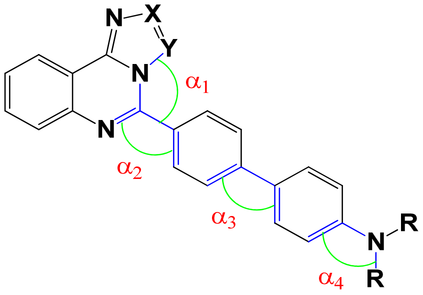

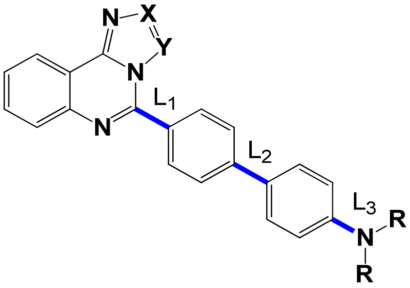

	Phase/Solvent	S	α_1, °	α_2, °	α_3, °	α_4, °	L_1, Å	L_2, Å	L_3, Å
**4a**	Gas	S_0	-6.9	-38.4	31.2	-6.5	1.467	1.466	1.371
		S_1	-13.1	-19.0	44.5	-5.7	1.439	1.470	1.361
	Toluene	S_1	-13.4	-20.4	29.0	-4.8	1.436	1.459	1.356
	MeCN	S_1	-14.0	-19.8	20.6	-3.8	1.430	1.450	1.349
**4b**	Gas	S_0	-6.1	-38.8	31.4	33.0	1.468	1.470	1.399
		S_1	-12.3	-19.6	19.6	71.9	1.432	1.447	1.440
	Toluene	S_1	-12.6	-19.9	20.8	45.8	1.431	1.451	1.410
	MeCN	S_1	-13.3	-18.8	18.7	38.5	1.427	1.445	1.392
**4c**	Gas	S_0	-6.9	-36.1	36.9	54.1	1.470	1.472	1.405
		S_1	-12.1	-19.4	15.3	-87.0	1.431	1.440	1.433
	Toluene	S_1	-13.4	-19.4	16.8	50.8	1.428	1.443	1.404
	MeCN	S_1	-14.2	-18.4	10.1	43.3	1.423	1.436	1.387
**4d**	Gas	S_0	10.0	54.8	32.8	-5.7	1.472	1.469	1.372
		S_1	31.3	28.9	33.1	-3.9	1.439	1.463	1.363
	Toluene	S_1	31.1	30.2	25.8	-5.0	1.436	1.457	1.356
	MeCN	S_1	31.7	30.2	19.2	-4.6	1.430	1.450	1.349
**4e**	Gas	S_0	-11.8	-55.2	34.4	35.1	1.473	1.470	1.401
		S_1	-26.5	-27.9	19.3	68.8	1.433	1.446	1.437
	Toluene	S_1	-28.16	-27.9	19.0	44.5	1.431	1.449	1.407
	MeCN	S_1	-29.8	-27.2	14.5	36.6	1.426	1.444	1.389
**4f**	Gas	S_0	-14.0	-52.7	34.7	53.0	1.473	1.472	1.405
		S_1	-25.5	-28.3	13.2	86.4	1.433	1.438	1.432
	Toluene	S_1	-29.2	-26.0	16.3	48.9	1.426	1.442	1.399
	MeCN	S_1	-30.8	-25.3	12.6	43.9	1.420	1.436	1.388
**5a**	Gas	S_0	-1.4	-16.5	32.9	0.7	1.468	1.466	1.372
		S_1	0.5	-1.1	39.7	-1.0	1.448	1.468	1.361
	Toluene	S_1	-1.1	-2.4	28.2	-0.5	1.441	1.458	1.356
	MeCN	S_1	-1.4	-4.3	20.7	-0.6	1.433	1.450	1.349
**5b**	Gas	S_0	1.7	21.7	-32.1	-36.0	1.468	1.469	1.402
		S_1	-0.1	0.8	-20.8	-66.7	1.435	1.450	1.437
	Toluene	S_1	1.4	2.8	-19.1	-44.8	1.434	1.450	1.407
	MeCN	S_1	2.1	5.3	-12.7	-37.0	1.430	1.444	1.390
**5c**	Gas	S_0	1.6	19.3	-36.3	-53.1	1.470	1.471	1.405
		S_1	0.2	0.1	-15.8	86.9	1.432	1.441	1.433
	Toluene	S_1	0.7	1.8	-14.5	-49.3	1.432	1.442	1.401
	MeCN	S_1	0.4	2.0	-6.8	-44.0	1.426	1.436	1.387
**5d**	Gas	S_0	2.1	19.6	-30.7	-2.1	1.468	1.467	1.373
		S_1	0.4	2.4	-37.8	-1.5	1.446	1.466	1.362
	Toluene	S_1	1.0	4.4	-26.3	-1.1	1.439	1.457	1.356
	MeCN	S_1	0.7	3.2	-20.4	-0.4	1.432	1.449	1.349
**5e**	Gas	S_0	2.0	19.2	-32.5	-35.3	1.469	1.469	1.401
		S_1	-0.1	1.2	-19.7	-65.7	1.435	1.448	1.436
	Toluene	S_1	0.0	-0.5	-10.5	-45.4	1.434	1.448	1.408
	MeCN	S_1	1.0	2.6	-11.8	-37.1	1.430	1.443	1.390

**5f**	Gas	S_0	2.2	19.7	-34.9	-52.5	1.471	1.471	1.405
		S_1	0.1	0.7	-14.2	-87.8	1.433	1.439	1.432
	Toluene	S_1	-0.9	-0.1	-15.3	-49.5	1.431	1.441	1.400
	MeCN	S_1	-0.2	0.8	1.5	-43.3	1.427	1.434	1.387

Table S19. The electronic $S_0 \rightarrow S_n$ transition properties of compounds **4a-f** and **5a-f**.

#	State/ Energy(cm^{-1})/ Wavelength(nm)/ f_{osc}	
**4a**	1 26700.4 374.5 0.885420282<br>2 32365.1 309.0 0.051737291<br>3 33122.3 301.9 0.134253142	STATE 1: E= 0.121656 au 3.310 eV 26700.4 cm**-1 <S**2> = 0.000000<br>103a -> 104a : 0.978340 (c= 0.98911072)<br><br>STATE 2: E= 0.147466 au 4.013 eV 32365.1 cm**-1 <S**2> = 0.000000<br>101a -> 104a : 0.135764 (c= 0.36846201)<br>101a -> 105a : 0.035003 (c= -0.18709090)<br>101a -> 107a : 0.010457 (c= -0.10225798)<br>102a -> 104a : 0.637255 (c= 0.79828236)<br>102a -> 105a : 0.017330 (c= 0.13164379)<br>103a -> 105a : 0.137650 (c= -0.37101192)<br><br>STATE 3: E= 0.150916 au 4.107 eV 33122.3 cm**-1 <S**2> = 0.000000<br>102a -> 104a : 0.178986 (c= -0.42306754)<br>102a -> 105a : 0.010585 (c= 0.10288155)<br>103a -> 105a : 0.764584 (c= -0.87440508)<br>103a -> 106a : 0.021318 (c= -0.14600542)
**4b**	1 25220.2 396.5 0.803416720<br>2 30580.7 327.0 0.014881890<br>3 31880.4 313.7 0.356740104	STATE 1: E= 0.114912 au 3.127 eV 25220.2 cm**-1 <S**2> = 0.000000<br>127a -> 128a : 0.968726 (c= 0.98423883)<br>127a -> 130a : 0.010074 (c= -0.10037013)<br><br>STATE 2: E= 0.139336 au 3.792 eV 30580.7 cm**-1 <S**2> = 0.000000<br>127a -> 131a : 0.913650 (c= 0.95585060)<br>127a -> 132a : 0.014716 (c= -0.12130776)<br>127a -> 133a : 0.017828 (c= 0.13351977)<br><br>STATE 3: E= 0.145258 au 3.953 eV 31880.4 cm**-1 <S**2> = 0.000000<br>125a -> 128a : 0.093294 (c= 0.30544074)<br>125a -> 130a : 0.014512 (c= 0.12046712)<br>126a -> 128a : 0.734613 (c= 0.85709550)<br>127a -> 130a : 0.098188 (c= 0.31335007)
**4c**	1 26600.0 375.9 0.416519327<br>2 30390.1 329.1 0.000009486<br>3 31316.4 319.3 0.353213004	STATE 1: E= 0.121198 au 3.298 eV 26600.0 cm**-1 <S**2> = 0.000000<br>126a -> 127a : 0.958550 (c= -0.97905572)<br>126a -> 129a : 0.020325 (c= -0.14256414)

		STATE 2: E= 0.138468 au 3.768 eV 30390.1 cm**-1 <S**2> = 0.000000<br>125a -> 127a : 0.972326 (c= -0.98606570)<br>125a -> 129a : 0.019537 (c= -0.13977309)<br><br>STATE 3: E= 0.142688 au 3.883 eV 31316.4 cm**-1 <S**2> = 0.000000<br>123a -> 127a : 0.106187 (c= -0.32586339)<br>123a -> 129a : 0.016953 (c= 0.13020313)<br>124a -> 127a : 0.826608 (c= -0.90917960)
**4d**	1 28114.0 355.7 0.758649112<br>2 32351.5 309.1 0.046029421<br>3 33518.7 298.3 0.105717891	STATE 1: E= 0.128097 au 3.486 eV 28114.0 cm**-1 <S**2> = 0.000000<br>111a -> 112a : 0.971124 (c= -0.98545601)<br><br>STATE 2: E= 0.147404 au 4.011 eV 32351.5 cm**-1 <S**2> = 0.000000<br>109a -> 112a : 0.047261 (c= -0.21739689)<br>109a -> 113a : 0.043346 (c= -0.20819722)<br>109a -> 114a : 0.011932 (c= 0.10923302)<br>110a -> 112a : 0.810595 (c= -0.90033048)<br>111a -> 113a : 0.041884 (c= -0.20465666)<br><br>STATE 3: E= 0.152722 au 4.156 eV 33518.7 cm**-1 <S**2> = 0.000000<br>109a -> 112a : 0.010431 (c= 0.10213395)<br>110a -> 112a : 0.055189 (c= -0.23492372)<br>111a -> 113a : 0.809555 (c= 0.89975297)<br>111a -> 115a : 0.083501 (c= 0.28896486)
**4e**	1 26500.2 377.4 0.707712188<br>2 30574.0 327.1 0.014845948<br>3 31816.2 314.3 0.244345473	STATE 1: E= 0.120744 au 3.286 eV 26500.2 cm**-1 <S**2> = 0.000000<br>135a -> 136a : 0.952880 (c= -0.97615571)<br>135a -> 138a : 0.014714 (c= -0.12130176)<br><br>STATE 2: E= 0.139306 au 3.791 eV 30574.0 cm**-1 <S**2> = 0.000000<br>135a -> 139a : 0.926021 (c= 0.96230006)<br>135a -> 140a : 0.010780 (c= 0.10382794)<br>135a -> 141a : 0.011292 (c= 0.10626289)<br><br>STATE 3: E= 0.144965 au 3.945 eV 31816.2 cm**-1 <S**2> = 0.000000<br>133a -> 136a : 0.034409 (c= 0.18549636)<br>133a -> 138a : 0.023264 (c= -0.15252491)<br>134a -> 136a : 0.789767 (c= 0.88868851)<br>135a -> 136a : 0.013327 (c= 0.11544249)

		135a -> 137a : 0.020598 (c= 0.14351845)<br>135a -> 138a : 0.068068 (c= -0.26089876)
**4f**	1 27847.0 359.1 0.477044445<br>2 31191.7 320.6 0.188438477<br>3 31801.3 314.5 0.000007003	STATE 1: E= 0.126880 au 3.453 eV 27847.0 cm**-1 <S**2> = 0.000000<br>134a -> 135a : 0.935698 (c= 0.96731475)<br>134a -> 136a : 0.021599 (c= -0.14696519)<br>134a -> 137a : 0.018211 (c= 0.13494968)<br><br>STATE 2: E= 0.142120 au 3.867 eV 31191.7 cm**-1 <S**2> = 0.000000<br>131a -> 135a : 0.036993 (c= 0.19233585)<br>131a -> 137a : 0.028225 (c= -0.16800268)<br>132a -> 135a : 0.879045 (c= 0.93757414)<br>132a -> 136a : 0.012680 (c= 0.11260730)<br><br>STATE 3: E= 0.144897 au 3.943 eV 31801.3 cm**-1 <S**2> = 0.000000<br>133a -> 135a : 0.952008 (c= -0.97570880)<br>133a -> 136a : 0.022349 (c= 0.14949546)<br>133a -> 137a : 0.020078 (c= -0.14169529)
**5a**	1 25784.3 387.8 0.925073934<br>2 30712.9 325.6 0.088284344<br>3 34106.1 293.2 0.333398738	STATE 1: E= 0.117482 au 3.197 eV 25784.3 cm**-1 <S**2> = 0.000000<br>103a -> 104a : 0.982155 (c= 0.99103754)<br><br>STATE 2: E= 0.139938 au 3.808 eV 30712.9 cm**-1 <S**2> = 0.000000<br>101a -> 104a : 0.027726 (c= -0.16651031)<br>102a -> 105a : 0.023762 (c= -0.15414900)<br>103a -> 105a : 0.938808 (c= 0.96892086)<br><br>STATE 3: E= 0.155399 au 4.229 eV 34106.1 cm**-1 <S**2> = 0.000000<br>101a -> 104a : 0.256735 (c= -0.50668999)<br>101a -> 105a : 0.043712 (c= -0.20907325)<br>102a -> 104a : 0.546752 (c= 0.73942683)<br>102a -> 105a : 0.060121 (c= -0.24519633)<br>103a -> 105a : 0.015764 (c= -0.12555671)<br>103a -> 107a : 0.022849 (c= -0.15115759)
**5b**	1 24528.2 407.7 0.790599243<br>2 30493.6 327.9 0.087947143<br>3 30565.1 327.2 0.042070589<br>4 32668.7 306.1 0.704585824	STATE 1: E= 0.111759 au 3.041 eV 24528.2 cm**-1 <S**2> = 0.000000<br>127a -> 128a : 0.972238 (c= 0.98602136)<br><br>STATE 2: E= 0.138939 au 3.781 eV 30493.6 cm**-1 <S**2> = 0.000000<br>126a -> 129a : 0.012839 (c= -0.11330900)

		127a -> 129a : 0.686052 (c= 0.82828273)<br>127a -> 130a : 0.225419 (c= 0.47478273)<br>127a -> 131a : 0.041551 (c= 0.20384137)<br><br>STATE 3: E= 0.139265 au 3.790 eV 30565.1 cm**-1 <S**2> = 0.000000<br>127a -> 129a : 0.241908 (c= 0.49184108)<br>127a -> 130a : 0.704401 (c= -0.83928571)<br><br>STATE 4: E= 0.148850 au 4.050 eV 32668.7 cm**-1 <S**2> = 0.000000<br>125a -> 128a : 0.050437 (c= 0.22458200)<br>126a -> 128a : 0.412347 (c= -0.64214241)<br>127a -> 129a : 0.014647 (c= 0.12102642)<br>127a -> 131a : 0.445252 (c= -0.66727181)
**5c**	1 26367.6 379.3 0.483467416<br>2 30393.1 329.0 0.000000727<br>3 32569.2 307.0 0.929865279	STATE 1: E= 0.120139 au 3.269 eV 26367.6 cm**-1 <S**2> = 0.000000<br>126a -> 127a : 0.965679 (c= 0.98268951)<br>126a -> 130a : 0.016847 (c= -0.12979607)<br><br>STATE 2: E= 0.138481 au 3.768 eV 30393.1 cm**-1 <S**2> = 0.000000<br>125a -> 127a : 0.977074 (c= 0.98847078)<br>125a -> 130a : 0.015066 (c= -0.12274525)<br><br>STATE 3: E= 0.148396 au 4.038 eV 32569.2 cm**-1 <S**2> = 0.000000<br>123a -> 127a : 0.070011 (c= -0.26459549)<br>123a -> 128a : 0.020835 (c= -0.14434439)<br>124a -> 127a : 0.768614 (c= 0.87670651)<br>126a -> 128a : 0.012922 (c= -0.11367703)<br>126a -> 129a : 0.069868 (c= -0.26432480)
**5d**	1 26267.4 380.7 0.976773127<br>2 31025.3 322.3 0.083638581<br>3 34178.6 292.6 0.235254227	STATE 1: E= 0.119683 au 3.257 eV 26267.4 cm**-1 <S**2> = 0.000000<br>111a -> 112a : 0.980408 (c= -0.99015576)<br><br>STATE 2: E= 0.141362 au 3.847 eV 31025.3 cm**-1 <S**2> = 0.000000<br>109a -> 112a : 0.038052 (c= -0.19506998)<br>110a -> 113a : 0.028844 (c= 0.16983573)<br>111a -> 113a : 0.922615 (c= -0.96052875)<br><br>STATE 3: E= 0.155729 au 4.238 eV 34178.6 cm**-1 <S**2> = 0.000000<br>109a -> 112a : 0.367505 (c= -0.60622151)

		109a -> 113a : 0.038445 (c= 0.19607512)<br>110a -> 112a : 0.402840 (c= 0.63469680)<br>110a -> 113a : 0.085665 (c= 0.29268636)<br>111a -> 113a : 0.027844 (c= 0.16686426)<br>111a -> 115a : 0.030037 (c= -0.17331191)
**5e**	1 24890.5 401.8 0.844724557<br>2 30534.0 327.5 0.016394603<br>3 30942.4 323.2 0.115123211	STATE 1: E= 0.113409 au 3.086 eV 24890.5 cm**-1 <S**2> = 0.000000<br>135a -> 136a : 0.970602 (c= 0.98519128)<br><br>STATE 2: E= 0.139123 au 3.786 eV 30534.0 cm**-1 <S**2> = 0.000000<br>135a -> 138a : 0.938965 (c= -0.96900201)<br><br>STATE 3: E= 0.140984 au 3.836 eV 30942.4 cm**-1 <S**2> = 0.000000<br>133a -> 136a : 0.020948 (c= -0.14473298)<br>134a -> 137a : 0.024037 (c= -0.15503896)<br>135a -> 137a : 0.902843 (c= -0.95018052)<br>135a -> 139a : 0.023746 (c= -0.15409703)
**5f**	1 26754.5 373.8 0.530879882<br>2 30843.4 324.2 0.000005202<br>3 32596.7 306.8 0.829471138	STATE 1: E= 0.121902 au 3.317 eV 26754.5 cm**-1 <S**2> = 0.000000<br>134a -> 135a : 0.962479 (c= -0.98106036)<br>134a -> 138a : 0.018462 (c= 0.13587420)<br><br>STATE 2: E= 0.140533 au 3.824 eV 30843.4 cm**-1 <S**2> = 0.000000<br>133a -> 135a : 0.974352 (c= 0.98709288)<br>133a -> 138a : 0.017249 (c= -0.13133551)<br><br>STATE 3: E= 0.148522 au 4.041 eV 32596.7 cm**-1 <S**2> = 0.000000<br>131a -> 135a : 0.104886 (c= -0.32386105)<br>131a -> 136a : 0.022135 (c= -0.14877842)<br>132a -> 135a : 0.704476 (c= 0.83933045)<br>132a -> 136a : 0.012422 (c= -0.11145288)<br>134a -> 136a : 0.013011 (c= -0.11406696)<br>134a -> 137a : 0.094205 (c= -0.30692844)

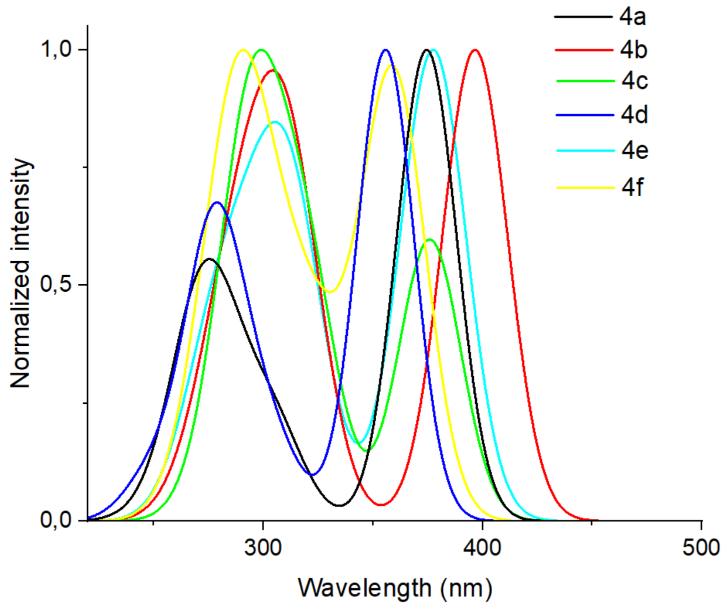

a

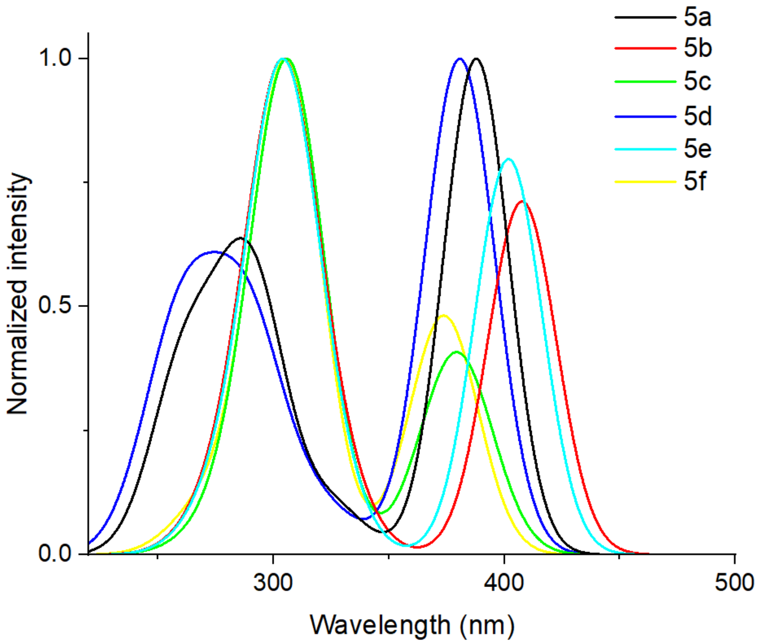

b

Figure S26. Computed absorption spectra for compounds **4a-f** (a) and **5a-f** (b).

Table S20. Hole-electron interaction analysis result for compounds **4**, **5**.

Comp	Sr(a.u.)	D index (Å)	H index (Å)	t index (Å)	Ec	HDI/EDI	Distribution of hole and electron
**4a**	0.50544	6.310	3.719	2.895	3.08	8.81/ 6.80	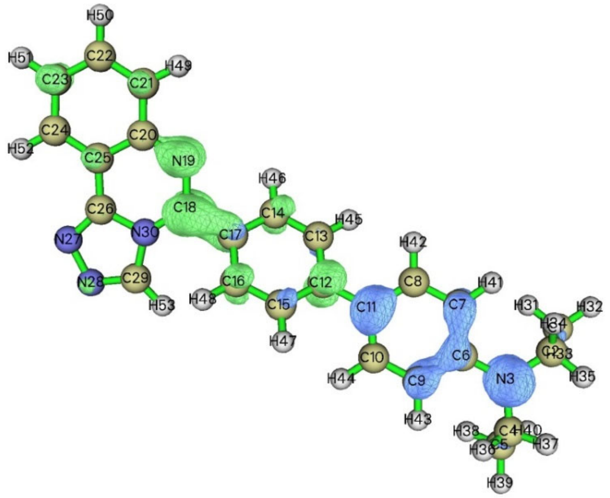
**4b**	0.50926	6.850	4.106	3.163	2.76	8.09/ 5.97	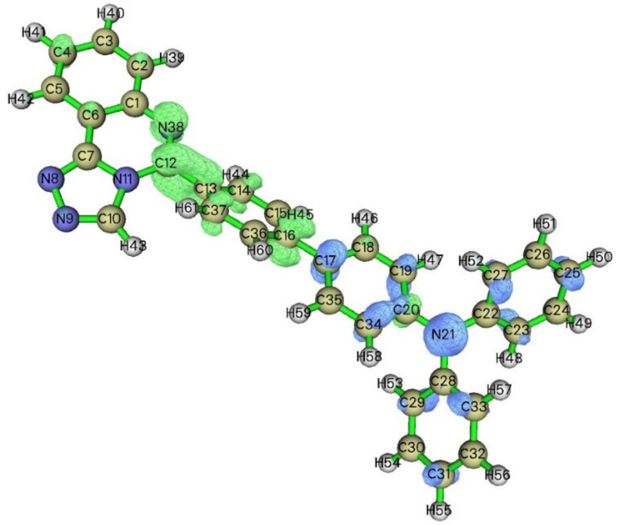

**4c**	0.39654	7.830	3.934	4.350	2.42	7.99/5.85	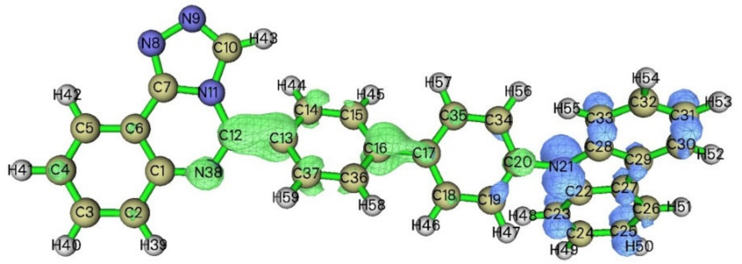
**4d**	0.50724	6.356	3.755	2.951	2.98	8.44/ 6.79	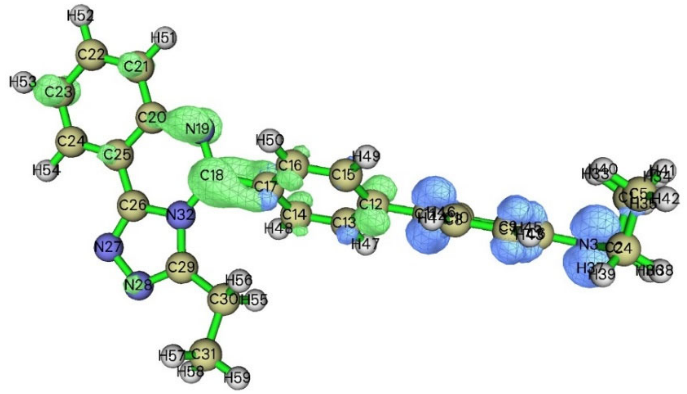
**4e**	0.52832	6.586	4.149	2.880	2.79	8.19/ 5.64	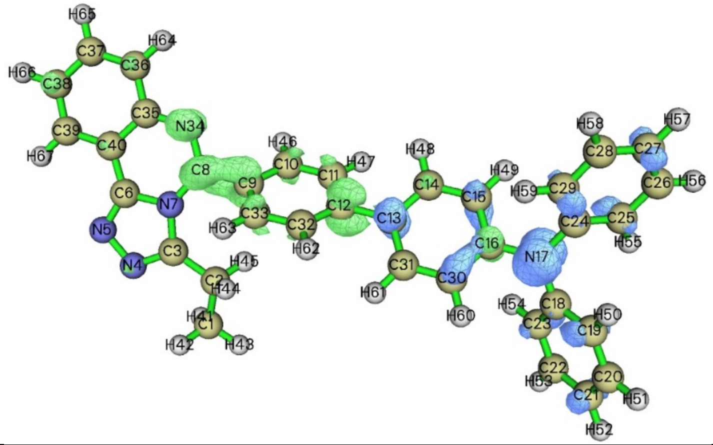

**4f**	0.45366	6.982	4.116	3.333	2.66	7.79/5.61	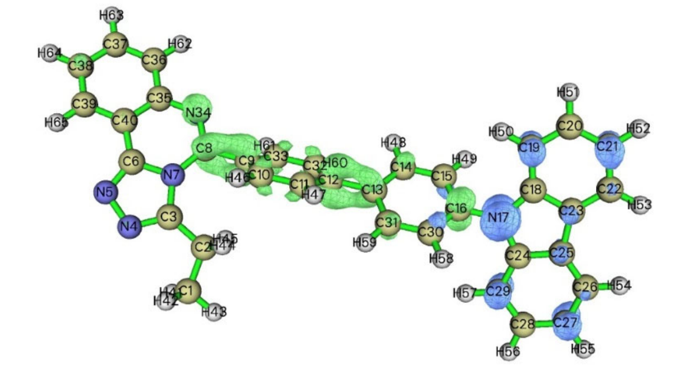
**5a**	0.49229	6.546	3.698	3.176	2.94	8.67/ 6.77	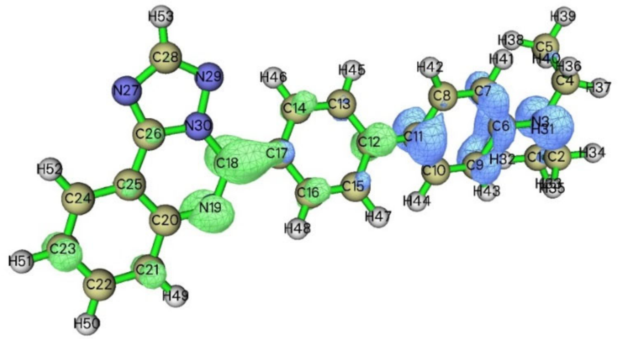
**5b**	0.48785	7.272	4.074	3.620	2.63	8.07/6.11	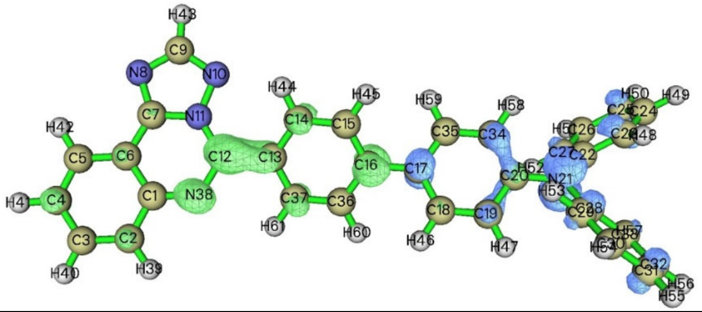

**5c**	0.40714	8.170	3.994	4.616	2.34	7.74/ 5.98	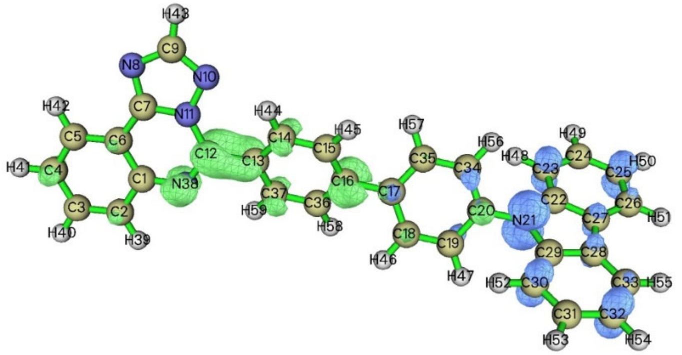
**5d**	0.51262	6.317	3.748	3.026	2.85	8.48/ 6.65	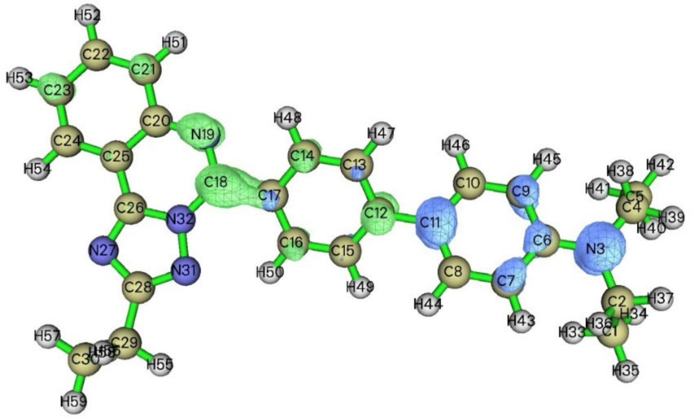

**5e**	0.50296	7.018	4.104	3.406	2.61	8.06/6.03	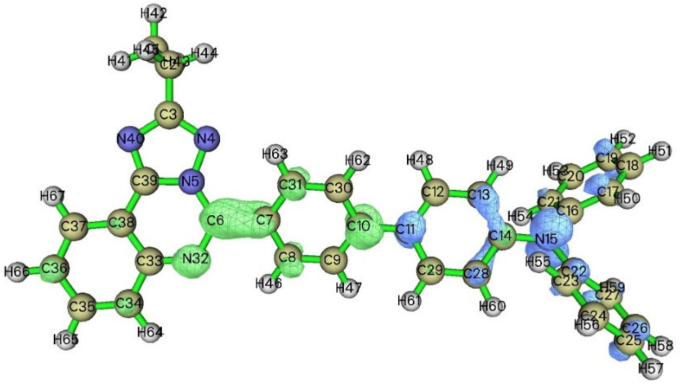
**5f**	0.42992	7.809	4.068	4.233	2.38	7.82/5.83	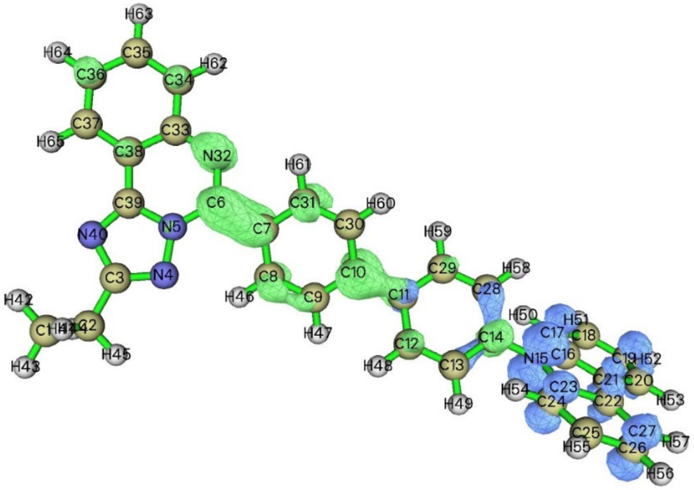

[1] V. A. Mamedov, N. A. Zhukova, and M. S. Kadyrova, “The Dimroth Rearrangement in the Synthesis of Condensed Pyrimidines – Structural Analogs of Antiviral Compounds,” *Chem. Heterocycl. Compd.*, vol. 57, no. 4, pp. 342–368, Apr. 2021, doi: 10.1007/s10593-021-02913-7.
